# Health impacts of electronic nicotine delivery systems: an umbrella review of systematic reviews

**DOI:** 10.1136/bmjopen-2025-100168

**Published:** 2025-10-10

**Authors:** Jagdish Kaur, Sonu Goel, Muhammed Shabil, Rishabh Kumar Rana, Arvind Vashishta Rinkoo, Anil Chauhan, Shibaji Gupta

**Affiliations:** 1 World Health Organization Regional Office for South‒East Asia, New Delhi, India; 2 School of Medicine, Faculty of Education & Health Sciences, University of Limerick, Limerick, Ireland, Ireland; 3 Department of Community Medicine and School of Public Health, Postgraduate Institute of Medical Education and Research, Chandigarh, India; 4 Department of Pharmacy Practice, Faculty of Pharmacy, MS Ramaiah University of Applied Sciences, Bangalore, Karnataka, India; 5 Department of Community Medicine, Shaheed Nirmal Mahto Medical College and Hospital, Dhanbad, India; 6 Department of Telemedicine, Post Graduate Institute of Medical Education and Research, Chandigarh, India; 7 Department of Community Medicine, Midnapore Medical College, Midnapore, West Bengal, India

**Keywords:** PUBLIC HEALTH, Substance misuse, Smoking Reduction

## Abstract

**Abstract:**

**Background:**

The rise of electronic nicotine delivery systems (ENDS) has introduced new challenges to tobacco control and regulation, particularly among young adults, raising questions about their safety. This umbrella review aimed to synthesise existing systematic reviews with or without meta-analyses to evaluate the health impacts of ENDS.

**Methods:**

We conducted a systematic literature search via the PICO strategy across multiple databases, focusing on e-cigarettes, ENDS and e-liquids, while excluding non-nicotine e-cigarette and nicotine replacement therapies (NRTs). Health outcomes include a range of clinical diseases and physiological changes. Quality assessment was performed via assessing the methodoligcal quality of systematic reviews 2 (AMSTAR-2), and the findings were synthesised narratively and in tables, prioritising the highest-rated reviews. The meta-analyses used R software (V.4.3) random effects models, and evidence quality was assessed via the Grading of Recommendations, Assessment, Development and Evaluation criteria.

**Results:**

Of the 5055 records, 69 systematic reviews were included. Systematic reviews have indicated increased risks of cardiovascular and respiratory diseases, mental health issues and substance abuse with ENDS use, especially among adolescents. Cardiovascular risk factors included increased heart rate (mean difference (MD) 1.41, 95% CI 0.81 to 2.01, I^2^=91%) from 25 studies; increased blood pressure (MD for systolic blood pressure=0.51 mm Hg, 95% CI 0.26 to 0.75, I^2^=89%; MD for diastolic blood pressure=0.59 mm Hg, 95% CI 0.35 to 0.83, I^2^=82%) from 23 studies; endothelial dysfunction and increased platelet activity. Respiratory risk factors included reduced lung function and a higher incidence of asthma in nine studies (OR 1.30, 95% CI 1.1 to 1.55; I^2^=43%) and chronic obstructive pulmonary disease. Mental health concerns, such as depression and suicidality, were also prevalent among adolescent ENDS users. Nine studies reported a negative effect of ENDS on periodontal health. Evidence of carcinogens has been found in the urinary examinations of ENDS users in some studies. The adverse events reported in seven randomised controlled trials with 2611 participants were similar between ENDS and NRT (RR 1.13, 95% CI 0.83 to 1.54, I^2^=12%).

**Conclusions:**

Exposure to ENDS is harmful to various organ systems, especially cardiovascular and respiratory systems. Comprehensive regulatory measures and public health strategies are necessary to curb the use of ENDS, particularly among young people.

**STRENGTHS AND LIMITATIONS OF THIS STUDY**

- A comprehensive umbrella study consolidating various physiological health impacts of electronic nicotine delivery systems (ENDS) derived from existing meta-analyses.
- High statistical power and accuracy for critical cardiac outcomes due to a substantial number of contributing studies.
- Notable heterogeneity, affected by diverse confounding adjustments and insufficient data for impact modification analysis.
- Restricted statistical power for certain outcomes, particularly those employing less robust dichotomous measures or involving a smaller number of included studies.
- The scope is confined to nicotine-containing ENDS, potentially excluding adverse consequences from non-nicotine constituents.

## Introduction

 Tobacco remains the most significant preventable cause of mortality worldwide and is responsible for approximately 8 million deaths annually. It significantly increases the risk of cancer, heart disease and stroke.^1^ Furthermore, secondhand smoke accounts for the deaths of 1.2 million people each year.^2^ A recent study estimated that over 50 billion years of life were lost as a result of tobacco-related premature mortalities.^3^ In 2019, tobacco-related disorders constituted 6.7% of the global healthcare costs and 1.4% of the gross domestic product,^4 5^ with healthcare expenses surpassing US$300 billion in the USA alone, alongside a productivity loss of US$150 billion.^4 5^

In addition to tobacco, nicotine is another culprit that leads to cardiovascular diseases, such as coronary heart disease, stroke, heart failure and peripheral arterial disease.^6^,^8^ Nicotine stimulates the sympathetic nervous system, which causes narrowing of the coronary arteries, decreasing heart flow reserve, along with temporarily increasing heart rate, blood pressure and heart contractility.^9^,^12^ Additionally, nicotine can lead to airway diseases such as asthma, chronic bronchitis and chronic obstructive pulmonary disease (COPD).^13^

Electronic nicotine delivery systems (ENDS) are battery-powered devices that heat a liquid solution into aerosol that is inhaled by the user. ENDS are being aggressively marketed as reduced-harm alternatives to traditional cigarettes. On the contrary, they still pose serious health concerns, particularly for vulnerable age groups, because of the presence of various toxic substances like nicotine, heavy metals such as nickel, tin and lead; volatile chemical compounds and carcinogens in the aerosol.^14 15^ The levels of heavy metals in ENDS’ aerosol are reportedly higher than those in traditional cigarette smoke.^16^ The literature has documented that ENDS can lead to periodontal disease, tissue damage, toxicity and sudden heart rate irregularities.^17 18^ The Food and Drug Administration has reported the side effects of ENDS, such as seizures, tremors, fainting and severe neurological symptoms. ENDS also impairs brain development.^19 20^ The adverse effects of ENDS consumption are not limited to the cardiovascular and respiratory systems but extend to various other organ systems.^20^ The long-term effects of hazardous aldehydes produced by heating propylene glycol and glycerine in e-liquids remain unknown. The current situation demands a definitive assessment of the health risks associated with ENDS, particularly their impact on the cardiorespiratory system.

The prevalence of ENDS use in adults in the USA was estimated to be ~11.1 million (4.5%) in 2021.^21^ In the UK, the use of e-cigarettes has shown notable trends in recent years. As of 2024, approximately 8.6% of adults in England reported using e-cigarettes, with a significant increase observed among younger adults.^22^ The variety of flavours in disposable ENDS, including fruity and dessert-like options, along with discreet designs, plays a crucial role in attracting younger users.^23 24^

Numerous systematic reviews have highlighted the range of adverse health outcomes associated with ENDS. However, few reviews have reported uncertain outcomes, which limited the understanding about ENDS to guide policymakers and implementors. An umbrella review offers a bird’s-eye view by synthesising existing systematic reviews and meta-analyses.^25^ In the recent past, three umbrella reviews reported health outcomes. An umbrella review by Afsar *et al*^26^ included 40 systematic reviews reporting health outcomes. Khan *et al*^27^ compiled six systematic reviews in their umbrella review on vaping and mental health conditions in children.^27^ The most recent review by Banks *et al*^28^ collated the results of eight major independent systematic reviews (2017–2020), and the 2018 US National Academies of Sciences, Engineering and Medicine report. These umbrella reviews reported uncertain health outcomes for ENDS. Further, they are often constrained by their limited scope and the quantity of evidence considered.

The current umbrella review builds on previous work by (1) including a substantially larger evidence base, incorporating 69 systematic reviews—a significant increase over the 40 included by Afsar *et al*; (2) synthesising recent meta-analytical data to provide updated, quantitative risk estimates for key cardiovascular and respiratory outcomes and (3) applying the rigorous Grading of Recommendations, Assessment, Development and Evaluation (GRADE) criteria to formally assess the quality of evidence for each major health outcome, offering a clear guide for clinical and policy decisions. Thus, the current umbrella review provides a broader and more current appraisal of ENDS-related health risks for a comprehensive analysis across various health outcomes (immediate and long-term impacts), enhancing our comprehension of their risks and assisting in developing informed, evidence-based health policies and practices.

This umbrella review is part of a trilogy derived from a comprehensive protocol registered on PROSPERO CRD42023464207 that has already been published as Goel *et al.*^29^ This included safety, efficacy, gateway effect and health outcomes with ENDS use. However, owing to the comprehensiveness of the topic and the vast number of recent studies on subtopics, we have divided these into three distinct umbrella reviews. This paper specifically focuses on synthesising acute and long-term adverse health-related impacts of ENDS across major organ systems.

## Methods

This umbrella review was conducted via the approach recommended by the Joanna Briggs Institute.^25^ Throughout this process, we adhered to the Preferred Reporting Items for Overviews of Reviews (PRIOR) guidelines.^30^ A checklist derived from Preferred Reporting Items for Systematic Reviews and Meta-Analyses (PRISMA) was constructed and is available in online supplemental table S1.

### Inclusion and exclusion criteria

We included studies that investigated the health ENDS. Our umbrella review comprehensively included systematic reviews, both with and without meta-analysis, that evaluated any adverse health outcomes, both serious and non-serious, associated with the use of ENDS. The severe adverse events (SAEs) included medical events that could lead to death, were life-threatening, required hospitalisation or resulted in significant disability. The focus was on both immediate effects, such as changes in cardiac and respiratory parameters, and long-term effects, such as the development of diseases, including stroke, COPD and asthma. In terms of exposure or intervention, the reviews included those on ENDS and e-liquids. Studies exclusively investigating non-nicotine vaping products (NNVP), non-nicotine ENDS and other pharmaceutical treatments, such as nicotine replacement therapy (NRT), were excluded from the meta-analysis. The latter were excluded as we need to focus on nicotine-related health risks from ENDS to ensure consistency with the review’s objectives and avoid bias in health effects from inclusion of non-nicotine products. Table 1 outlines the inclusion and exclusion criteria adopted for this umbrella review.

**Table 1:** PICO

PICO	Inclusion criteria	Exclusion criteria
Population	General population with or without cigarette smokers with >12 years age	Animals In vitro In vivo
Intervention	E-cigarettes, electronic nicotine delivery systems, e-liquids	Nicotine replacement therapy Non-nicotine e-cigarettes Other pharmacological interventions
Comparison	Placebo e-cigarette (without nicotine) or any comparator treatment or combination of treatments usually given for smoking cessation, for example, nicotine replacement therapy Never smokers (no e-cigarette or combustible tobacco products ever)	Dual users of e-cigarette and tobacco
Outcome	Primary Outcomes: Clinical disease endpoints, such as myocardial infarction, coronary artery disease, congestive heart failure, stroke, other cardiovascular disease and cancer. Development of risk factors and intermediate biological effect of health outcomes like atherosclerosis, high blood pressure, lung damage, high glucose levels, dyslipidaemia. Respiratory diseases oral health, renal health, neurological effects, optical health, wound healing, olfactory, endocrine, allergic diseases and haematological outcomes. Effect on pregnancy, neonatal effects, development and reproductive effects. Mental health, effects on sleep pattern, quality, duration. Nicotine dependency. Serious and non-serious adverse effects.	Economic outcomes Environmental outcomes
Study type	Systematic reviews and meta-analyses of RCTs and observational studies Primary studies (observational studies and RCTs)	Case reports, non-human studies
Setting	Any country or setting	No exclusion
Follow-up	No restrictions	No exclusion
Language	English	Not available in English

RCTs, randomised controlled trials.

### Literature search

A skilled medical librarian conducted an exhaustive literature search from inception to January 2024 using various databases, including OVID (Medline), PubMed, EMBASE, Scopus, CINAHL, the Cochrane Library and Web of Science. Using the PICO strategy, the search included a combination of keywords and MeSH terms related to e-cigarettes (eg, “electronic cigarettes,” “electronic nicotine delivery systems,” “vaping,” “e-liquids”) and the population (eg, “general population,” “adolescents,” “adults”). Additionally, the reviewers examined citations within the identified publications to identify more pertinent articles. The search was limited to human studies published in English. The search methodology is provided in online supplemental table S2.

### Screening and selection

The search results were exported to Mendeley for duplicate removal. The initial screening of titles and abstracts was conducted via Rayyan software, followed by full-text review in Excel. To assess eligibility, two independent reviewers (MS and AC) evaluated the titles, abstracts and full texts of the papers. The analysis included systematic reviews that met predefined PICO criteria. In instances of disagreement between the two reviewers, a third reviewer (SG) assessed the article to reach consensus and make a final decision on its inclusion or exclusion.

### Data extraction

Data extraction was carried out by five reviewers (RKR, PS, SoG, ShG and CS) through a prepiloted and standardised data extraction form. The extracted data were verified by an impartial reviewer (MS). In cases of discrepancies in data extraction, a third reviewer (AC) was consulted to facilitate discussion and reach a consensus. Quantitative and qualitative data were collected from each study. The compilation of quantitative results included critical details such as the name of the primary author, publication year, types of studies included, number of randomised controlled trials (RCTs) and observational studies in the review, characteristics of study participants, specifics of interventions and comparators, and outcomes evaluated. The extracted information included the total number of participants, effect sizes with CIs, metrics and outcomes related to heterogeneity, results concerning publication bias, and the tests used, as well as details of funding and the risk of bias. Additionally, values for the overall combined effects, Egger’s test and I^2^ were obtained. Funding sources for the systematic reviews were identified, with particular attention given to any potential conflicts of interest, especially financial incentives related to the intervention.

### Quality assessment

Five reviewers used the AMSTAR-2 (assessing the methodoligcal quality of systematic reviews 2) scale^31^ to assess the quality of the included systematic reviews. AMSTAR-2 consists of 16 domains, 7 of which are deemed critical owing to their significant impact on trust in systematic review conclusions. These critical domains encompass aspects such as review protocol registration, appropriateness of the search strategy, reasons for excluding specific studies, risk of bias assessment in included studies and its impact on systematic review conclusions, evidence synthesis methods and considerations for publication bias. The overall level of confidence in the findings of the systematic review was classified as high, moderate, low or critically low.^31^

### Data synthesis

The data of each systematic review included in our analysis were extracted and meta-analysis performed when the same outcome is reported by multiple systematic reviews, each with different included primary studies and when sufficient data are available. The meta-analysis integrated effect sizes from each trial, employing a fixed effects model for studies with low heterogeneity and a random effects model for those with high heterogeneity. This analysis was conducted via the ‘meta’ and ‘metafor’ packages in R software, V.4.3.^32^ Subgroup analyses were performed, considering variables such as participant characteristics, outcomes, comparators and other potential contributors to heterogeneity. Heterogeneity was assessed via the I² statistic. The scores were graded as follows: 0%–40%, low heterogeneity; 30%–60%, moderate heterogeneity; 50%–90%, substantial heterogeneity and 75%–100%, considerable heterogeneity.^33^ Statistical significance was set at p<0.05. Publication bias was evaluated by visual inspection of funnel plot symmetry, the trim-and-fill procedure and Egger’s test. However, publication bias was not assessed for meta-analysis with fewer than 10 included studies, due to the limited reliability of these tests in this scenario.

The quality of evidence was appraised through the GRADE criteria. It focuses on five domains: risk of bias in individual studies, inconsistency, indirectness, imprecision and publication bias for each outcome.^34^ The GRADE assessment was conducted via the GRADE pro web application which categorises the strength of evidence into four levels. When evidence is considered ‘very low’, it indicates a strong likelihood that the actual effect diverges significantly from the estimated effect. ‘Low’ suggests that the actual effect could be markedly different from what was estimated. For evidence categorised as ‘moderate’, there is a belief among authors that the actual effect is likely close to the estimated effect. Finally, evidence deemed ‘high’ reflects a high degree of confidence from the authors in the similarity between the true and estimated impacts.^35^

## Results

### Search results

**Figure 1:**
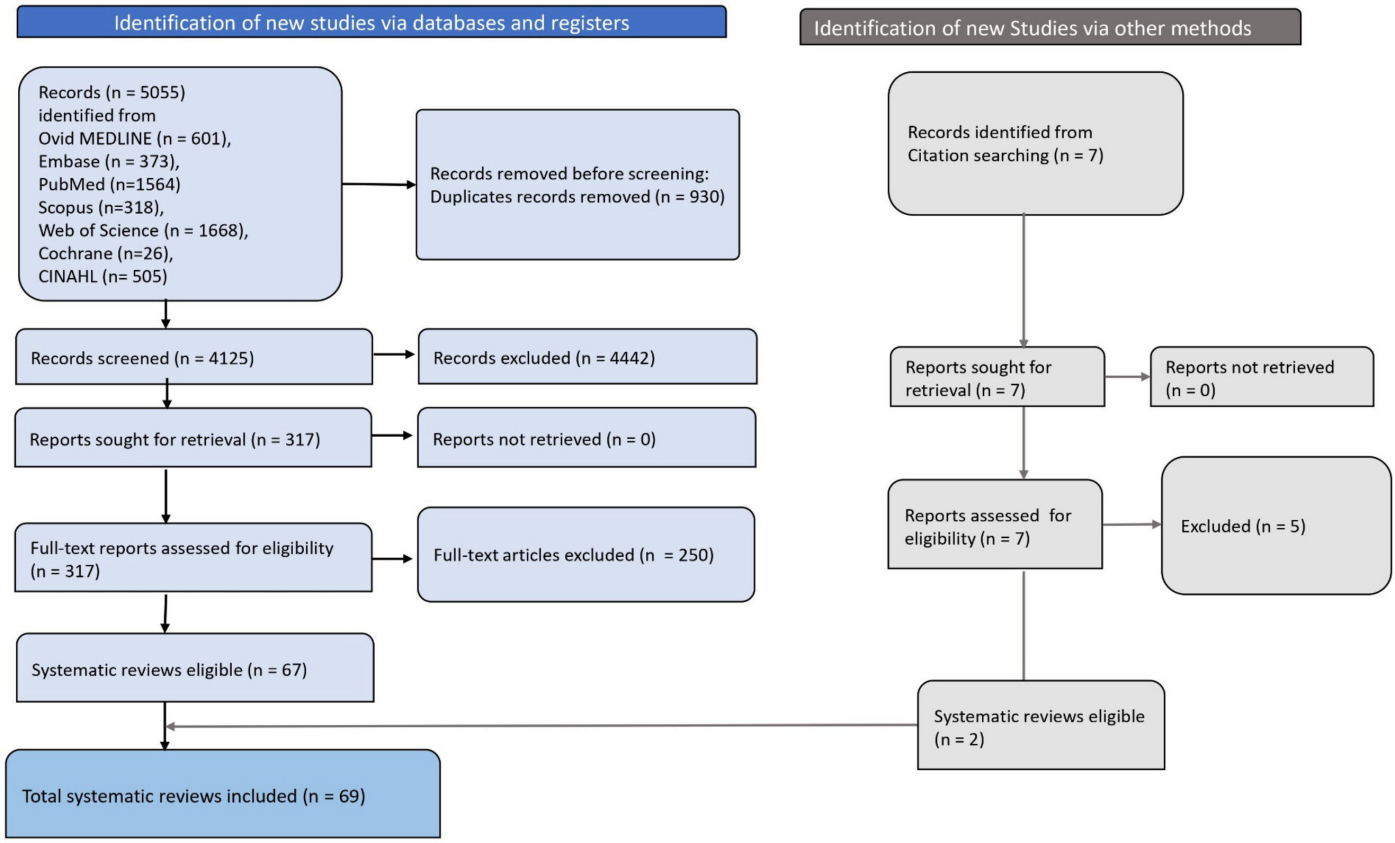
PRISMA flow chart depicting the screening and selection process of the article. PRISMA, Preferred Reporting Items for Systematic Reviews and Meta-Analyses.

In total, 5055 records were identified from various databases. After removing duplicates (n=930), excluding studies in the primary screening (n=4242) and full screening (n=250) for various reasons, 69 systematic reviews and meta-analyses were included in the umbrella review (online supplemental table S4).^1017 36^,^102^ Figure 1 illustrates the PRISMA flow chart of the screening process employed for the umbrella review.

### Characteristics of the included systematic reviews

The resulting set of systematic reviews uses a variety of study types to investigate the impact of ENDS on various populations worldwide. Between 2013 and 2024, researchers investigated various health outcomes, including respiratory and cardiovascular effects, mental health and dental health, across a range of age groups, including adolescents and pregnant women. While study quality and bias varied, the majority reported no conflicts of interest and received funding from sources, such as government grants and health organisations. Online supplemental table S3 summarises the key characteristics of the included reviews, table 2 shows the overall results from the RCTs along with publication bias estimates, table 3 shows the pooled results reported in the systematic reviews and meta-analyses based on observational studies and online supplemental table S5 displays the quality assessment of the reviews. The forest plots are given as online supplemental figures S1–S7. Funnel plots and trim fill results are given in online supplemental figures S8–S10.

**Table 2:** Summary details of the meta-analysis of the RCTs

Comparison	No of studies	E-cigarette sample size	Comparison group sample size	Type of effect size (ES)	ES (95% CI)	I^2^	Publication bias†	GRADE
E-cigarettes vs NRT	6	1016	959	RR	0.99 (0.873 to 1.125)	17%	NA	Moderate
E-cigarettes vs non-nicotine e-cigarettes	5	513	327	RR	1.01 (0.915 to 1.11)	0%	NA	Low
E-cigarettes vs NRT	7	1340	1271	RR	1.13 (0.83 to 1.54)	12%	NA	Low
E-cigarettes vs non-nicotine e-cigarettes	9	859	553	RR	1.00 (0.55 to 1.80)	0%	NA	Low
Heart rate (pre vs post e-cigarette)	23	750	750	MD	1.41 (0.81 to 2.01)**	91%	Yes (Egger test p value=0.008)	NA
SBP (pre vs post e-cigarette)	23	544	544	MD	0.51 (0.26 to 0.75)**	89%	No (Egger test p=0.370)	NA
DBP (pre vs post e-cigarette)	23	544	544	MD	0.59 (0.35 to 0.83)**	82%	Yes (Egger p=0.0325)	NA
MAP (pre vs post e-cigarette)	5	191	191	MD	5.17 (3.33 to 7.02)**	74%	NA	NA
FMD (pre vs post e-cigarette)	3	157	157	MD	0.78 (−0.08 to 1.64)	84%	NA	NA
PWV (pre vs post e-cigarette)	3	70	70	MD	0.60 (0.25 to 0.94)**	95%	NA	NA
Alx-75 (pre vs post e-cigarette)	4	90	90	MD	0.58 (0.22 to 0.94)**	75%	NA	NA
Soluble CD40 (pre vs post e-cigarette)	2	60	60	MD	1.14 (0.80 to 8.66)*	98%	NA	NA
Soluble P-selectin (pre vs post e-cigarette)	2	60	60	MD	4.73 (0.46 to 1.13)*	92.60%	NA	NA
FEV1 (pre vs post e-cigarette)	6	132 (Pre)	132 (Post)	SMD	−0.15 (−0.32 to 0.01)	20.80%	NA	NA
FEV1 (e-cigarette vs support/counselling)	2	498	216	SMD	0.15 (−0.01 to 0.31)	70%	NA	NA
FVC (pre vs post e-cigarette)	5	147 (Pre)	147 (Post)	SMD	−0.05 (−0.22 to 0.12)	0%	NA	NA
FEV1/FVC (pre vs post e-cigarette)	6	167 (Pre)	167 (Post)	SMD	−0.05 (−0.31 to 0.22)	65%	NA	NA
FeNO (pre vs post e-cigarette)	10	257 (Pre)	257 (Post)	SMD	−0.27 (−0.56 to −0.01)	85.40%	NA	NA
FEV1/FVC (e-cigarette vs NRT)	2	46	35	MD	−0.16 (−1.83 to 1.50)	51%	NA	NA

*p<0.05 and **p<0.00.

†Not assessed (NA) for meta-analysis with less than 10 included studies, due to limited reliability of relevant tests in these cases.

DBP, diastolic blood pressure; FeNO, fractional exhaled nitric oxide; FEV1, forced expiratory volume in one second; FMD, flow-mediated vasodilation; FVC, forced vital capacity; GRADE, Grading of Recommendations, Assessment, Development and Evaluation; MAP, mean arterial pressure; MD, mean difference; NRT, nicotine replacement therapy; RCTs, randomised controlled trials; RR, relative risk ; SBP, systolic blood pressure; SMD, standardised mean difference.

**Table 3:** Summary details of the results from the meta-analysis of observational studies

Study	Population/comparison	Number of studies	Type of ES	ES	95% CI	P value	I^2^	Publication bias
Asthma								
Xian *et al*, 2021^100^	Current or former e-cigarette users vs never e-cigarette users	15	OR	1.27	1.17 to 1.37	0.0001	45%	Egger’s test p=0.02, trim and fill adjusted the bias
	Current e-cigarette users vs non-e-cigarette users	9	OR	1.3	1.17 to 1.45	0.0001	43%	NA
	Former e-cigarette users vs non-e-cigarette users	6	OR	1.22	1.08 to 1.39	0.0016	48%	NA
	Current e-cigarette users without tobacco smoking vs non-e-cigarette users	4	OR	1.47	1.13 to 1.91	0.004	82%	NA
Wills *et al*, 2021^98^	E-cigarette users vs non-e-cigarette users	15	aOR	1.39	1.28 to 1.51	<0.0001	50%	Not assessed
Chand *et al*, 2022^46^	Current e-cigarette users vs non-e-cigarette users	13	OR	1.36	1.21 to 1.52	<0.0001	73%	Detected in funnel plot
	Current e-cigarette users with never tobacco smoking vs non-e-cigarette users	4	OR	1.62	1.13 to 2.31	0.008	NA	NA
	Ever e-cigarette use vs non-e-cigarette users	7	OR	1.24	1.13 to 1.36	<0.0001	6%	Detected in funnel plot
Li *et al*, 2022^71^	Adolescents e-cigarette users vs non-e-cigarette users	10	OR	1.31	1.22 to 1.42	<0.0001	80%	Not detected (Egger p=0.25)
	Adolescents E-cigarette users (current use) vs Non-E-cigarette users	7	OR	1.36	1.26 to 1.48	<0.0001	61%	NA
	Adolescents E-cigarette users (ever use) vs non-e-cigarette users	6	OR	1.2	1.12 to 1.28	<0.0001	19%	NA
	COPD/composite respiratory symptoms							
Wills *et al*, 2021^98^	E-cigarette users vs non-e-cigarette users	9	aOR	1.49	1.36 to 1.65	<0.0001	0%	NA
	Stroke							
Awad *et al*, 2023^38^	Current e-cigarette users vs non-E-cigarette users	6	OR	1.52	1.17 to 1.97	0.002	80%	NA
	Former e-cigarette users vs non-E-cigarette users	3	OR	1.03	0.87 to 1.21	0.74	63%	NA
Zhao *et al*, 2022^102^	E-cigarette users vs neither e-cigarette nor combustible cigarette users	3	OR	1.13	0.99 to 1.29	0.07	46%	NA

COPD, chronic obstructive pulmonary disease; ES, effect size; NA, not assessed.

### General adverse events

For the risk of SAEs, there was no statistically significant difference between ENDS and either type of comparator (viz. NRTs (RR 1.13, 95% CI 0.83 to 1.54), and non-nicotine e-cigarettes. Similarly, for non-harmful adverse events, the pooled relative risk (RR) for ENDS compared with NRT was 0.99 (95% CI 0.873 to 1.125), whereas ENDS compared with non-nicotine e-cigarettes yielded an RR of 1.01 (95% CI 0.91 to 1.11), which also signifies a statistically non-significant risk among ENDS users compared with their counterparts. The heterogeneity was generally low between comparisons (I^2^ range: 0%–17%), but a low certainty of evidence was consistently found.

### Effects on the cardiovascular and cerebrovascular systems

#### Cardiac parameters

Studies evaluating the impact of ENDS on cardiac parameters were mostly of critically low quality.^10 17 38 43 48 54 57 61 64 65 67 69 70 75 83 85 88 89 102^

The analysis revealed that ENDS use was associated with significant increases in several haemodynamic parameters, with measurements typically taken immediately after use up to 1-hour postexposure. Compared with prevaping baselines, ENDS use increased heart rate by 1.41 bpm (95% CI 0.81 to 2.01; I²=91%), systolic blood pressure (SBP) by 0.51 mm Hg (95% CI 0.26 to 0.75; I²=89%) and diastolic blood pressure (DBP) by 0.59 mm Hg (95% CI 0.35 to 0.83; I²=82%). Additionally, ENDS use significantly elevated mean arterial pressure (5.17 mm Hg; I²=74%), pulse wave velocity (0.6 m/s) and augmentation index at 75 bpm (0.58%), though with considerable heterogeneity across studies. Flow-mediated dilation showed no significant change (MD: 0.78%; I²=84%). At the molecular level, ENDS exposure significantly increased markers of endothelial activation and platelet aggregation, including soluble CD40 ligand (MD=1.14) and soluble P-selectin (MD=4.73), though these findings were also characterised by substantial between-study variability.

#### Stroke

ENDS use was associated with an increased risk of stroke (OR 1.25, 95% CI 1.1 to 1.55).^58 103^ Current use showed a stronger association (OR 1.52, 95% CI 1.17 to 1.97) (58), but another analysis revealed that this association was not statistically significant (OR 1.03, 95% CI 0.87 to 1.21). Compared with non-users of ENDS and combustible tobacco, ENDS users had a significant association with stroke risk (OR 1.13, 95% CI 0.99 to 1.29).^103^

### Effects on the respiratory system

17 systematic reviews discussed the effects of ENDS in respiratory studies.^42^,^4446 57 61 63 65 70 71 82 90 94 98 100^

#### Pulmonary parameters

When respiratory outcomes associated with ENDS use are examined, a variety of pulmonary function tests, including forced expiratory volume in one second (FEV1), forced vital capacity (FVC), the FEV1/FVC ratio and fractional exhaled nitric oxide (FeNO), are considered.

Pulmonary function tests revealed minimal changes after the use of ENDS. Compared with support/counselling, the value of FEV1 slightly but non-significantly decreased (standardised mean differnece (SMD)=−0.15; I^2^=20.8%), and the difference was not significant (SMD=0.15; I^2^=70%). FVC remained unchanged (SMD=−0.05; I^2^=0%). FEV1/FVC did not significantly differ between pre-ENDS and post-ENDS (SMD=−0.05; I^2^=65%) or NRT (MD=−0.16; I^2^=51%). FeNO showed a small reduction (SMD=−0.27; I^2^=85.4%).

#### Risk of asthma

ENDS use was significantly associated with increased asthma risk (OR 1.30, 95% CI 1.17 to 1.45). This risk remained elevated even after cessation (OR 1.22, 95% CI 1.08 to 1.39). Never-tobacco cigarette users using ENDS also presented an increased risk (OR 1.47 to 1.62).^66 104^ Adolescents who use ENDS are also at increased risk (OR=1.31 to 1.46) for asthma,^71 100^ highlighting a consistent pattern of elevated asthma risk across multiple studies. Moreover, ENDS use was associated with increased asthma risk (OR~1.20) in both the general population and adolescents. ENDS use was associated with an increased incidence of COPD or composite respiratory symptoms (OR 1.49, 95% CI 1.36 to 1.65).

#### Adverse events

The present analysis examined adverse events associated with ENDS compared with NRT and non-nicotine ENDS. A total of 13 systematic reviews were included in the analysis, ten of which were of critically low quality.^3651 55 59 61^,^63 73 76 77 80 85 95 97^

#### General adverse events

Six RCTs (1975 participants) reported no significant difference in general adverse events between ENDS and NRT (RR 0.99, 95% CI 0.873 to 1.125; I^2^=17%). Similarly, five RCTs (840 participants) reported no difference between ENDS use and non-nicotine e-cigarette use (RR 1.01, 95% CI 0.91 to 1.11; I^2^=0%).

##### Serious adverse events

Seven studies (2611 participants) revealed no statistically significant difference in serious adverse events between ENDS and NRT (RR 1.13, 95% CI 0.83 to 1.54; I^2^=12%), although the potential for increased risk with ENDS requires consideration. Nine studies (1412 participants) reported no difference in serious adverse events between ENDS use and non-nicotine e-cigarette use (RR 1.00, 95% CI 0.55 to 1.80; I^2^=0%).

### Genitourinary effects

Few studies are available on the genitourinary effects of ENDS use, as only two systematic reviews could be used, one each having low and critically low quality. No specific country was reported by these reviews.^39 41^

#### Bladder cancer

A study indicated that ENDS users may have a greater risk of developing bladder cancer (OR 3.831, 95% CI 0.494 to 29.701) and are diagnosed earlier than those who never use ENDS.^105^ ENDS users also present higher levels of urinary metabolites and carcinogens than non-smokers and non-ENDS users do.^39^

#### Chronic kidney disease

ENDS use may be associated with progression to chronic kidney disease (CKD), although there is no strong evidence available, with higher albuminuria values (an indicator of CKD) observed in ENDS users (160 mg/L (150 to 207.5)) than in both combustible cigarette users (115 mg/L (60; 200)) and non-smokers/non-ENDS users (20 mg/L (10 to 50)) (p<0.01).^106^

#### Sperm characteristics

Compared with non-ENDS users, daily ENDS users have been found to have significantly lower total sperm counts (91.8 million vs 147 million) (p<0.01).^39^ Compared with those in the control group, exposure to ENDS was associated with a notable decrease in sperm concentration, motility and progression, with statistical significance (p<0.01).^107^

#### Urinary components

In terms of chemical exposure, one study identified 40 parent compounds and four metals in the urine of ENDS users. This included 12 compounds in IARC group 1, 4 in group 2A, 8 in group 2B, 8 in group 3 and 10 that were not listed in the IARC monographs, with none in group 4.^41^

### Oral and periodontal effects

Nine systematic reviews reported on these effects, with seven of them evaluated as having low or critically low quality. Studies from India, China, Middle Eastern countries, South Africa and parts of Europe were included.^47 49 60 79 84 92 93 101^

#### Gum disease and bone loss

ENDS users are more likely to experience dental problems (OR 1.28, 95% CI 1.07 to 1.54),^108^ gum disease (OR 1.76, 95% CI 1.12 to 2.76),^109^ bone loss around teeth (OR 1.67, 95% CI 1.06 to 2.63)^109^ and broken teeth (OR 1.65, 95% CI 1.19 to 2.27)^110^ than never-users are.^43^ They also experience increased bone loss around dental implants, greater inflammation, greater plaque accumulation and deeper probing depths.^43^

#### Periodontal parameters

Some studies have shown that vaping groups present higher plaque index values (p<0.01),^52^ but these findings are inconsistent across studies.^92^ An increased clinical attachment level was observed in ENDS users (0.2 mm, p< 0.5), suggesting greater loss of clinical attachment than in non-smokers.^52^ However, bleeding on probing was lower in ENDS users (13.73% lower, p<0.01).^52 79^ The findings also revealed more MBL in ENDS users (0.19 mm greater, p=0.04).^52^

#### Gingival bleeding

Some studies reported similar rates of gingival pain and bleeding between non-smokers and ENDS users, whereas others reported increased risks (OR 1.76; 95% CI 1.12 to 2.76).^92^

#### Oral symptoms

ENDS users often experience symptoms affecting the lips, tongue and oral tissues, such as dryness, burning, irritation and bad breath, more than non-smokers do but less than traditional tobacco users do.^101^ Ingredients such as nicotine and menthol have been linked to increased oral mucosa blood flow and mouth irritation, respectively.^101^

##### Throat symptoms

Throat dryness, irritation and coughing are commonly reported by ENDS users, with mixed reports on conditions such as tonsillitis.^101^

### Birth outcomes

The evidence on the effects of ENDS on birth outcomes is limited; one systematic review with critically low quality was performed.^45^

In a study from an Irish maternity hospital, the average birth weight of babies from mothers who vaped during their last trimester was 3470±555 g, almost identical to the 3471±504 g of babies from non-smoking, non-vaping mothers. Babies born to smoking mothers averaged a lower 3166±504 g. In a US cohort of 248 pregnant women, babies of dual users had an RR for smallness for gestational age of 2.5 (95% CI 0.7 to 8.8), comparable to the RR of 2.6 (95% CI 0.9 to 7.2) for individuals who use tobacco cigarettes.^45^ Exclusive ENDS users had an RR of 5.1 (95% CI 1.2 to 22.2), but the study emphasised its small sample size as a limitation.

### Breastfeeding

The breastfeeding rates at discharge were 61.1% for non-smoking/non-vaping mothers, 48.6% for ENDS users and 27.2% for individuals who use tobacco cigarettes. For mothers who vaped and smoked (dual users), the outcomes resembled those of individuals who used tobacco cigarettes.^45^

### Mental health

Six systematic reviews were considered, five of which were critically low quality and the rest of which were low quality.^40 50 56 64 66 74^

#### Depression

Adolescents using ENDS are more likely to experience depressive symptoms, with a bidirectional relationship between depression and ENDS use.^50 74^ The Youth Risk Behaviour Survey (YRBS) indicated a greater occurrence of depressive symptoms among adolescent ENDS users (OR 1.37, 95% CI 1.19 to 1.57).^50^ Although ENDS users generally exhibit higher rates of depression and anxiety than non-users do, these rates are lower than those of dual (ENDS and conventional cigarette) users. Some longitudinal studies have revealed a link between ENDS use and increasing depressive symptoms over time, but others have not established a clear predictive relationship.^40^

#### Suicidality

There is a correlation between ENDS use and increased suicidality among adolescents, with studies showing that ENDS users are more likely to consider and attempt suicide than non-users are (OR 1.23, 95% CI 1.03 to 1.47), especially among dual users and female adolescents.^74^ Cases of intentional misuse of ENDS in suicide attempts have been reported, some of which resulted in death due to lethal nicotine doses.^64^

#### Anxiety

The relationship between anxiety and ENDS use is less clear.^40^ Studies involving high school students in Los Angeles reported that exclusive ENDS users did not report higher levels of anxiety than non-users did, although they had higher levels of panic symptoms.^40^

### Sleep issues

Only one study reported sleep issues associated with the use of ENDS. Youths who used only ENDS (8.90 hours) tended to have shorter total sleep times on weekends than non-users did (9.17 hours) (p<0.01).^111^ However, there was no significant association between the frequency of ENDS use and sleep duration on weekdays (7.39 hours for ENDS users and 7.43 hours for non-users) or sleep quality (p=0.15).^68 111^

### Nicotine poisoning

Similar to our findings of various serious adverse effects of END use, research by Hua and Talbot and Tzortzi *et al* collectively highlights the serious risk of nicotine poisoning from ENDS use, which affects both children and adults.^64 94^ Nicotine poisoning from ENDS occurred in both children (accidental) and adults (predominantly males, intentionally). Children present with a range of symptoms, from vomiting to severe respiratory changes, with some requiring intensive care unit admission and intubation and even fatalities. Adult cases involving higher nicotine doses also vary in terms of outcomes, from complete recovery to severe complications and fatalities.

### Explosions and burn injuries

Explosions of ENDS and injuries have been reported in various case reports and case series.^49^ In some instances, explosions occur in the mouth and leg pockets.^49 64^ The cases showed variation in both the location of the ENDS explosions and the types of burn injuries sustained. ENDS explosions most commonly occur while the devices are in users’ pockets, leading to a diverse range of injuries.^87^ The most frequently affected body areas included the thigh, hand, genitals and face. A significant proportion of injuries are oromaxillofacial, including oropharyngeal burns, oral lacerations, tooth avulsion and various fractures.^49^ The most common facial lacerations or burns affect the lips, tongue, nose and other areas.^49^ Projectile injuries predominantly occur in the lower facial third, involving the maxilla, teeth and lips.^93^ One case of intracranial injury-induced fatality has also been reported.^93^

The factors contributing to these incidents often involve contact with metallic objects, such as coins or keys, in pockets, as well as issues related to battery charging or device modifications.^93^ Approximately 30% of the devices were found to have been altered prior to the explosion, primarily involving modifications to the battery. The severity of injuries varies, with 35% of cases reporting second-degree burns and 20% involving a combination of second-degree and third-degree burns.^87^ The average total body surface area affected by burns is 4.9%, with some instances requiring skin grafting and hospital stays ranging from 1 to 31 days.^87^

## Discussion

This umbrella review represents the most recent and comprehensive analysis of the effects of ENDS on human health. For an in-depth understanding of adverse effects, studies not reporting ORs and CIs, which have not been included in previous systematic reviews, were included in the current review.

Studies consistently demonstrate an increased risk of respiratory diseases, including asthma and EVALI (e-cigarrette or vaping type products associated lung injury), associated with ENDS use. Studies have shown adverse cardiovascular effects, including an increased risk of stroke. Acute respiratory and cardiovascular parameters seem to be altered with ENDS use. ENDS also have negative oral and periodontal effects according to the literature. Case reports and case series studies have reported explosion and burn injuries caused by ENDS. A complex relationship exists between ENDS and mental health. The findings suggest that ENDS use among adolescents is associated with various mental health challenges, including depression, suicidality and, to a lesser extent, anxiety and sleep disturbances. The effects of ENDS on genitourinary, gastrointestinal, reproductive and birth-related outcomes are limited. Similarly, evidence of the carcinogenicity of ENDS based on real-world human studies is limited. Since the current umbrella review highlights the potential harm of using ENDS and like products, any promotion of ENDS should clearly present these findings to inform users about the possible risks.

### Vascular dysfunction, platelet function and oxidative stress

Studies have reported that ENDS use increases oxidative stress, resulting in higher levels of reactive oxygen species and lower levels of antioxidants, which are linked to various vascular dysfunctions and affect wound healing, potentially contributing to atherosclerosis.^37 67^ Additionally, ENDS plays a role in thrombosis by influencing platelet activity, increasing the secretion and aggregation of platelet microparticles and affecting soluble P-selectin levels.^67^ The evidence pertaining to the association of ENDS use with myocardial infarction is characterised by inconsistent results. Studies to date have not provided conclusive outcomes, with some suggesting a strong potential link (OR 4.09, 95% CI 1.29 to 12.98) while others finding no significant association (OR 1.65, 95% CI 0.51 to 5.32).^112^,^114^ While the impact on platelet function varies across studies, with some reporting no significant changes and others noting increased platelet adhesion and activation, the cumulative effects on heart rate, blood pressure, endothelial and platelet functions, and increased oxidative stress present potential cardiovascular complications among ENDS users.^37 67^

### EVALI

Our analysis did not reveal significant damage to pulmonary function test values; however, studies on respiratory outcomes associated with ENDS use have revealed significant concerns. ENDS users exhibit evidence of airway inflammation and damage, characterised by increased inflammatory cells and cytokines.^63^ Over 2800 EVALI cases, including 68 deaths, were reported in one review. Radiological findings in EVALI patients commonly include bilateral infiltrates and ground-glass opacities.^64^ Another review identified 58 cases of respiratory issues linked to vaping, with EVALI being the most frequent, alongside other conditions such as organising pneumonia and bronchiolitis obliterans.^115^ Our analysis revealed an increased risk of asthma in ENDS users. Furthermore, case reports have linked ENDS use to severe respiratory conditions, including pneumothorax and exacerbated asthma.^115^ Even after a single use, ENDS can trigger acute respiratory effects such as coughing.^62^

### Novelty in this umbrella review

A previous umbrella review reported findings similar to those of our study.^115^ These findings indicate that the use of nicotine ENDS increases the risk of various negative health effects. These include addiction, poisoning, inhalation toxicity (which can lead to seizures) and lung injury, which are primarily linked to products containing tetrahydrocannabinol or vitamin E acetate. Adverse effects on cardiovascular health, including changes in blood pressure and heart rate, as well as reduced lung function, were also observed in their study. The review highlighted that non-smoking youths who use ENDS are approximately three times more likely than non-smoking non-users to begin smoking tobacco and eventually become individuals who use tobacco cigarettes regularly. They further reported that the impacts of ENDS on the environment include indoor air pollution, waste and fires, which were not part of the study.

However, the current review is more comprehensive in terms of the inclusion of several meta-analyses and systematic reviews that have been conducted since. Our review included recent systematic reviews and presented a wide array of adverse health outcomes of ENDS. The current review also incorporated the psychological effects of ENDS use.

### Nicotine poisoning and device explosions

The use of ENDS is associated with other safety concerns, including nicotine poisoning and device explosions. Nicotine poisoning, affecting both children (often accidental) and adults (frequently intentional), can result in severe symptoms ranging from vomiting to respiratory distress, with some cases requiring intensive care or leading to fatalities. Additionally, ENDS explosions, often triggered by battery malfunctions or contact with metallic objects, have caused diverse injuries, including oromaxillofacial burns, lacerations and fractures, predominantly affecting the face, hands and thighs.

Although the outcomes of this umbrella review do not encompass cessation data, we assert that it is essential to contextualise our findings regarding the physiological effects of ENDS considering its principal application in cigarette smoking cessation. This part uses the extensive literature to furnish essential context for doctors and public health policy-makers, while recognising that the efficacy of ENDS for quitting is a distinct and intricate subject now under examination in a separate manuscript.

### ENDS use in the context of smoking cessation

ENDS, often marketed as less harmful alternatives to traditional cigarettes and as aids for smoking cessation, remain a subject of intense debate among researchers, policy-makers and health organisations. Despite claims, the evidence regarding their effectiveness and safety is mixed.^116 117^ Currently, there are insufficient long-term data to determine whether ENDS are effective for tobacco smoking cessation conclusively. Some RCTs indicate that ENDS may help some tobacco users reduce tobacco use or quit smoking altogether.^118^ However, several observational studies have indicated no significant beneficial effect of ENDS for quitting tobacco.^119^ Moreover, they also pose health risks, including the potential for nicotine addiction among non-smokers, especially youths.^104 120^ The controversy extends beyond clinical benefits to the public health implications of ENDS manufacturers’ marketing practices. These companies often advertise their products as effective tools for quitting tobacco despite a lack of substantial evidence to support such claims.^103 121^ This misleading marketing strategy primarily targets young and non-smoking populations, potentially fostering a new generation that is dependent on nicotine.^122^,^124^ Research emphasises that non-smokers and young people are especially susceptible to the negative effects of ENDS, including an increased risk of starting tobacco smoking, as demonstrated by multiple studies.^115 125 126^ Therefore, these negative aspects of ENDS should be taken into consideration in the regulation and policy-making surrounding these products.

### Regulating ENDS

Regulating ENDS poses a significant challenge for policymakers, particularly regarding their accessibility to young people. Legislation concerning ENDS varies globally, with numerous countries enacting stringent regulations or outright bans. According to the WHO, 34 countries prohibit the sale of ENDS, including Brazil, India, Iran and Thailand; 88 countries lack a minimum age requirement for purchasing these products and 74 countries have no regulations in place.^127^ To deter ENDS from becoming a pathway to tobacco smoking, it is essential to reassess their availability to adolescents.^128^ The WHO urges immediate action to curb ENDS use, especially to safeguard children and tobacco non-users, highlighting the lack of evidence for public health benefits and growing concerns about health risks. Countries with ENDS bans are encouraged to enforce these bans strictly and monitor their impact while allowing them to implement stringent regulations, such as flavour bans and nicotine restrictions.^129^ All nations are advised to promote traditional smoking cessation methods and avoid endorsing ENDS as consumer products for quitting smoking, emphasising the need for controlled access and regulation as medicinal products rather than consumer goods.^129^ Regulators must take steps to monitor ENDS use and capture the use of ENDS among adults and youth through regular and periodic surveillance. Public health campaigns are crucial to informing the public about the dual aspects of ENDS: their use (which is debated) in assisting adults in quitting smoking and the risks they pose, particularly to non-smokers and younger individuals. Implementing policies that require detailed reporting of adverse events, including device malfunctions, is of paramount importance. This multifaceted approach is vital in managing the complex public health issues surrounding ENDS.

There is a pressing need for long-term cohort studies to understand the comprehensive health impacts associated with ENDS use. This includes potential carcinogenic effects and impacts on various body systems. The complex relationship between ENDS use and mental health, particularly among adolescents, needs further exploration. Research should also delve into behavioural patterns that lead to dual use (ENDS and tobacco) and polysubstance use. Policy initiatives should promote and financially support extensive research on the long-term effects of ENDS.

### Strengths and limitations

A significant strength of our umbrella review is its comprehensive coverage of all adverse effects associated with the use of ENDS. Furthermore, we included numerous recently published systematic reviews on the outcomes of interest, ensuring a current and up-to-date analysis. In addition, we delved into individual studies in which ORs and CIs have not been documented in systematic reviews to obtain an in-depth understanding of adverse effects.

However, several limitations of our review should be acknowledged. Our review included only articles published in English. Many of the outcomes of these studies relied on self-reports from users, which can introduce subjective biases. The type of ENDS used was not available in most studies. A significant limitation of this umbrella review is the intentional exclusion of studies exclusively on NNVP. While our analysis focused on the effects of nicotine, it is well established that many health effects of ENDS can be attributed to non-nicotine components, such as flavouring agents, propylene glycol, vegetable glycerin and metal particulates. The decision to exclude NNVP was based on the review’s primary focus on nicotine-related health effects in the context of smoking cessation. As a result, our findings may not fully represent the complete spectrum of health effects from all types of vaping products. Further research is needed to comprehensively characterise the health impacts of NNVP, independent of nicotine. Also, the studies included different confounding factors, which could have added to the significant level of heterogeneity. We know that demographic and clinical characteristics like sex, age and economic status might change the effect, which could explain some of the differences between studies. For instance, e-cigarettes may be a more tempting way to quit smoking for some groups of people, such as younger people or people who do not need nicotine as much. On the other hand, NRTs might be a better choice for some people, like long-term smokers who are very dependent on nicotine. However, we could not officially test for these kinds of interactions or conduct subgroup analyses since we lacked the necessary individual-level data. This is a major limitation in our review and an important area for future studies. We have not considered effect modifications due to a lack of required data. We did not combine the results of observational studies for further meta-analysis due to the significant complexity of the data, arising from differences in PICO elements and methodological heterogeneity among these studies. Most of the risks mentioned are relative risks, as the studies did not report absolute risk separately. For some outcomes, only a limited number of studies are available; therefore, ORs were not mentioned. We were unable to perform a sensitivity analysis due to the vast range of outcomes. A further consideration is the varying number of studies contributing to each meta-analysis, which directly impacts the statistical power of the pooled effect estimate. Table 3 shows that the meta-analyses with the most studies included (eg, heart rate (N=25), SBP (N=23) and DBP (N=23)) had the most statistically significant results. This is a direct result of having more statistical power. By pooling data from multiple studies, the pooled effect estimate becomes more accurate, increasing the likelihood of finding a statistically significant effect, even if the effect size is modest. On the other hand, for outcomes with smaller studies contributing to them, the meta-analysis may not have had enough power to find a minor but clinically important effect. Therefore, the non-significant findings for these outcomes should be interpreted with caution, as they do not necessarily indicate a true absence of an effect. Furthermore, some of the articles in the meta-analysis and independent meta-analysis may have been conducted by authors affiliated with the tobacco industry; however, we were unable to isolate those studies or meta-analyses due to the non-declaration of conflicts of interest.

We would also like to highlight that quantifiable variables, such as those with more fine-grained measures (eg, mean and SD), were not consistently documented in many of the primary studies. This lack of detailed data necessitated our reliance on less statistically powerful dichotomous measures, which may have limited our ability to detect a true effect. This gap in the existing literature implies a significant possibility for further research topics, where the systematic documentation of continuous, quantifiable variables would enable more powerful and exhaustive reviews in the future.

## Conclusions

This umbrella review highlights the significant health risks associated with ENDS use, especially in terms of respiratory and cardiovascular health. The evidence suggests a need for stringent regulation of these products and public health campaigns to educate people about health risks and protect people’s health. Further comprehensive research is crucial to understand the long-term effects of ENDS on various health aspects, particularly in adolescents and youth. Addressing these concerns is imperative for developing effective public health policies and curbing the use of these products.

## Supplementary material

10.1136/bmjopen-2025-100168 online supplemental file 1

## Data Availability

All data analysed in this umbrella review are taken from previously published and publicly accessible systematic reviews and meta-analyses, which are fully cited in the reference list. No additional primary data were obtained. The datasets generated and/or analysed during the current study (ie, the extracted and synthesised data from the included reviews) are not publicly deposited but can be obtained from the corresponding author on reasonable request.
