# Supplementary material for: Health impacts of electronic nicotine delivery systems: an umbrella review of systematic reviews
Source: BMJ Open. 2025 Oct 10;15(10):e100168. doi: 10.1136/bmjopen-2025-100168 (PMC12516984; doi:10.1136/bmjopen-2025-100168)
Supplement: online supplemental file 1 [file bmjopen-15-10-s001.docx]

**Supplementary Materials**

**Table S1.** PRIOR Checklist

| Section Topic | # | Item | Location reported |
| --- | --- | --- | --- |
| TITLE | |  |  |
| Title | 1 | Identify the report as an overview of reviews. | Page no 1 |
| ABSTRACT | |  |  |
| Abstract | 2 | Provide a comprehensive and accurate summary of the purpose, methods, and results of the overview of reviews. | Page 2 |
| INTRODUCTION | |  |  |
| Rationale | 3 | Describe the rationale for conducting the overview of reviews in the context of existing knowledge. | Page 2 and 3 |
| Objectives | 4 | Provide an explicit statement of the objective(s) or question(s) addressed by the overview of reviews. | Page 3 |
| METHODS | |  |  |
| Eligibility criteria | 5a | Specify the inclusion and exclusion criteria for the overview of reviews. If supplemental primary studies were included, this should be stated, with a rationale. | Page 4 |
|  | 5b | Specify the definition of ‘systematic review’ as used in the inclusion criteria for the overview of reviews. | Page 4 and 5 |
| Information sources | 6 | Specify all databases, registers, websites, organizations, reference lists, and other sources searched or consulted to identify systematic reviews and supplemental primary studies (if included).  Specify the date when each source was last searched or consulted. | Page 5, Table S2 |
| Search strategy | 7 | Present the full search strategies for all databases, registers and websites, such that they could be reproduced. Describe any search filters and limits applied. | Table S2 |
| Selection process | 8a | Describe the methods used to decide whether a systematic review or supplemental primary study (if included) met the inclusion criteria of the overview of reviews. | Page 5 |
|  | 8b | Describe how overlap in the populations, interventions, comparators, and/or outcomes of systematic reviews was identified and managed during study selection. | Page 5 |
| Data collection process | 9a | Describe the methods used to collect data from reports. | Page 6 |
|  | 9b | If applicable, describe the methods used to identify and manage primary study overlap at the level of the comparison and outcome during data collection. For each outcome, specify the method used to illustrate and/or quantify the degree of primary study overlap across systematic reviews. | Page 6 |
|  | 9c | If applicable, specify the methods used to manage discrepant data across systematic reviews during data collection. | Page 6, table 2 |
| Data items | 10 | List and define all variables and outcomes for which data were sought. Describe any assumptions made and/or measures taken to identify and clarify missing or unclear information. | Page 6, table 2 |
| Risk of bias assessment | 11a | Describe the methods used to *assess* risk of bias or methodological quality of the included systematic reviews. | Page 6 |
|  | 11b | Describe the methods used to *collect* data on (from the systematic reviews) and/or *assess* the risk of bias of the primary studies included in the systematic reviews. Provide a justification for instances where flawed, incomplete, or missing assessments are identified but not re-assessed. | Page 6 |
|  | 11c | Describe the methods used to *assess* the risk of bias of supplemental primary studies (if included). | NA |
| Synthesis methods | 12a | Describe the methods used to summarize or synthesize results and provide a rationale for the choice(s). | Page 6 and 7 |
|  | 12b | Describe any methods used to explore possible causes of heterogeneity among results. | Page 6 and 7 |
|  | 12c | Describe any sensitivity analyses conducted to assess the robustness of the synthesized results. | NA |
| Reporting bias assessment | 13 | Describe the methods used to *collect* data on (from the systematic reviews) and/or *assess* the risk of bias due to missing results in a summary or synthesis (arising from reporting biases at the levels of the systematic reviews, primary studies, and supplemental primary studies, if included). | Page 6 and 7 |
| Certainty assessment | 14 | Describe the methods used to *collect* data on (from the systematic reviews) and/or *assess* certainty (or confidence) in the body of evidence for an outcome. | Page 6 and 7 |
| RESULTS | |  |  |
| Systematic review and supplemental primary study selection | 15a | Describe the results of the search and selection process, including the number of records screened, assessed for eligibility, and included in the overview of reviews, ideally with a flow diagram. | Page 7 and figure 1 |
|  | 15b | Provide a list of studies that might appear to meet the inclusion criteria, but were excluded, with the main reason for exclusion. | NA |

| Section Topic | # | Item | Location reported |
| --- | --- | --- | --- |
| Characteristics of systematic reviews and supplemental primary studies | 16 | Cite each included systematic review and supplemental primary study (if included) and present its characteristics. | Table 2 |
| Primary study overlap | 17 | Describe the extent of primary study overlap across the included systematic reviews. | NA |
| Risk of bias in systematic reviews, primary studies, and supplemental primary studies | 18a | Present assessments of risk of bias or methodological quality for each included systematic review. | Table S4 |
|  | 18b | Present assessments (*collected* from systematic reviews or *assessed* anew) of the risk of bias of the primary studies included in the systematic reviews. | Table 2 |
|  | 18c | Present assessments of the risk of bias of supplemental primary studies (if included). | Table 2 |
| Summary or synthesis of results | 19a | For all outcomes, summarize the evidence from the systematic reviews and supplemental primary studies (if included). If meta-analyses were done, present for each the summary estimate and its precision and measures of statistical heterogeneity. If comparing groups, describe the direction of the effect. | Page 7 to 15 |
|  | 19b | If meta-analyses were done, present results of all investigations of possible causes of heterogeneity. | Table 3 |
|  | 19c | If meta-analyses were done, present results of all sensitivity analyses conducted to assess the robustness of synthesized results. | NA |
| Reporting biases | 20 | Present assessments (*collected* from systematic reviews and/or *assessed* anew) of the risk of bias due to missing primary studies, analyses, or results in a summary or synthesis (arising from reporting biases at the levels of the systematic reviews, primary studies, and supplemental primary studies, if included) for each summary or synthesis assessed. | Supplementary file |
| Certainty of evidence | 21 | Present assessments (*collected* or *assessed* anew) of certainty (or confidence) in the body of evidence for each outcome. | page 7-15 |
| DISCUSSION | | |  |
| Discussion | 22a | Summarize the main findings, including any discrepancies in findings across the included systematic reviews and supplemental primary studies (if included). | Page 15 |
|  | 22b | Provide a general interpretation of the results in the context of other evidence. | Page 15-16 |
|  | 22c | Discuss any limitations of the evidence from systematic reviews, their primary studies, and supplemental primary studies (if included) included in the overview of reviews. Discuss any limitations of the overview of reviews methods used. | Page 18 |
|  | 22d | Discuss implications for practice, policy, and future research (both systematic reviews and primary research). Consider the relevance of the findings to the end users of the overview of reviews, e.g., healthcare providers, policymakers, patients, among others. | Page 18 |
| OTHER INFORMATION | | |  |
| Registration and protocol | 23a | Provide registration information for the overview of reviews, including register name and registration number, or state that the overview of reviews was not registered. | Page 4 |
|  | 23b | Indicate where the overview of reviews protocol can be accessed, or state that a protocol was not prepared. | Page 4 |
|  | 23c | Describe and explain any amendments to information provided at registration or in the protocol. Indicate the stage of the overview of reviews at which amendments were made. | Page 4 |
| Support | 24 | Describe sources of financial or non-financial support for the overview of reviews, and the role of the funders or sponsors in the overview of reviews. | Page 19 |
| Competing  interests | 25 | Declare any competing interests of the overview of reviews' authors. | Page 19 |
| Author information | 26a | Provide contact information for the corresponding author. | Page 1 |
|  | 26b | Describe the contributions of individual authors and identify the guarantor of the overview of reviews. | Page 19 |
| Availability of data and other materials | 27 | Report which of the following are available, where they can be found, and under which conditions they may be accessed: template data collection forms; data collected from included systematic reviews and supplemental primary studies; analytic code; any other materials used in the overview of reviews. | Page 19, supplementary material |

**Table S2:** Search strategy

| **Search** | **Query** | **Results** |
| --- | --- | --- |
| #4 | Search: **((((("Electronic Nicotine Delivery Systems"[Mesh]) OR ("Vaping"[Mesh])) OR (("e-cig*" OR "ecig*" OR "e cig*" OR "electronic cig*" OR "electronic nicotine*" OR vape OR vapes OR vaporizer OR vapourizer OR vaporiser OR vapouriser OR vaper OR vapers OR vaping OR e-liquid OR ENDS))) OR ((E Cigarettes) OR (E-Cigarette) OR (E Cigarette) OR (Electronic Cigarette) OR (Cigarette, Electronic) OR (Cigarettes, Electronic))) AND (((((("Adolescent"[Mesh]) OR ("Adult"[Mesh] OR "Young Adult"[Mesh])) OR (young people)) OR (middle aged)) OR (older adult)) OR (older people))) AND ((((systematic review) OR (systematic reviews)) OR (meta analysis)) OR (network meta analysis))** Sort by: **Most Recent** | 1,564 |
| #3 | Search: **(((systematic review) OR (systematic reviews)) OR (meta analysis)) OR (network meta analysis)** Sort by: **Most Recent** | 468,238 |
| #2 | Search: **((((("Adolescent"[Mesh]) OR ("Adult"[Mesh] OR "Young Adult"[Mesh])) OR (young people)) OR (middle aged)) OR (older adult)) OR (older people)** Sort by: **Most Recent** | 9,046,337 |
| #1 | Search: **((("Electronic Nicotine Delivery Systems"[Mesh]) OR ("Vaping"[Mesh])) OR (("e-cig*" OR "ecig*" OR "e cig*" OR "electronic cig*" OR "electronic nicotine*" OR vape OR vapes OR vaporizer OR vapourizer OR vaporiser OR vapouriser OR vaper OR vapers OR vaping OR e-liquid OR ENDS))) OR ((E Cigarettes) OR (E-Cigarette) OR (E Cigarette) OR (Electronic Cigarette) OR (Cigarette, Electronic) OR (Cigarettes, Electronic))** Sort by: **Most Recent** | 367,626 |

**Table S3:** Characteristics of the included systematic reviews

| **Study** | **Type of included studies** | **Countries of included studies** | **Year of studies** | **Number of Studies** | **Population** | **Sample size** | **Risk of Bias** | **Outcomes Assessed** | **Funding and conflict of interest** |
| --- | --- | --- | --- | --- | --- | --- | --- | --- | --- |
| Anandan 2023 (36) | RCTS and cross-sectional studies | USA, Malaysia, Canada, KSA, Poland, Hungary, Italy, New Zealand, UK | NA | 18 | Healthy current or former smokers using nicotine E-cigarette (dual users excluded) | 3756 | Good quality on NOS, some concerns and low risk of bias for RoB-2 | Adverse effects | None |
| Ashour 2023 (37) | NA | NA | 2016-2022 | 37 (18 human studies) | E-cigarette users | 5311 (human studies) | NA | Surgical wound healing (Inflammation and oxidative stress markers, Blood flow, Platelet function) | None |
| Awad 2023 (38) | Cohort, Cross-sectional | USA | 2021-2023 | 6 | E-cigarette users | 1024401 | High quality | Risk of stroke | None |
| Bandara 2023 (39) | Cross-sectional,  experimental, case series, and RCTs | NA | 2015-2023 | 28 | E-cigarette users | NA | Moderate to high quality | Genitourinary impacts | None |
| Becker 2020 (40) | Cross-sectional and Cohort studies | USA, UK, South Korea, Taiwan | 2015-2020 | 40 | Adolescents and young adults | NA | Weak to moderate quality | Mental health | None |
| Bjurlin 2020 (41) | RCTs, Data analysis from databases | NA | 2015-2020 | 22 | E-cigarette users | 1259 | NA | Presence of carcinogens in urine | None |
| Bourke 2021 (42) | Cross-sectional studies | USA, Canada & Hong Kong | 2016-2020 | 7 | Electronic cigarette use in children and adolescents (up to 20 years) | 52392 | NA | Coughing | None |
| Bozier 2020 (43) | Case reports, Survey, Meta Analysis and RCTs. Anything reported have been included | NA | 2017-2019 | 225 | E-cigarette users | NA | NA | Wide range of outcomes like harm reduction, lung and respiratory, cardio effects | None |
| Bravo-Gutiérrez 2021 (44) | NA | NA | NA | NA | E-cigarette users | NA | NA | Lung Damage | Funding: Instituto Nacional de Enfermedades Respiratorias Ismael Cosío Villegas (INER).    Conflict of interest: None |
| Calder 2021 (45) | Cross sectional, Surveys, Cohort, Qualitative interview or focus group  data and RCTs | USA, UK, Ireland, Multicounty | 2015-2019 | 23 (3 studies for health outcomes, 6 for smoking cessation) | Pregnant E-cigarette users | NA | Fair to good (for health outcomes and smoking cessation) | Birth outcomes, smoking cessation | Funding: A grant from Public Health England and  National Institute for Health Research (NIHR) Applied Research Collaboration South London (NIHR ARC South London) at King’s College Hospital NHS Foundation Trust Conflict of interest: None |
| Chand 2022 (46) | Cross-sectional, cohort studies | USA, Canada, Korea, Kuwait | 2016-2020 | 13 | E-cigarette users | 1039203 | Fair to good quality | Asthma | None |
| D Ambrosia 2022 (47) | Case‒control, Cohort and Cross-sectional studies | NA | 2015-2019 | 18 | Smokers/E cigarette users | NA | Bias due to confounding: Critical,  Bias in selection of participants: Serious,  Bias in measurement of outcomes: Moderate.  All other biases as per the ROBINS-1 tool | Clinical, radiographic, and inflammatory periodontal and peri-implant  tissue parameters | None |
| Damay 2022 (48) | RCTs and Observational studies | NA | NA | 10 (Human) | Smokers, nonsmokers, Dual users | 77055 | Some had high, some had low risk | Vascular outcomes | None |
| Dekhou 2021 (49) | Case report, Case series | NA | 2016-2019 | 20 | Male participants | 21 | NA | Electronic Cigarette-Related Oromaxillofacial Injuries | None |
| Farooqui 2023 (50) | Longitudinal and cross-sectional studies | Canada, Hong Kong, Australia, USA, Sweden Korea, New Zealand, Isfahan | NA | 122 (total for tobacco cigarettes and E-cigarettes) | Adolescents | 72438 (for E-cigarette studies) | NA | Depression, mental and behavioral outcomes | None |
| Farsalinos 2014 (51) | Clinical, animal toxicological studies | NA | NA | NA | NA | NA | NA | Health outcomes, passive effect and smoking cessation with E-cigarettes | None |
| Figueredo 2020 (52) | Cross-sectional design | KSA | 2017-2020 | 8 | Individuals above age 13 (25-45 age in included studies) | 582 | Moderate to high quality | Periodontal effects | None |
| Flach 2019 (53) | 13 Laboratory based investigations, 05 clinical research (2 cohorts, 2 case‒control and 1 case series) | USA, South Korea, Germany, Turkey, Canada, Italy, Vietnam | 2014-2018 | 18 | General population samples of adolescents and adults, any age | 224 (of 5 clinical studies) | High risk of bias | Head and neck Cancer Pathogenesis | None |
| Garcia 2020 (54) | NA | USA | NA | 19 | Users of earlier-generation EC devices | NA | NA | Autonomic cardiovascular effects | Funding: The Tobacco-Related Disease Research Program (TRDRP)    Conflict of interest: None |
| Gentry 2019 (55) | 9 studies: 5 quantitative (secondary analysis, uncontrolled before and after, cohort) and 4 qualitative (FGD and online postings) | USA, Australia, Italy, New Zealand, one study was conducted globally (online) | 2004-2017 | 9 | Vulnerable participants of any age or country with mental illness, substance misuse, homelessness, and involved in the criminal justice system | 1217 | High risk of bias | Adverse events | None |
| Glasser 2017 (56) | Human and animal, cell studies | NA | NA | 676 (total animal, cell, and human) | General population | NA | NA | Physiologic, cognitive, adverse effects, and second hand exposure effects | Funding: The Schroeder Institute at Truth Initiative, the Robert Wood Johnson Foundation and a NIH K01 Career Development Award in Tobacco Control Regulatory Research    Conflict of interest: None |
| Goniewicz 2020 (57) | Cross-sectional and longitudinal | NA | NA | 6 | E-cigarette users and smokers | Na | Moderate quality | Respiratory and cardiovascular outcomes | Funding: Research grant from Pfizer and personal fees from Johnson and Johnson, outside of this work    Conflict of interest: None |
| Gonsalves 2021 (58) | Case reports, case series, retrospective studies | NA | NA | 23 | Children/Paediatrics (13-18 years) | NA | NA | EVALI | None |
| Gualano 2014 (59) | Experimental and cohort studies | USA, Italy, New Zealand, Greece and UK | 2010-2013 | 12 | Smokers | 1293 (Cohort studies) | Poor quality | Adverse effects, Physiological effects | None |
| Guo 2022 (60) | Case‒control, cross-sectional | India, Pakistan, USA, Poland, Italy, KSA, China, Egypt, Croatia, Iraq, Jordan, Kuwait, Lebanon, Malaysia, Nigeria, South Africa, Turkey and Yemen | 2000-2022 | 14 | Healthy smokers | 6827 | Moderate-High quality | Xerostomia | Funding: Shanghai Association for Science and Technology and innovation research team of high-level local universities in Shanghai.    Conflict of interest: None |
| Hartmann-Boyce 2022 (61) | RCTs and crossover trials | USA, UK, Italy, Australia, New zealand, Canada, Belgium, Ireland, Poland, the Republic of Korea, South Africa,  Switzerland, and Turkey | 2011-2022 | 78 | Smokers | 22052 | Ten studies  to be at low risk of bias, 18 to  be at unclear risk, and the remaining 50 at high risk of bias | Adverse events, respiratory and cardiac parameters | Conflict of Interest:  Authors were PI of some included studies.    Funding: Grants from pharmaceutical companies and other institutions |
| Hess et al 2016 (62) | 3 experimental, 1 observational, 1 animal study and 11 laboratory investigations | Not mentioned | 2012-2015 | 16 | NA | 79 (of 04 human studies) | High risk of bias | Health effects from passive exposure to EC vapour | None |
| Honeycutt 2022 (63) | RCTs, nonrandomized studies, cohort studies | Greece, Italy, Canada, USA | 2013-2021 | 8 | E cigarette users | 273 | Low to moderate quality | Lung functions | None |
| Hua 2016 (64) | Case reports | NA | 2012-2016 | 26 | E cigarette users | NA | NA | Cardiovascular, respiratory, gastrointestinal, neurological, mechanical injury, nicotine poisoning, misuse, and suicide attempts. | None |
| Ioakeimidis 2016 (65) | NA | NA | NA | 20 | E cigarette users | NA | NA | Respiratory and cardiovascular effects | None |
| Keijsers 2022 (66) | Mixed pre-posttest design,  Within-participant experimental design,  Pre-posttest design with no control group,  Regression analysis | NA | NA | 22 | Smokers and dual users | NA | Overall, the quality of the studies was high | Cue reactivity | Funding: A  Health Research Council (HRC) of New Zealand Explorer Grant    Conflict of interest: None |
| Kennedy 2019 (67) | Human and nonhuman experimental studies | NA | NA | 24 (Human) | Smokers and nonsmokers | NA | 1/3 of the studies had a moderate to high risk of bias | Heart functions and related outcomes | None |
| Kwon 2019 (68) | Observational studies | NA | NA | 1 (for E-cigarettes) | Adolescents (10-22 years) | NA | Self-reporting bias | Sleep problems | Conflict of interest: None |
| La Rosa 2023 (69) | RCT, cohort, Quasi experiment | USA, UK, Italy, Germany, Belgium, Greece, Indonesia, Poland, and South Africa | NA | 26 | Adults who smoke cigarettes | 1742 | Mostly high bias | Cardiac parameters | None |
| Larue 2021 (70) | Crossover studies and randomized parallel-group studies | NA | 2010-2021 | 27 | Adults either previously smokers or nonsmokers | 919 | Low risk of bias | Cardiovascular, respiratory effects | Conflict of Interest: Authors were associated with pharma companies, but declared the work is not associated with it |
| Li 2022 (71) | Cross-sectional | USA, South Korea, Canada | 2016-2020 | 10 | Young adults | 483948 | Moderate quality | Asthma | Funding: The Research Center of Traditional Chinese Medicine (TCM), Gansu Province, TCM evidence-based Capacity Building project, and Clinical practice of TCM comprehensive prevention and treatment of heart failure based on evidence-based research.    Conflict of interest: Not reported |
| Liber 2023 (72) | RCTs, longitudinal and cross-sectional studies | USA, UK, Australia, KSA, Netherlands, Spain, Greece, New zealand, Canada | 2013-2020 | 29 | E-cigarette users | NA | Mostly unclear risk of bias | Quit intent, quit success | Funding: NIH. Funding from imvaria, Inc. for unrelated work.  Conflict of interest: None |
| Liu 2018 (73) | Observational studies, surveys, RCTs | NA | NA | 14 | Smokers | NA | Moderate quality | Adverse effects | None |
| Livingston 2022 (74) | Cross-sectional, longitudinal | USA, South Korea, Canada | 2016-2019 | 18 | Adolescents (13-24 years old) | NA | Overall high quality | Oral health, respiratory issues, depression, suicidality, nicotine dependence, anxiety | None |
| Martinez-Morata 2020 (10) | 13 e-cig trials (12 crossover designs) and 1 observational study | USA, Greece, Italy, Poland, Belgium, Germany, Sweden, UK | 2012-2020 | 14 | Adult e-cigarettes users (20-58 years old) | 318 | Low risk of bias | Blood pressure endpoints (SBP and DBP) | None |
| Meng 2023 (75) | RCTs | NA | 2016-2020 | 8 | Healthy smokers and nonsmokers | 712 | Low risk of bias | Vascular endothelial function | Funding: National Natural Science Foundation of China    Conflict of interest: None |
| Notley 2021 (76) | Cross-sectional survey, longitudinal cohort, and qualitative studies | USA, UK, Korea, Taiwan, Mexico | 2015-2020 | 58 | Young (<18 years) e-cig smokers | 512874 | Low risk of bias | Adverse effects | None |
| Oloyede 2021 (77) | RCTs, Meta-analysis, Systematic review, Cross-sectional survey, Longitudinal survey | Canada, USA, England, Australia, New Zealand | 2006-2019 | 18 | Tobacco smokers of any age or country | 118650 | Low risk of bias | Adverse effects | None |
| Patel et al 2020 (78) | RCTs, Prospective Cohort | Not mentioned | Not mentioned | 42 | Cigarette and E-cigarette smokers (any age or country) | Not mentioned | High risk of bis | Impact of cigarettes on middle-ear disease, otological surgery and sensorineural hearing loss | None |
| Pesce et al 2022 (79) | Clinical observational and cross-sectional trials | Saudi Arabia | 2017-2020 | 5 | Cigarette smokers, water-pipe smokers, E-cigarette smokers and nonsmokers | 512 | Low risk of bias | Periodontal probing depth (PD), plaque index (PI), and bleeding on probing (BOP) | None |
| Pisinger et al 2014 (80) | 34 studies investigating content/effect of e-fluid or –vapour, 20 studies reporting adverse events, 21 human experimental studies, and one animal experimental study | Not mentioned | All studies upto 2014 | 76 | E-cigarette users, dual users, nonusers (any age or country) | 677 | High risk of bias | Any health-related event or symptom | None |
| Prasetyo et al 2020 (81) | Cross sectional, prospective cohort study | Turkey, India, Brazil, Italy | 2011-2018 | 16 | Smokers, nonsmokers, and ex-smokers (any age or country) | 1362 | Low risk of bias | Changes in nasal mucociliary clearance (NMC) | None |
| Qureshi et al 2023 (82) | RCTs, Quasi, and Cohort | Greece (5), United King- dom (4), USA (3), Italy (2), and one each from Belgium and Hungary | 2013-2020 | 16 | E-cigarette and conventional cigarette users aged 18-73 years  Six studies included participants with asthma or COPD | 1357 | Moderate to high risk | Lung Function Tests | Funding: ECLAT srl, a research-based spin-off company of the University of Catania, with the help of a grant from the Foundation for a Smoke-Free World    Conflict of interest: None |
| Rahman 2023 (83) | Observational and trials | NA | 2017-2022 | 15 | Current E-cigarette smokers, current tobacco cigarette smokers, dual  cigarettes smokers, and former tobacco cigarette smokers | 85420 | Good quality | Myocardial infarction, SBP, DBP, MBP, HR | None |
| Ralho et al 2019 (84) | Case‒control, clinical observational study | Saudi Arabia | 2016-2018 | 08 | E-cigarette smokers, conventional cigarette smokers, water-pipe smokers, nonsmokers | 1283 | Low risk of bias | Oral cavity problems: BOP, bleeding on probing; CAL, clinical attachment loss; IL-1B, interleukin 1 beta; MBL, marginal bone loss; MMP-9, matrix metalloproteinase-9; MT, missing teeth; OML, oral mucosa lesions; PBI, papillary bleeding index; PI, plaque index; PD, probing depth; PIBL, peri-implant bone loss; RBL, radiographic bone level; TNF-a, tumor necrosis factor-a | None |
| Riley et al 2015 (85) | RCT, Case series, Prospective cohort, Cross-sectional study | USA, Greece, Poland, Italy, South Africa | 1997-2014 | 13 | Cigarette and E-cig smokers, nonsmokers and ex-smokers | 20794 | Low risk of bias | Changes in BP, Heart Rate, any adverse events | None |
| Scarpino 2020 (86) | Case-report | USA Korea, Italy, Germany, Poland, Denmark, Canada, China, France, Japan, Netherlands, Switzerland and Turkey | 2010-2020 | 38 | General population | NA | NA | Nicotine intoxication by e-cigarette liquids | None |
| Seitz 2018 (87) | Case series, case reports | USA, UK, Canada, Germany, and France | 2015 to 2017 | 31 | E-cigarette users | 156 | NA | Burn injuries caused by E-cigarettes | None |
| Sharma 2022 (88) | Cross-sectional | USA, Greece | 2018, 2019 | 4 | Current e-cigarette users, traditional cigarette users, and non-e-cigarette users | 585306 | Low risk of bias | Myocardial infarction | Conflict of interest: None |
| Siddiqi 2023 (89) | RCTs | UK, USA, Sweden, Greece, Belgium, Poland, Indonesia, Italy, Canada | 2010-2022 | 27 | E-cigarette users | NA | NA | Heart rate, SBP, DBP, MAP | Funding: From pharmaceutical companies    Conflict of interest: None |
| Skotsimara 2019 (17) | NA | NA | NA | 26 (clinal and nonclinical) | Smokers and e-cigarette users | NA | Confounding bias detected in many studies | Cardiac parameters | None |
| Sreedharan 2021 (90) | Case series, case report and prospective cohort study | NA | 2016-2020 | 42 | Vapers (Nicotine, marijuana, THC) | 184 | 19 had a low risk of bias  and 11 studies had a moderate risk of bias. | Lung injury | None |
| Taylor 2023 (91) | RCTS and interventional longitudinal studies | NA | 2017-2022 | 22 | Smokers, E cigarette users and nonusers | NA | RCTs had some concerns, rest of the studies were good quality | NNAL level | Funding: NIHR HPRU, UKHSA, Imperial College, ARC South London, KCH NHS Foundation Trust, SSA, OHID, UKPRP (BHF, CRUK, CSO, EPSRC, ESRC, HSC R&D Division, MRC, NIHR, NERC, PHA NI, Health Foundation, Wellcome). |
| Theim 2023 (92) | Observational studies | NA | NA | 16 | E-cigarette users, smokers, and nonsmokers | NA | Low to moderate quality | Periodontal health | None |
| Tran 2023 (93) | Case series and case reports | NA | 2016 to 2022 | 28 | E-cigarette users mostly male | 32 | Mostly good quality | Oral and Maxillofacial Injuries with E cigarette explosion | None |
| Tzortzi 2020 (94) | Case reports and case series | USA, UK, Australia, Canada, China,  Denmark, France, Germany, Ireland, Italy, Japan, Korea, Malaysia, the Netherlands, Poland, Portugal,  Scotland, South Korea, Switzerland, and Turkey | 2013-2020 | 133 | E-cigarettes users | 238 | NA | Respiratory effects, traumatic injuries, poisoning, allergy, Ulcerative colititis | None |
| Vanderkam 2022 (95) | RCTs | Canada, UK, Korea, Italy, New Zealand | 2013-2021 | 7 | Smokers | 3097 | Low bias | Adverse effects | None |
| Vyncke 2020 (96) | Case series, case reports | USA, UK, Canada,  Germany, France, Belgium, and Malaysia | 2016-2020 | 41 | E-cigarette users | NA | NA | Burns, injuries | None |
| Wasfi 2020 (97) | Cross sectional and cohort, case control, RCT, quasi experimental studies | USA, Middle east, east Asia, Europe | NA | 73 | E-cigarette users | NA | High bias | Health outcomes | None |
| Wills 2021 (98) | Cross-sectional and longitudinal | NA | NA | 24 | Adolescents and adults | Asthma: 971278; COPD: 1023494 | NA | Asthma, COPD | Funding: Grants from National Cancer Institute    Conflict of interest: None |
| Wilson 2022 (99) | In vitro and trials | USA, Canada, Switzerland, Germany, Italy, Poland, Brazil | NA | 18 (2 trials) | E-cigarette users | NA | NA | Effect of E cigarette on head, neck and oral cells | None |
| Xian 2020 (100) | Cross-sectional | USA, Korea | 2016-2020 | 11 | E-cigarette users | 1143118 | Moderate to high quality | Asthma | None |
| Yang 2020 (101) | RCTs, quasiexperimental studies, correlational or descriptive studies, case reports, in vitro  studies | Mainly Europe and north America | 2010-2019 | 99 | E-cigarette users | NA | NA | Oral effects | None |
| Zhao 2022 (102) | Cross-sectional | NA | NA | 6 | E-cigarette users | 1134896 | Low risk of bias overall | Stroke | Funding: Project of Science and Technology Department of Qinghai Province and Mingfei Yang from Mingfei Yang-2020 Kunlun Talents of Qinghai Province High-End Innovation and Entrepreneurship Talent Project-Cultivate Leading Talents.    Conflict of interest: None |

Abbreviations: BNP - B-type Natriuretic Peptide; BMMC - Bone Marrow Mononuclear Cells; BMSC - Bone Marrow-Derived Stem Cells; CAL - Clinical Attachment Loss; cTnT - Cardiac Troponin T; CTRL - Control Group; DBP - Diastolic Blood Pressure; EC - Electronic Cigarette; ENDS - Electronic Nicotine Delivery Systems; EVALI - E-cigarette or Vaping Product Use-Associated Lung Injury; HR - Heart Rate; IL-1B - Interleukin 1 Beta; LVEF - Left Ventricular Ejection Fraction; LVEDd - Left Ventricular End-Diastolic Diameter; MACE - Major Adverse Cardiovascular Events; MBL - Marginal Bone Loss; MBP - Mean Blood Pressure; MMP-9 - Matrix Metalloproteinase-9; MT - Missing Teeth; NA - Not Applicable; NMC - Nasal Mucociliary Clearance; NNAL - 4-(Methylnitrosamino)-1-(3-pyridyl)-1-butanol; NOS - Newcastle‒Ottawa Scale; NYHA - New York Heart Association; OML - Oral Mucosa Lesions; PBI - Papillary Bleeding Index; PD - Period

**Table S4: Excluded studies**

| Vaping-Related Acute Parenchymal Lung Injury: A Systematic Review. | https://dx.doi.org/10.1016/j.chest.2020.03.085 | Not proper SLR |
| --- | --- | --- |
| Efficacy and safety of electronic cigarettes as a smoking cessation intervention: A systematic review and network meta-analysis. | https://dx.doi.org/10.18332/tpc/143077 | NMA |
| How do periodontal indices compare among non-smokers, tobacco and e-cigarette smokers?. | | NMA |
| Treatment of Adolescent e-Cigarette Use: Limitations of Existing Nicotine Use Disorder Treatment and Future Directions for e-Cigarette Use Cessation | 10.1016/j.jaac.2020.07.007 | Not systematic review |
| Disparities in E-Cigarette Harm and Addiction Perceptions Among Adolescents in the United States: a Systematic Review of the Literature | 10.1007/s40615-023-01553-1 | Wrong exposure |
| The efficacy of smoking cessation interventions in low- and middle-income countries: a systematic review and meta-analysis. | 10.1111/add.14518 | Wrong exposure |
| Systematic review of biomarker findings from clinical studies of electronic cigarettes and heated tobacco products. | https://dx.doi.org/10.1016/j.toxrep.2021.01.014 | Wrong outcome |
| Cardiovascular effects of waterpipe smoking: a systematic review and meta-analysis. | 10.31083/j.rcm.2020.03.135 | Wrong exposure |
| The Effect of Tobacco Smoking on Musculoskeletal Health: A Systematic Review. | https://dx.doi.org/10.1155/2018/4184190 | Wrong exposure |
| Association between electronic cigarette use and asth ma symptoms among adolescents: A systematic review | 10.1186/s12919-021-00209-4 | Conference abstract |
| The Association Between E-cigarette Use and Respiratory Symptoms in the Adult Population: A Systematic Review | 10.1164/ajrccm-conference.2022.205.1_MeetingAbstracts.A1347 | Conference abstract |
| Association between Harmful and Addictive Perceptions of E-Cigarettes and E-Cigarette Use among Adolescents and Youthâ€”A Systematic Review and Meta-Analysis. | 10.3390/children9111678 | Wrong outcome |
| [Effects of electronic cigarettes on health: a systematic review of the available evidence.]. | https://dx.doi.org/10.1701/3294.32651 | Not available in english |
| Social Influence in the Uptake and Use of Electronic Cigarettes: A Systematic Review. | 10.1016/j.amepre.2019.08.023 | Wrong outcome |
| A systematic review of experimental and longitudinal studies on e-cigarette use cessation. | https://dx.doi.org/10.1016/j.addbeh.2023.107787 | Wrong outcome |
| Correction: electronic nicotine delivery systems and/or electronic non-nicotine delivery systems for tobacco smoking cessation or reduction: a systematic review and meta-analysis. | https://dx.doi.org/10.1136/bmjopen-2016-012680corr1 | Correction |
| P1-50: Radiological findings of EVALI: A systematic review. | https://dx.doi.org/10.1111/resp.14150_50 | Abstract only |
| Cigarette Smoking and Human Gut Microbiota in Healthy Adults: A Systematic Review. | https://dx.doi.org/10.3390/biomedicines10020510 | Wrong outcome |
| A systematic review and meta-analysis of the cardiovascular effects of E-cigarette | 10.1093/eurheartj/ehz745.0797 | Abstract only |
| Genotoxic and Carcinogenic Potential of Compounds Associated with Electronic Cigarettes: A Systematic Review. | https://dx.doi.org/10.1155/2019/1386710 | Wrong exposure |
| The Orthopedic Effects of Electronic Cigarettes: A Systematic Review and Pediatric Case Series. | 10.3390/children9010062 | Non human studies |
| Does using e-cigarettes increase cigarette smoking in adolescents? | | Abstract only |
| A Systematic Review of the Literature Examining the Effects of Cigarette Smoke and e-Cigarette Vapor on the Virulence of Human Pathogenic Bacteria. | https://dx.doi.org/10.3390/ijerph191912518 | Wrong outcome |
| Impact of COVID-19 lockdown on smoking and vaping: systematic review and meta-analysis. | 10.1016/j.puhe.2023.02.007 | Wrong outcome |
| Smoking cessation attempts among adolescent smokers: a systematic review of prevalence studies. | 10.1136/tc.2006.018853 | Wrong outcome |
| Do e-cigarettes have a part to play in peri-implant diseases?. | https://dx.doi.org/10.1038/s41432-023-00864-w | Commentary |
| SMOKING CESSATION AND E-CIGARETTES | 10.1177/00048674221088686 | Conference abstract |
| [Risk of initiation to smoking with the use of electronic cigarettes: systematic review and meta-analysis]. | https://dx.doi.org/10.1590/1413-812320212612.35032020 | Not available in english |
| 42.9 ELECTRONIC CIGARETTE USE (VAPING) AND MENTAL HEALTH COMORBIDITY: A SYSTEMATIC REVIEW OF STUDIES AMONG ADOLESCENTS | 10.1016/j.jaac.2020.08.328 | Conference abstract |
| Gender Differences in Reasons for Using Electronic Cigarettes: A Systematic Review. | 10.1093/ntr/ntac108 | Wrong outcome |
| Heat-not-burn tobacco products and cardiovascular risk reduction: A systematic review of randomized controlled trials | 10.3233/THC-220677 | Wrong exposure |
| Carcinogen biomarkers in the urine of electronic-cigarette users and implications for potential bladder cancer: A systematic review | | Wrong outcome |
| E-cigarettes and Urologic Health: A Collaborative Review of Toxicology, Epidemiology, and Potential Risks. | https://dx.doi.org/10.1016/j.eururo.2016.12.022 | Not systematic review |
| Tobacco harm reduction: are smokers becoming more hardcore?. | https://dx.doi.org/10.1057/s41271-020-00226-1 | Wrong exposure |
| Longer-term use of electronic cigarettes when provided as a stop smoking aid: Systematic review with meta-analyses. | https://dx.doi.org/10.1016/j.ypmed.2022.107182 | Wrong outcome |
| Pharmacological interventions for smoking cessation: an overview and network meta-analysis. | 10.1002/14651858.CD009329.pub2 | Wrong exposure |
| A systematic review of nicotine by inhalation: is there a role for the inhaled route?. | | Wrong outcome |
| The use of electronic nicotine delivery systems during pregnancy and the reproductive outcomes: A systematic review of the literature. | 10.18332/tid/104724 | Animal studies |
| Acute eosinophilic pneumonia associated with non-cigarette smoking products: a systematic review. | https://dx.doi.org/10.5603/ARM.2020.0088 | Outcomes not differentiated for E cigerattes |
| E-cigarette use and adverse respiratory symptoms among adolescents and Young adults in the United States. | 10.1016/j.ypmed.2021.106766 | Primary study |
| A systematic review of randomized controlled trials and network meta-analysis of e-cigarettes for smoking cessation. | https://dx.doi.org/10.1016/j.addbeh.2021.106912 | NMA |
| Electronic nicotine delivery systems use and asthma in adolescents: A systematic review and meta-analysis | 10.1111/acem.13961 | Abstract only |
| Effect of smoking on COVID-19 severity: A systematic review and meta-analysis | 10.1186/s40635-020-00354-8 | Wrong exposure |
| Is vaping a gateway to smoking: A review of the longitudinal studies | 10.1515/ijamh-2016-0033 | Not systematic review |
| Smoking cessation interventions for adults aged 50 or older: A systematic review and meta-analysis. | 10.1016/j.drugalcdep.2015.06.004 | Wrong exposure |
| A Comparative Health Risk Assessment of Electronic Cigarettes and Conventional Cigarettes. | https://dx.doi.org/10.3390/ijerph14040382 | Wrong outcome |
| Effectiveness and cost-effectiveness of computer and other electronic aids for smoking cessation: a systematic review and network meta-analysis. | 10.3310/hta16380 | Wrong exposure |
| The global impact of tobacco control policies on smokeless tobacco use: a systematic review. | https://dx.doi.org/10.1016/S2214-109X(23)00205-X | Wrong outcome |
| Systematic review of changed smoking behaviour, smoking cessation and psychological states of smokers according to cigarette type during the COVID-19 pandemic. | https://dx.doi.org/10.1136/bmjopen-2021-055179 | Wrong exposure |
| Efficacy and safety of nicotine replacement therapy for smoking cessation in pregnancy: systematic review and meta-analysis. | 10.1111/j.1360-0443.2010.03179.x | Wrong exposure |
| Pharmacological interventions for promoting smoking cessation during pregnancy. | https://dx.doi.org/10.1002/14651858.CD010078.pub2 | Wrong exposure |
| Systematic Review of Health Communication for Non-Cigarette Tobacco Products. | https://dx.doi.org/10.1080/10410236.2017.1407274 | Wrong outcome |
| Impact of e-cigarettes as cancer risk: A protocol for systematic review and meta-analysis. | https://dx.doi.org/10.1097/MD.0000000000032233 | Protocol |
| Association Between Exposure to Tobacco Content on Social Media and Tobacco Use: A Systematic Review and Meta-analysis. | https://dx.doi.org/10.1001/jamapediatrics.2022.2223 | Wrong exposure |
| Systematic review of systematic reviews: Do ecigarettes affect smoking cessation? | 10.1136/jim-2020-SRM.664 | Not systematic review |
| Human Biomarker Exposure From Cigarettes Versus Novel Heat-Not-Burn Devices: A Systematic Review and Meta-Analysis. | https://dx.doi.org/10.1093/ntr/ntz200 | Wrong exposure |
| Helping the quitters quit: A systematic review and narrative synthesis of the barriers and facilitators to e-cigarette cessation and the support that is needed. | https://dx.doi.org/10.1016/j.pec.2021.09.024 | Wrong outcome |
| Pulmonary effects of e-liquid flavors: a systematic review. | https://dx.doi.org/10.1080/10937404.2022.2124563 | Wrong exposure |
| Does the content and source credibility of health and risk messages related to nicotine vaping products have an impact on harm perception and behavioural intentions? A systematic review. | https://dx.doi.org/10.1111/add.15473 | Wrong exposure |
| Nicotine replacement therapy for long-term smoking cessation: a meta-analysis. | 10.1136/tc.2005.015487 | Wrong exposure |
| The rise of e-cigarettes, pod mod devices, and JUUL among youth: Factors influencing use, health implications, and downstream effects | 10.1016/j.drugalcdep.2019.04.011 | Wrong outcome |
| Carbonyl Emissions in E-cigarette Aerosol: A Systematic Review and Methodological Considerations. | https://dx.doi.org/10.3389/fphys.2017.01119 | Wrong outcome |
| Cigarette Smoke Exposure and Lung Health | 10.1002/ppul.25961 | Conference abstract |
| Particulate Matter from Electronic Cigarettes and Conventional Cigarettes: a Systematic Review and Observational Study. | https://dx.doi.org/10.1007/s40572-015-0072-x | Wrong outcome |
| The use of electronic cigarettes is not associated with cessation of smoking: A systematic review and meta-analysis | 10.1111/ijs.12633-2 | Conference abstract |
| Smoking Cessation in Patients With Acute Coronary Syndrome. | https://dx.doi.org/10.1016/j.amjcard.2018.01.017 | Wrong exposure |
| The Role of Nicotine and Flavor in the Abuse Potential and Appeal of Electronic Cigarettes for Adult Current and Former Cigarette and Electronic Cigarette Users: A Systematic Review. | 10.1093/ntr/ntac073 | Wrong exposure |
| Health Effects of Trace Metals in Electronic Cigarette Aerosols-a Systematic Review. | https://dx.doi.org/10.1007/s12011-018-1423-x | Wrong outcome |
| Cardiovascular Effects of Switching From Tobacco Cigarettes to Electronic Cigarettes. | https://dx.doi.org/10.1016/j.jacc.2019.09.067 | Primary study |
| Combustible and Electronic Cigarette Exposures Increase ACE2 Activity and SARS-CoV-2 Spike Binding. | 10.1164/rccm.202106-1377LE | letter to editor |
| EVALI (E-cigarette or Vaping use-Associated Lung Injury) and Youth Vaping | 10.1016/j.jnma.2020.09.072 | Not systematic review |
| Potential effects of using non-combustible tobacco and nicotine products during pregnancy: a systematic review. | https://dx.doi.org/10.1186/s12954-020-00359-2 | Wrong exposure |
| White matter development and tobacco smoking in young adults: A systematic review with recommendations for future research. | https://dx.doi.org/10.1016/j.drugalcdep.2016.02.015 | Wrong exposure |
| Reporting Certainty of Evidence on E-Cigarette Use for Adult Smoking Cessation. | https://dx.doi.org/10.2105/AJPH.2020.306091 | Not systematic review |
| PRS41 RESPIRATORY HEALTH EFFECTS OF E-CIGARETTES: A LITERATURE REVIEW | 10.1016/j.jval.2020.04.1362 | Abstract only |
| A scoping review of studies on the health impact of electronic nicotine delivery systems. | https://dx.doi.org/10.1007/s11739-021-02835-4 | Not systematic review |
| A systematic review of socio-ecological factors influencing current e-cigarette use among adolescents and young adults. | https://dx.doi.org/10.1016/j.addbeh.2022.107425 | Wrong exposure |
| Anesthesia Implications of Patient Use of Electronic Cigarettes. | | Not systematic review |
| Electronic cigarettes and subsequent cigarette smoking in young people | 10.1002/14651858.CD015170 | Protocol |
| Biomarkers of potential harm in people switching from smoking tobacco to exclusive e-cigarette use, dual use or abstinence: secondary analysis of Cochrane systematic review of trials of e-cigarettes for smoking cessation. | https://dx.doi.org/10.1111/add.16063 | Not systematic review |
| Interventions for preventing weight gain after smoking cessation. | https://dx.doi.org/10.1002/14651858.CD006219.pub4 | Wrong outcome |
| E-cigarettes and equity: a systematic review of differences in awareness and use between sociodemographic groups. | https://dx.doi.org/10.1136/tobaccocontrol-2016-053222 | Wrong outcome |
| Impact of Smoking and Vaping in Films on Smoking and Vaping Uptake in Adolescents: Systematic Review and Meta-Analysis. | 10.1177/10901981221086944 | Wrong exposure |
| Using the BMD Approach to Derive Acceptable Daily Intakes of Cannabidiol (CBD) and Tetrahydrocannabinol (THC) Relevant to Electronic Cigarette Liquids. | https://dx.doi.org/10.31083/j.fbl2708228 | Not systematic review |
| Effectiveness of smoking cessation interventions for smokers with chronic diseases: A systematic review. | 10.1111/jan.14869 | Wrong exposure |
| Systematic review on e-cigarette and its effects on weight gain and adipocytes. | https://dx.doi.org/10.1371/journal.pone.0270818 | Wrong outcome |
| Interventions for tobacco cessation delivered by dental professionals. | https://dx.doi.org/10.1002/14651858.CD005084.pub4 | Wrong exposure |
| Effect of nicotine on human gingival, periodontal ligament and oral epithelial cells. A systematic review of the literature. | https://dx.doi.org/10.1016/j.jdent.2019.05.030 | Wrong exposure |
| MS15.04 Approaching Cessation in the Patient Using Electronic Cigarettes | 10.1016/j.jtho.2019.08.370 | Abstract only |
| Electronic Cigarettes for Smoking Cessation. | | Abstract only |
| Smoking cessation interventions in the setting of low dose computed tomography (LDCT) lung cancer screening: A systematic review | 10.1164/ajrccm-conference.2017.D102 | Wrong intervention |
| Smoking cessation interventions for pregnant women attending treatment for substance use disorders: A systematic review. | https://dx.doi.org/10.1111/add.15663 | Wrong outcome |
| Prevalence and Associated Factors of E-Cigarette Use among Adolescents in Southeast Asia: A Systematic Review | 10.3390/ijerph20053883 | Wrong outcome |
| Interventions to Mitigate Vaping Misinformation: A Meta-Analysis. | https://dx.doi.org/10.1080/10810730.2022.2044941 | Wrong outcome |
| Effect of tobacco and nicotine in causing staining of dental hard tissues and dental materials: A systematic review and meta-analysis. | https://dx.doi.org/10.1002/cre2.683 | Systematic review of Invitro study |
| Impact of electronic cigarette smoking on the Saudi population through the analysis of literature: A systematic review | 10.4103/jomfp.JOMFP_141_19 | Wrong outcome |
| An International Systematic Review of Prevalence, Risk, and Protective Factors Associated with Young People's E-Cigarette Use. | https://dx.doi.org/10.3390/ijerph191811570 | Wrong outcome |
| A Systematic Review and Meta-analysis of the Association between E-cigarette Use among Cigarette Smokers and Quit Attempts Made to Abstain from Cigarette Smoking. | https://dx.doi.org/10.5993/AJHB.46.4.2 | Wrong outcome |
| Motivations for using electronic cigarettes in young adults: A systematic review. | https://dx.doi.org/10.1080/08897077.2019.1671937 | Wrong exposure |
| Is Some e-Cigarette Use, Like Some Cigarette Use, Chronic-Pain Coping? | 10.1080/00325481.2019.1655695 | Not systematic review |
| IQOS - a heat-not-burn (HnB) tobacco product - chemical composition and possible impact on oxidative stress and inflammatory response. A systematic review. | https://dx.doi.org/10.1080/15376516.2019.1669245 | Wrong exposure |
| Comparison of effects of tobacco cigarettes, electronic nicotine delivery systems and tobacco heating products on miRNA-mediated gene expression. A systematic review. | https://dx.doi.org/10.1080/15376516.2022.2089610 | Wrong outcome |
| Perceptions and experiences with flavored non-menthol tobacco products: A systematic review of qualitative studies | 10.3390/ijerph14040338 | Wrong exposure |
| An overview of the role of flavors in e-cigarette addiction | 10.18332/tid/84095 | Wrong exposure |
| Perceptions and Sentiments About Electronic Cigarettes on Social Media Platforms: Systematic Review. | https://dx.doi.org/10.2196/13673 | Wrong outcome |
| Association of e-cigarette use with smoking intentions and behaviors among adolescents: A systematic review | 10.6288/TJPH.201904_38(2).107136 | Not available in english |
| Is there a role for e-cigarettes in smoking cessation?. | https://dx.doi.org/10.1177/1753465815621233 | Not systematic review |
| Youth and Young Adult Use of Pod-Based Electronic Cigarettes From 2015 to 2019: A Systematic Review. | 10.1001/jamapediatrics.2020.0259 | Wrong outcome |
| Prevalence of Adolescent Cannabis Vaping: A Systematic Review and Meta-analysis of US and Canadian Studies. | 10.1001/jamapediatrics.2021.4102 | Wrong exposure |
| Radiological findings of EVALI: A systematic review | 10.1111/resp.14150 | Abstract only |
| An exploration of flavours in studies of e-cigarettes for smoking cessation: secondary analyses of a systematic review with meta-analyses. | https://dx.doi.org/10.1111/add.16091 | Wrong outcome |
| Nicotine receptor partial agonists for smoking cessation. | https://dx.doi.org/10.1002/14651858.CD006103.pub8 | Wrong exposure |
| A systematic review and meta-analysis of e-cigarette use among cancer survivors | 10.1007/s11764-023-01357-6 | Wrong outcome |
| Potential for non-combustible nicotine products to reduce socioeconomic inequalities in smoking: a systematic review and synthesis of best available evidence. | https://dx.doi.org/10.1186/s12889-019-7836-4 | Wrong outcome |
| Exploring factors associated with smokeless tobacco use among young people: A systematic scoping review | 10.1016/j.drugalcdep.2022.109627 | Wrong exposure |
| The up-rise in e-cigarette use - friend or foe? | 10.1186/s12931-016-0371-2 | Not systematic review |
| Exposure to Electronic Cigarette Advertisements and Use of Electronic Cigarettes: A Meta-analysis of Prospective Studies. | https://dx.doi.org/10.1093/ntr/ntac266 | Wrong exposure |
| Do Vaping Prevention Messages Impact Adolescents and Young Adults? A Meta-Analysis of Experimental Studies | 10.1080/10410236.2023.2185578 | Wrong exposure |
| Dual use of electronic cigarettes and classic cigarettes: a systematic review | 10.1080/16066359.2017.1388372 | Wrong exposure |
| [Prevalence of and risk factors for cigarette smoking among adolescents in South America: a systematic literature review]. | 10.1590/s1020-49892003000300004 | Wrong exposure |
| A Systematic Literature Review of Asian American, Native Hawaiian, and Pacific Islander Youth Electronic Nicotine Delivery Systems (ENDS) Use. | | Wrong outcome |
| Effectiveness of multicomponent interventions in primary healthcare settings to promote continuous smoking cessation in adults: a systematic review. | 10.1136/bmjopen-2015-008807 | Wrong exposure |
| Global frequency and epidemiological profile of electronic cigarette users: a systematic review. | 10.1016/j.oooo.2022.07.019 | Wrong outcome |
| Are vapes an effective device for smoking cessation or a gateway to conventional tobacco smoking? | | Wrong outcome |
| Electronic cigarettes for smoking cessation and reduction. | https://dx.doi.org/10.1002/14651858.CD010216.pub2 | Old version of updated cochrane review |
| Impact of non-menthol flavours in e-cigarettes on perceptions and use: an updated systematic review. | https://dx.doi.org/10.1136/bmjopen-2019-031598 | Wrong outcome |
| A critical analysis of 'Electronic cigarettes and health outcomes: Systematic review of global evidence'. | 10.1111/dar.13515 | Commentary |
| E-cigarette use in New Zealand-a systematic review and narrative synthesis. | | Wrong outcome |
| A systematic review of refillable e-liquid nicotine content accuracy. | https://dx.doi.org/10.1016/j.japh.2020.09.006 | Wrong outcome |
| Impact of smoking and smoking cessation on cardiovascular events and mortality among older adults: meta-analysis of individual participant data from prospective cohort studies of the CHANCES consortium. | 10.1136/bmj.h1551 | Wrong exposure |
| Burn Injury From Smoking Electronic Cigarettes While on Supplemental Oxygen | 10.1093/jbcr/irac087 | Wrong outcome |
| Water Pipe Tobacco Smoking and Risk of Coronary Artery Disease: A Systematic Review and Meta-Analysis. | 10.2174/1874467213666201223121322 | Wrong exposure |
| Effects of the vaper use in oral mucosa |  | Conference abstract |
| Efficacy and safety of pharmacotherapy for smoking cessation among pregnant smokers: a meta-analysis. | 10.1111/j.1471-0528.2012.03408.x | Wrong exposure |
| Vaping During Pregnancy: What Are the Potential Health Outcomes and Perceptions Pregnant Women Have?. | https://dx.doi.org/10.1016/j.jogc.2020.05.014 | Wrong outcome |
| E-Cigarettes and Cardiopulmonary Health: Review for Clinicians. | 10.1161/CIRCULATIONAHA.121.056777 | Not systematic review |
| Association between e-cigarette and atherosclerosis: A review | 10.1016/j.atherosclerosis.2022.06.245 | Abstract only |
| A systematic review examining the pulmonary effects of electronic vapor delivery systems. | https://dx.doi.org/10.1016/j.jclinane.2022.110952 | Wrong exposure |
| Urgent Need for Novel Investigations of Treatments to Quit E-cigarettes: Findings from a Systematic Review | 10.1158/1940-6207.CAPR-22-0172 | Wrong outcome |
| Instruments to measure e-cigarette related constructs: a systematic review. | 10.1186/s12889-022-13510-4 | Wrong outcome |
| The Convergence of the E-Cigarette or Vaping Associated Lung Injury (EVALI) Epidemic With the COVID-19 Pandemic: A Systematic Review | 10.1164/ajrccm-conference.2023.A51 | Conference abstract |
| Interventions for Tobacco Cessation in Adults, Including Pregnant Persons: Updated Evidence Report and Systematic Review for the US Preventive Services Task Force. | https://dx.doi.org/10.1001/jama.2020.23541 | Wrong exposure |
| Interventions for Tobacco Cessation in Adults, Including Pregnant Women: An Evidence Update for the U.S. Preventive Services Task Force | | Not systematic review |
| Behavioral Counseling and Pharmacotherapy Interventions for Tobacco Cessation in Adults, Including Pregnant Women: A Review of Reviews for the U.S. Preventive Services Task Force. | https://dx.doi.org/10.7326/M15-0171 | Wrong exposure |
| Exposure to second-hand smoke and the risk of tuberculosis in children and adults: a systematic review and meta-analysis of 18 observational studies. | 10.1371/journal.pmed.1001835 | Wrong exposure |
| Evaluation of periodontal indices among non-smokers, tobacco, and e-cigarette smokers: a systematic review and network meta-analysis. | https://dx.doi.org/10.1007/s00784-022-04531-9 | NMA |
| Smokers' Understandings of Addiction to Nicotine and Tobacco: A Systematic Review and Interpretive Synthesis of Quantitative and Qualitative Research. | https://dx.doi.org/10.1093/ntr/ntx186 | Wrong outcome |
| Correlates of tobacco use among Asian and Pacific Islander youth and young adults in the U.S.: A systematic review of the literature. | https://dx.doi.org/10.1037/pha0000511 | Wrong exposure |
| The Health Effects of Real-World Dual Use of Electronic and Conventional Cigarettes versus the Health Effects of Exclusive Smoking of Conventional Cigarettes: A Systematic Review. | https://dx.doi.org/10.3390/ijerph192013687 | Wrong exposure |
| [Effectiveness of smoking cessation interventions for pregnant women--meta-analyses of randomized trials and description of the study performed in Poland]. | | Wrong exposure |
| The rise of e-cigarettes: An emerging threat to the tobacco endgame? | | Conference abstract |
| Smoke and heart should stay apart: E cigarettes and other alternatives to conventional cigarettes' impact on cardiovascular health | 10.1136/jim-2021-SRMC.203 | Not systematic review |
| Regulatory Strategies for Preventing and Reducing Nicotine Vaping Among Youth: A Systematic Review. | 10.1016/j.amepre.2023.08.002 | Wrong outcome |
| Nicotine gateway effects on adolescent substance use | 10.5811/westjem.2019.7.41661 | Not systematic review |
| Viral Vaping: A systematic review and meta analysis of e-cigarette and Tobacco-Related social media content and its influence on youth behaviours and attitudes. | https://dx.doi.org/10.1016/j.addbeh.2023.107828 | Wrong exposure |
| E-cigarette explosion injuries in the oral and maxillofacial region and a protocol for their management | 10.1038/s41432-023-00936-x | Commentary |
| Flavor-Toxicant Correlation in E-cigarettes: A Meta-Analysis. | https://dx.doi.org/10.1021/acs.chemrestox.0c00247 | Wrong outcome |
| The effect of smoking and smoking cessation on arterial stiffness: a systematic review and meta-analysis. | https://dx.doi.org/10.1093/eurjcn/zvab102 | Wrong exposure |
| Smoking cessation for adolescents: a review of pharmacological and psychosocial treatments. | 10.2174/1874473710801020142 | Wrong exposure |
| Pharmacotherapy for smoking cessation: effects by subgroup defined by genetically informed biomarkers. | 10.1002/14651858.CD011823.pub2 | Wrong exposure |
| E-cigarette and vaping: Overview of product use related lung injury and its implications for medical & public health providers in the age of respiratory illness | 10.1111/ajad.13173 | Conference abstract |
| E-Cigarettes and myocardial Infarction: A systemic review and meta-analysis | 10.1093/eurjpc/zwab061.047 | Conference abstract |
| Adolescent's Health Perceptions of E-Cigarettes: A Systematic Review. | https://dx.doi.org/10.1016/j.amepre.2020.12.013 | Wrong outcome |
| 'Vaping'- a trojan horse against fight toward tobacco use and cancer: A systematic review of the existing evidence | 10.4103/ijmpo.ijmpo_11_20 | Wrong outcome |
| Heat-not-burn tobacco products: a systematic literature review. | https://dx.doi.org/10.1136/tobaccocontrol-2018-054419 | Wrong exposure |
| Exposure to E-cigarette adversely affects blood pressure and heart rate in healthy individuals: A systematic review and meta-analysis | | Conference abstract |
| Systematic review of behavioural smoking cessation interventions for older smokers from deprived backgrounds. | 10.1136/bmjopen-2019-032727 | Wrong exposure |
| Errors in data input in meta-analysis on association between initial use of e-cigarettes and subsequent cigarette smoking among adolescents and young adults | 10.1001/jamapediatrics.2017.4200 | Erratum |
| Intersection of smoking, e-cigarette use, obesity, and metabolic and bariatric surgery: a systematic review of the current state of evidence. | 10.1080/10550887.2021.1874817 | Wrong exposure |
| Efficacy of smoking-cessation interventions for young adults: a meta-analysis. | 10.1016/j.amepre.2012.02.013 | Wrong exposure |
| The Effects of E-Cigarette Vapor Components on the Morphology and Function of the Male and Female Reproductive Systems: A Systematic Review. | https://dx.doi.org/10.3390/ijerph17176152 | Systematic review of animal studies |
| Heated tobacco products for smoking cessation and reducing smoking prevalence. | https://dx.doi.org/10.1002/14651858.CD013790.pub2 | Wrong exposure |
| The prevalence of electronic cigarettes vaping globally: a systematic review and meta-analysis. | 10.1186/s13690-022-00998-w | Wrong outcome |
| Electronic cigarettes and cutaneous wound healing: A systematic review. | https://dx.doi.org/10.1016/j.jaad.2022.10.042 | letter to editor |
| Smoking cessation medicines and e-cigarettes: a systematic review, network meta-analysis and cost-effectiveness analysis | 10.3310/hta25590 | NMA |
| Comparative clinical effectiveness and safety of tobacco cessation pharmacotherapies and electronic cigarettes: a systematic review and network metaâ€analysis of randomized controlled trials. | 10.1111/add.15675 | NMA |
| The perceived effects of electronic cigarettes on health by adult users: A state of the science systematic literature review. | 10.1002/2327-6924.12358 | Wrong outcome |
| Chemical Profiles and Toxicity of Electronic Cigarettes: An Umbrella Review and Methodological Considerations. | https://dx.doi.org/10.3390/ijerph20031908 | Umbrella review |
| Interventions for smoking cessation and reduction in individuals with schizophrenia. | 10.1002/14651858.CD007253.pub2 | Wrong exposure |
| [Contribution of electronic cigarettes in smoking patients with psychotic disorders. A literature review]. | https://dx.doi.org/10.1016/j.encep.2020.11.008 | Wrong outcome |
| [Electronic cigarette use in patients with asthma]. | | Not available in english |
| Insomnia and parasomnia induced by validated smoking cessation pharmacotherapies and electronic cigarettes: A network meta-analysis | 10.1111/fcp.12908 | Conference abstract |
| [Efficacy and security of electronic cigarette for tobacco harm reduction: Systematic review and meta-analysis]. | https://dx.doi.org/10.1016/j.lpm.2016.05.026 | Not available in english |
| E-cigarette adverts and children's perceptions of tobacco smoking harms: an experimental study and meta-analysis. | https://dx.doi.org/10.1136/bmjopen-2017-020247 | Not systematic review |
| Effectiveness of smoking cessation interventions among pregnant women: An updated systematic review and meta-analysis. | 10.1016/j.addbeh.2023.107854 | Wrong exposure |
| Electronic cigarettes for smoking cessation: An individual patient meta-analysis of randomized controlled trials | 10.1183/13993003.congress-2019.OA5135 | Not systematic review |
| Cut down to quit' with nicotine replacement therapies in smoking cessation: a systematic review of effectiveness and economic analysis. | 10.3310/hta12020 | wrong outcome |
| Smoking by family members and friends and electronic-cigarette use in adolescence: A systematic review and meta-analysis. | https://dx.doi.org/10.18332/tid/84864 | Wrong outcome |
| Cigarette Smoking and Electronic Cigarettes Use: A Meta-Analysis. | https://dx.doi.org/10.3390/ijerph13010120 | Wrong outcome |
| Risk assessment of inhaled diacetyl from electronic cigarette use among teens and adults. | https://dx.doi.org/10.1016/j.scitotenv.2021.145486 | Not systematic review |
| Does utilization of electronic cigarettes facilitate smoking cessation compared to other interventions?. | | Not systematic review |
| The role of vitamin E acetate (VEA) and its derivatives in the vaping associated lung injury: systematic review of evidence. | https://dx.doi.org/10.1080/10408444.2020.1858754 | Wrong exposure |
| The influence of smoking and alcohol on bone healing: Systematic review and meta-analysis of non-pathological fractures. | https://dx.doi.org/10.1016/j.eclinm.2021.101179 | Wrong exposure |
| E-Cigarette Awareness, Use, and Harm Perception among Adults: A Meta-Analysis of Observational Studies. | https://dx.doi.org/10.1371/journal.pone.0165938 | Wrong outcome |
| A systematic review for the impacts of global approaches to regulating electronic nicotine products. | https://dx.doi.org/10.7189/jogh.13.04076 | Wrong outcome |
| A systematic review of anxiety across smoking stages in adolescents and young adults. | 10.1080/10826084.2019.1581222 | Wrong exposure |
| E-cigarette use and addiction | 10.1111/ajco.13868 | Conference abstract |
| Prevalence of electronic nicotine delivery systems and electronic non-nicotine delivery systems in children and adolescents: a systematic review and meta-analysis. | https://dx.doi.org/10.1016/S2468-2667(21)00106-7 | Wrong outcome |
| Prevalence of electronic nicotine delivery systems (ENDS) use among youth globally: a systematic review and meta-analysis of country level data. | https://dx.doi.org/10.1111/1753-6405.12777 | Wrong outcome |
| The effect of electronic cigarette use on peri-implant conditions in men: a systematic review and meta-analysis. | https://dx.doi.org/10.1016/j.oooo.2022.08.010 | Wrong outcome |
| A systematic review of consumer preference for e-cigarette attributes: Flavor, nicotine strength, and type | 10.1371/journal.pone.0194145 | Wrong outcome |
| Cigarette smoking and testosterone in men and women: A systematic review and meta-analysis of observational studies. | https://dx.doi.org/10.1016/j.ypmed.2015.12.021 | Wrong exposure |
| Acute effects of partial CB1 receptor agonists on cognition - A meta-analysis of human studies. | https://dx.doi.org/10.1016/j.pnpbp.2020.110063 | Wrong exposure |
| Exposure to Heated Tobacco Products and Adverse Health Effects, a Systematic Review. | https://dx.doi.org/10.3390/ijerph18126651 | Wrong exposure |
| Trend of electronic cigarette use among adult population in multiple countries across regions: A systematic review | | Wrong outcome |
| Electronic cigarettes: a systematic review of available studies on health risk assessment. | https://dx.doi.org/10.1515/reveh-2015-0075 | Wrong outcome |
| Erratum: Association between initial use of e-cigarettes and subsequent cigarette smoking among adolescents and young adults: a systematic review and meta-analysis (JAMA Pediatrics (2017) 171:8 (788-797) DOI: 10.1001/jamapediatrics.2017.1488) | 10.1001/jamapediatrics.2020.0480 | Erratum |
| Risk and safety profile of electronic nicotine delivery systems (ENDS): an umbrella review to inform ENDS health communication strategies. | https://dx.doi.org/10.1136/tc-2022-057495 | Umbrella review |
| E-cigarette use and combustible tobacco cigarette smoking uptake among non-smokers, including relapse in former smokers: Umbrella review, systematic review and meta-analysis | 10.1136/bmjopen-2020-045603 | Umbrella review |
| Electronic cigarettes and health outcomes: umbrella and systematic review of the global evidence. | https://dx.doi.org/10.5694/mja2.51890 | Umbrella review |
| Non-pharmacological interventions for smoking cessation: analysis of systematic reviews and meta-analyses. | https://dx.doi.org/10.1186/s12916-023-03087-z | Umbrella review |
| Vaping Cardiovascular Health Risks: an Updated Umbrella Review. | https://dx.doi.org/10.1007/s40138-020-00219-0 | Umbrella review |
| Health Effects of Electronic Cigarettes: An Umbrella Review and Methodological Considerations. | https://dx.doi.org/10.3390/ijerph19159054 | Umbrella review |
| Prospective association between use of electronic cigarettes and use of conventional cigarettes: a systematic review and meta-analysis. |  | Wrong outcome |
| Is adolescent e-cigarette use associated with smoking in the United Kingdom?: A systematic review with meta-analysis. Tobacco prevention & cessation |  | Wrong outcome |
| isk of smoking relapse with the use of electronic cigarettes: a systematic review with meta-analysis of longitudinal studies |  | Wrong outcome |
| Gateway or common liability? A systematic review and meta‐analysis of studies of adolescent e‐cigarette use and future smoking initiation. |  | Wrong outcome |
| Is e-cigarette use in non-smoking young adults associated with later smoking? A systematic review and meta-analysis. |  | Wrong outcome |
| Association between electronic cigarette use and tobacco cigarette smoking initiation in adolescents: a systematic review and meta-analysis. |  | Wrong outcome |
| Association between initial use of e-cigarettes and subsequent cigarette smoking among adolescents and young adults: a systematic review and meta-analysis |  | Wrong outcome |
| Association between electronic nicotine delivery systems and electronic non-nicotine delivery systems with initiation of tobacco use in individuals aged< 20 years. |  | Wrong outcome |
| Electronic cigarettes use and intention to cigarette smoking among never-smoking adolescents and young adults: a meta-analysis. International journal of environmental research and public health. |  | Wrong outcome |

**Table S5**: Amstar-2 quality assessment of the included systematic reviews

| **Study** | **Q1** | **Q2** | **Q3** | **Q4** | **Q5** | **Q6** | **Q7** | **Q8** | **Q9** | **Q10** | **Q11** | **Q12** | **Q13** | **Q14** | **Q15** | **Q16** |  |
| --- | --- | --- | --- | --- | --- | --- | --- | --- | --- | --- | --- | --- | --- | --- | --- | --- | --- |
|  | **Non-critical domain** | **Critical domain** | **Non-critical domain** | **Critical domain** | **Non-critical domain** | **Non-critical domain** | **Critical domain** | **Non-critical domain** | **Critical domain** | **Non-critical domain** | **Critical domain** | **Non-critical domain** | **Critical domain** | **Non-critical domain** | **Critical domain** | **Non-critical domain** | **Overall "critically low, low, medium, high"** |
| Anandan 2023 (1) | No | Yes | Yes | Yes | Yes | Yes | No | Yes | Yes | No | Yes | Yes | Yes | No | No | No | Critically Low |
| Ashour 2023 (2) | Yes | Yes | Yes | Yes | No | No | No | Yes | No | No | Not applicable | Not applicable | No | No | No | Yes | Critically Low |
| Awad 2023 (3) | No | No | Yes | Yes | No | No | No | Yes | Yes | No | Yes | Yes | Yes | Yes | No | No | Critically Low |
| Bandara 2023 (4) | Yes | Yes | Yes | Yes | Yes | No | No | Yes | Yes | No | Not applicable | Not applicable | Yes | No | No | Yes | Critically Low |
| Becker 2020 (5) | Yes | Yes | Yes | Yes | Yes | No | No | Yes | Yes | No | Not applicable | Not applicable | Yes | No | Not applicable | Yes | Low |
| Bjurlin 2020 (6) | Yes | Yes | Yes | Yes | Yes | Yes | No | Yes | Yes | No | Not applicable | Not applicable | Yes | Yes | Not applicable | Yes | Low |
| Bourke 2021 (7) | No | Yes | Yes | Yes | Yes | Yes | No | Yes | No | No | Not applicable | Not applicable | No | No | No | No | Critically Low |
| Bozier 2020 (8) | Yes | No | Yes | Yes | No | No | No | Yes | No | No | Not applicable | Not applicable | No | No | Not applicable | Yes | Critically Low |
| Bravo-Gutiérrez 2021 (9) | No | Yes | Yes | Yes | No | No | No | Yes | No | No | Not applicable | Not applicable | No | No | No | Yes | Critically Low |
| Calder 2021 (10) | Yes | Yes | Yes | Yes | Yes | Yes | No | Yes | Yes | Yes | Not applicable | Not applicable | Yes | No | Not applicable | Yes | Low |
| Chand 2022 (11) | Yes | Yes | Yes | Yes | Yes | Yes | No | Yes | Yes | Yes | Yes | Yes | Yes | Yes | Yes | Yes | Low |
| D Ambrosia 2022 (12) | Yes | Yes | Yes | Yes | Yes | No | Yes | Yes | Yes | No | Not applicable | Not applicable | Yes | No | Not applicable | Yes | Moderate |
| Damay 2022 (13) | No | No | No | Yes | No | No | No | Yes | Yes | No | Not applicable | Not applicable | Yes | No | No | No | Critically Low |
| Dekhou 2021 (14) | No | No | Yes | Yes | Yes | Yes | No | Yes | No | No | Not applicable | Not applicable | No | No | Not applicable | No | Critically Low |
| Farooqui 2023 (15) | No | No | Yes | Yes | Yes | Yes | No | Yes | No | No | Not applicable | Not applicable | No | No | Not applicable | No | Critically Low |
| Farsalinos 2014 (16) | No | No | No | Yes | No | No | No | Yes | No | No | Not applicable | Not applicable | No | No | Not applicable | Yes | Critically Low |
| Figueredo 2020 (17) | Yes | Yes | Yes | Yes | Yes | Yes | No | Yes | Yes | No | Yes | Yes | Yes | Yes | Not applicable | No | Low |
| Flach 2019 (18) | Yes | Yes | Yes | Yes | No | No | No | Yes | No | No | Not applicable | Not applicable | Yes | No | Not applicable | No | Critically Low |
| Garcia 2020 (19) | Yes | Yes | Yes | Yes | No | No | No | Yes | No | Yes | Yes | No | No | Yes | No | No | Critically Low |
| Gentry 2019 (20) | Yes | Yes | Yes | Yes | Yes | Yes | No | Yes | Yes | No | Not applicable | Not applicable | Yes | Yes | Not applicable | Yes | Low |
| Glasser 2017 (21) | Yes | No | No | Yes | No | No | No | Yes | No | No | Not applicable | Not applicable | No | No | Not applicable | Yes | Critically Low |
| Goniewicz 2020 (22) | Yes | Yes | Yes | Yes | Yes | Yes | Yes | Yes | Yes | No | Not applicable | Not applicable | No | Yes | Not applicable | Yes | Low |
| Gonsalves 2021 (23) | No | No | Yes | Yes | Yes | No | No | Yes | No | No | NA | NA | No | Yes | NA | Yes | Critically low |
| Gualano 2014 (24) | No | No | Yes | Yes | Yes | No | No | Yes | Yes | Yes | NA | NA | Yes | No | NA | No | Critically low |
| Guo 2022 (25) | Yes | Yes | No | Yes | Yes | Yes | No | Yes | Yes | No | Yes | Yes | Yes | Yes | Yes | Yes | Low |
| Hartmann-Boyce 2022 (26) | Yes | Yes | Yes | Yes | Yes | Yes | Yes | Yes | Yes | Yes | Yes | Yes | Yes | Yes | Yes | Yes | High |
| Hess et al 2016 (27) | Yes | No | No | Yes | No | No | No | Yes | No | Yes | NA | NA | No | No | NA | Yes | Critically low |
| Honeycutt 2022 (28) | No | Yes | Yes | Yes | Yes | Yes | No | Yes | Yes | No | NA | NA | Yes | Yes | NA | Yes | Low |
| Hua 2016 (29) | No | No | Yes | Yes | No | No | Yes | Yes | No | No | NA | NA | No | NA | NA | Yes | Critically low |
| Ioakeimidis 2016 (30) | No | No | No | Yes | No | No | No | No | No | No | No | No | No | No | No | No | Critically low |
| Keijsers 2022 (31) | Yes | Yes | Noe | Yes | Yes | No | No | Yes | Yes | No | NA | NA | Yes | Yes | NA | Yes | Low |
| Kennedy 2019 (32) | Yes | No | Yes | Yes | No | Yes | Yes | Yes | Yes | Yes | NA | NA | Yes | No | NA | No | Critically low |
| Kwon 2019 (33) | Yes | No | No | Yes | No | Yes | No | Yes | Yes | No | NA | NA | Yes | No | NA | Yes | Critically low |
| La Rosa 2023 (34) | Yes | Yes | Yes | Yes | Yes | Yes | No | Yes | Yes | No | NA | NA | Yes | Yes | NA | Yes | Low |
| Larue 2021 (35) | Yes | Yes | No | Yes | Yes | Yes | No | Yes | Yes | No | Yes | Yes | Yes | Yes | Yes | Yes | Low |
| Li 2022 (36) | Yes | Yes | No | Yes | Yes | Yes | No | Yes | Yes | No | Yes | Yes | Yes | Yes | Yes | Yes | Low |
| Liber 2023 (37) | Yes | No | No | Yes | Yes | Yes | No | Yes | No | No | NA | NA | Yes | No | NA | Yes | Critically low |
| Liu 2018 (38) | Yes | No | Yes | Yes | Yes | Yes | No | Yes | Yes | No | Yes | Yes | Yes | Yes | Yes | Yes | Critically low |
| Livingston 2022 (39) | Yes | No | Yes | Yes | Yes | Yes | No | Yes | Yes | No | NA | NA | No | No | NA | Yes | Critically low |
| Martinez-Morata 2020 (40) | Yes | No | Yes | No | No | No | No | Yes | No | No | NA | NA | No | No | NA | Yes | Critically low |
| Meng 2023 (41) | Yes | Yes | Yes | Yes | No | No | No | Yes | Yes | No | Yes | Yes | Yes | Yes | Yes | Yes | Low |
| Notley 2021 (42) | Yes | No | No | Yes | Yes | Yes | No | No | Yes | No | NA | NA | Yes | No | No | Yes | Critically low |
| Oloyede 2021 (43) | No | Yes | Yes | No | No | No | No | Yes | Yes | No | NA | NA | Yes | No | NA | Yes | Critically low |
| Patel et al 2020 (44) | No | No | No | No | No | No | No | Yes | No | No | NA | NA | No | Yes | NA | Yes | Critically low |
| Pesce et al 2022 (45) | Yes | Yes | Yes | Yes | Yes | Yes | Yes | Yes | Yes | No | Yes | No | Yes | Yes | No | Yes | Low |
| Pisinger et al 2014 (46) | Yes | No | Yes | Yes | No | No | Yes | Yes | Yes | Yes | Not applicable | Not applicable | Yes | Yes | Not applicable | Yes | Critically low |
| Prasetyo et al 2020 (47) | Yes | Yes | Yes | Yes | No | No | Yes | Yes | Yes | No | Not applicable | Not applicable | Yes | Yes | Not applicable | Yes | Critically low |
| Qureshi et al 2023 (48) | Yes | Yes | Yes | Yes | Yes | Yes | Yes | Yes | Yes | Yes | Not applicable | Yes | Yes | Yes | Not applicable | Yes | High |
| Rahman 2023 (49) | Yes | No | Yes | Yes | No | No | No | Yes | Yes | No | Yes | Yes | Yes | Yes | No | Yes | Critically low |
| Ralho et al 2019 (50) | Yes | Yes | Yes | Yes | Yes | Yes | No | Yes | Yes | Yes | Not applicable | Not applicable | Yes | Yes | No | No | Critically low |
| Riley et al 2015 (51) | Yes | No | Yes | Yes | Yes | Yes | No | Yes | Yes | Yes | Not applicable | Not applicable | Yes | Yes | No | No | Critically low |
| Scarpino 2020 (52) | Yes | No | Yes | Yes | Yes | Yes | No | Yes | No | No | Not applicable | Not applicable | Yes | No | No | Yes | Critically low |
| Seitz 2018 (53) | Yes | Yes | Yes | Yes | Yes | No | No | Yes | No | No | No | No | No | No | No | Yes | Critically low |
| Sharma 2022 (54) | Yes | Yes | Yes | Yes | Yes | Yes | Yes | Yes | Yes | No | Yes | Yes | No | Yes | No | Yes | Critically low |
| Siddiqi 2023 (55) | Yes | Yes | Yes | Yes | Yes | Yes | Yes | Yes | Yes | No | Yes | Yes | Yes | Yes | Yes | Yes | High |
| Skotsimara 2019 (56) | Yes | No | Yes | Yes | Yes | Yes | Yes | Yes | Yes | No | Yes | Yes | Yes | Yes | Yes | Yes | Medium |
| Sreedharan 2021 (57) | Yes | No | Yes | Yes | Yes | Yes | Yes | Yes | Yes | No | Not applicable | Not applicable | No | Yes | No | Yes | Critically low |
| Taylor 2023 (58) | Yes | Yes | Yes | Yes | Yes | Yes | Yes | Yes | Yes | Yes | Yes | Yes | Yes | Yes | No | Yes | Medium |
| Theim 2023 (59) | Yes | No | Yes | Yes | Yes | Yes | Yes | Yes | Yes | Yes | Yes | Yes | Yes | Yes | Yes | Yes | Medium |
| Tran 2023 (60) | Yes | No | Yes | Yes | Yes | Yes | No | Yes | Yes | No | No | No | Yes | Yes | No | Yes | Critically low |
| Tzortzi 2020 (61) | Yes | No | Yes | Yes | No | No | No | Yes | Not applicable | No | Yes | Not applicable | Not applicable | Not applicable | Yes | No | Critically low |
| Vanderkam 2022 (62) | Yes | Yes | Yes | Yes | Yes | Yes | Yes | Yes | Yes | No | Yes | Yes | Yes | Yes | No | Yes | Medium |
| Vyncke 2020 (63) | Yes | Yes | Yes | Yes | No | No | No | Yes | Yes | Yes | Not applicable | Not applicable | Not applicable | Yes | Yes | Yes | Critically low |
| Wasfi 2020 (64) | Yes | Yes | Yes | Yes | Yes | Yes | Yes | Yes | Yes | Yes | Not applicable | Not applicable | Yes | yes | Not applicable | No | Medium |
| Wills 2021 (65) | Yes | No | Yes | Yes | Yes | Yes | Yes | Yes | Yes | Yes | Yes | Yes | Yes | Yes | Yes | No | Low |
| Wilson 2022 (66) | Yes | No | Yes | Yes | Yes | Yes | Yes | Yes | Yes | Yes | Not applicable | Not applicable | Yes | Yes | Not applicable | Yes | Low |
| Xian 2020 (112) | Yes | No | Yes | Yes | Yes | Yes | Yes | Yes | Yes | Yes | Yes | Yes | Yes | Yes | Yes | No | Low |
| Yang 2020 (113) | Yes | No | Yes | Yes | Yes | Yes | Yes | Yes | Yes | Yes | Yes | Yes | Yes | Yes | Yes | No | Low |
| Zhao 2022 (114) | Yes | No | Yes | Yes | Yes | Yes | Yes | Yes | Yes | Yes | Yes | Yes | Yes | Yes | Yes | No | Low |

**Figure S1 . Adv Events E cigarettes VS NRT**

**
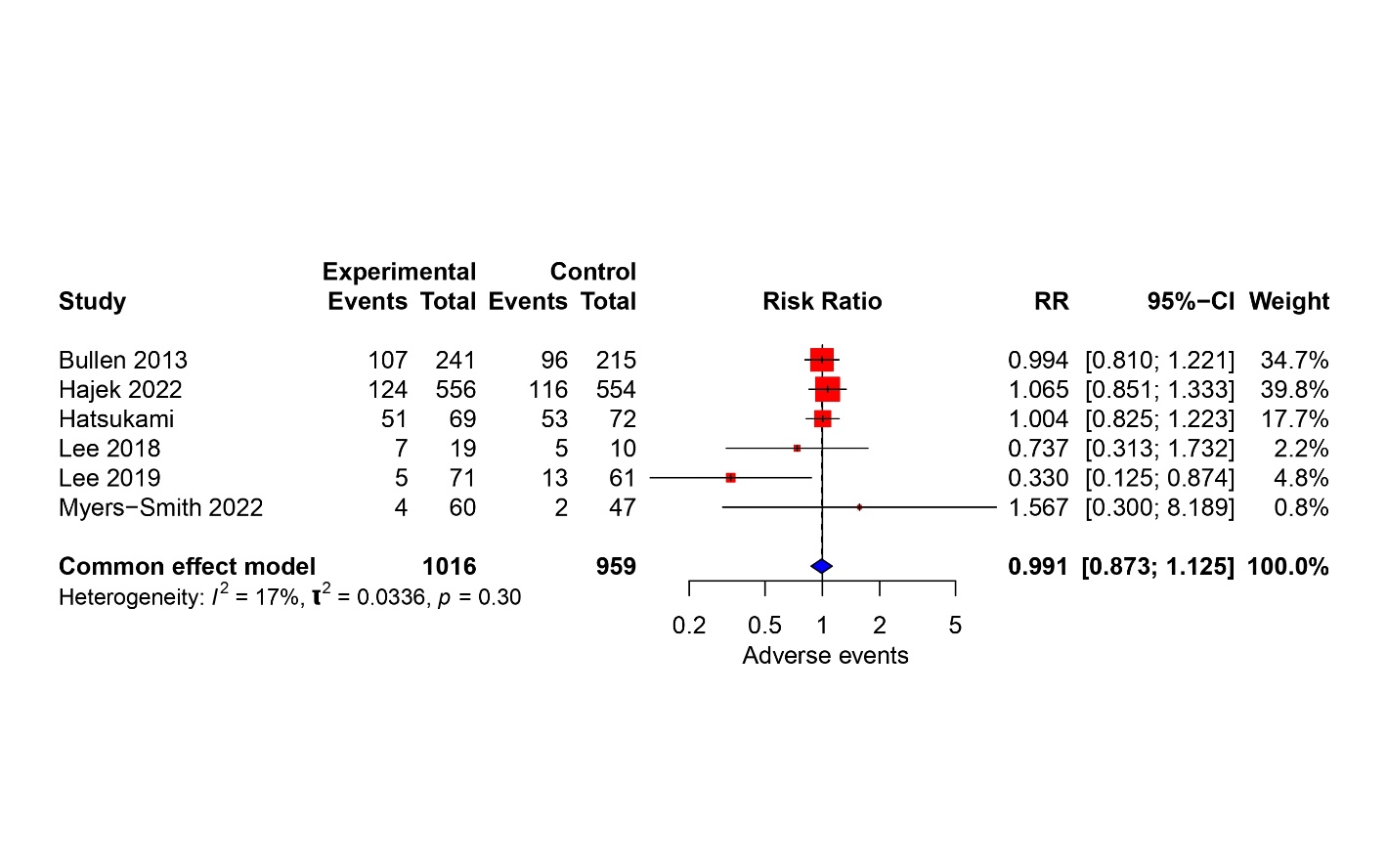
**

**Figure S2 . Adverse Events E cigarettes VS Non-nicotine e-cigarettes**

**
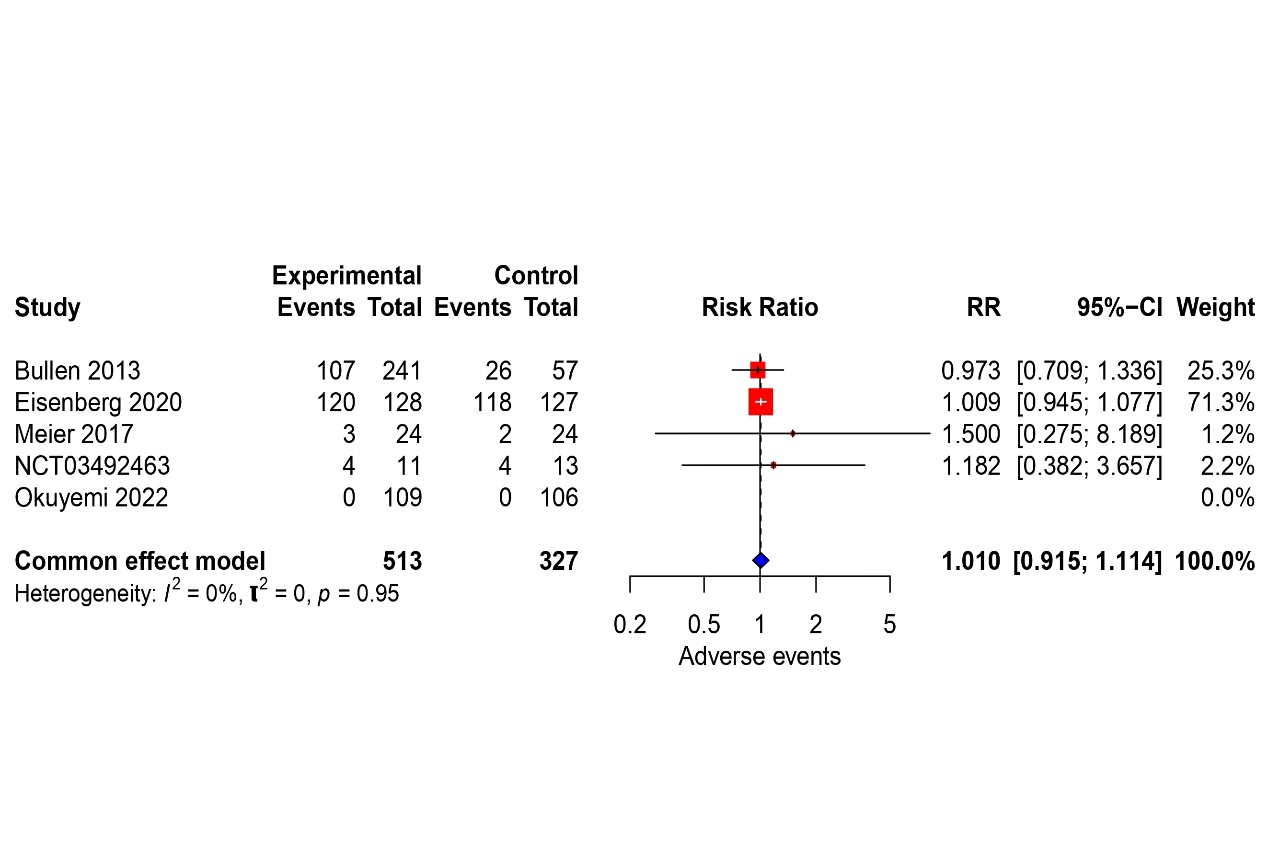
**

**Figure S3. Serious adverse events: E cigarettes VS NRT**

**
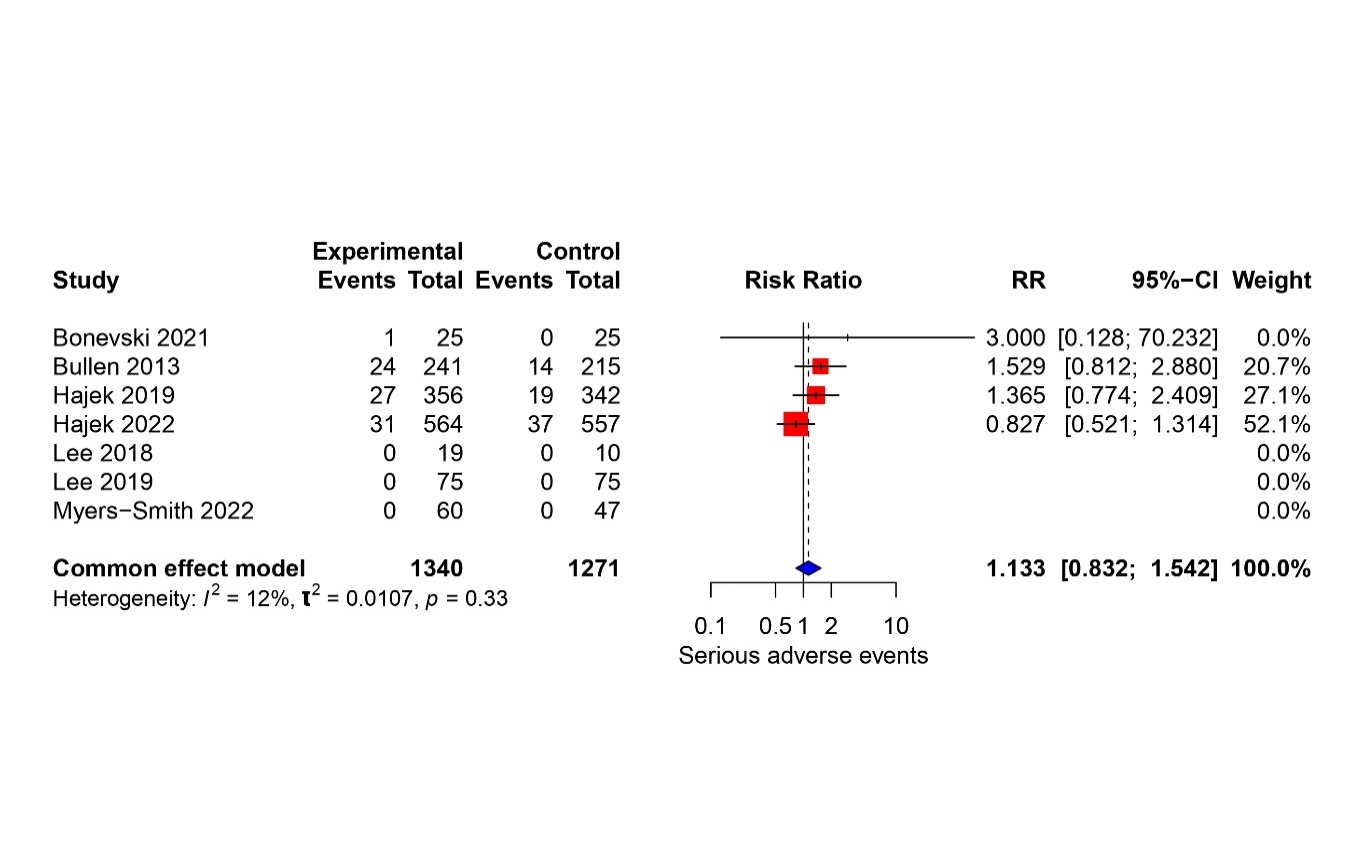
**

**Figure S4 Serious adverse events: E cigarettes VS Non-nicotine e-cigarettes**

**
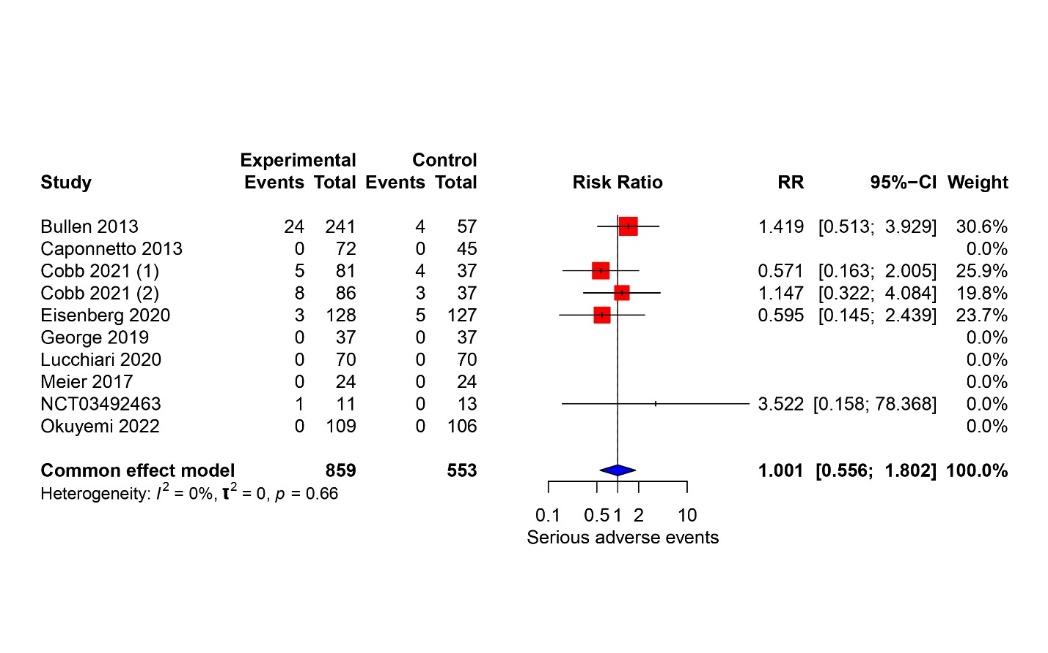
**

**Figure S5. Heart rate Non Smokers Vs Smokers**

**
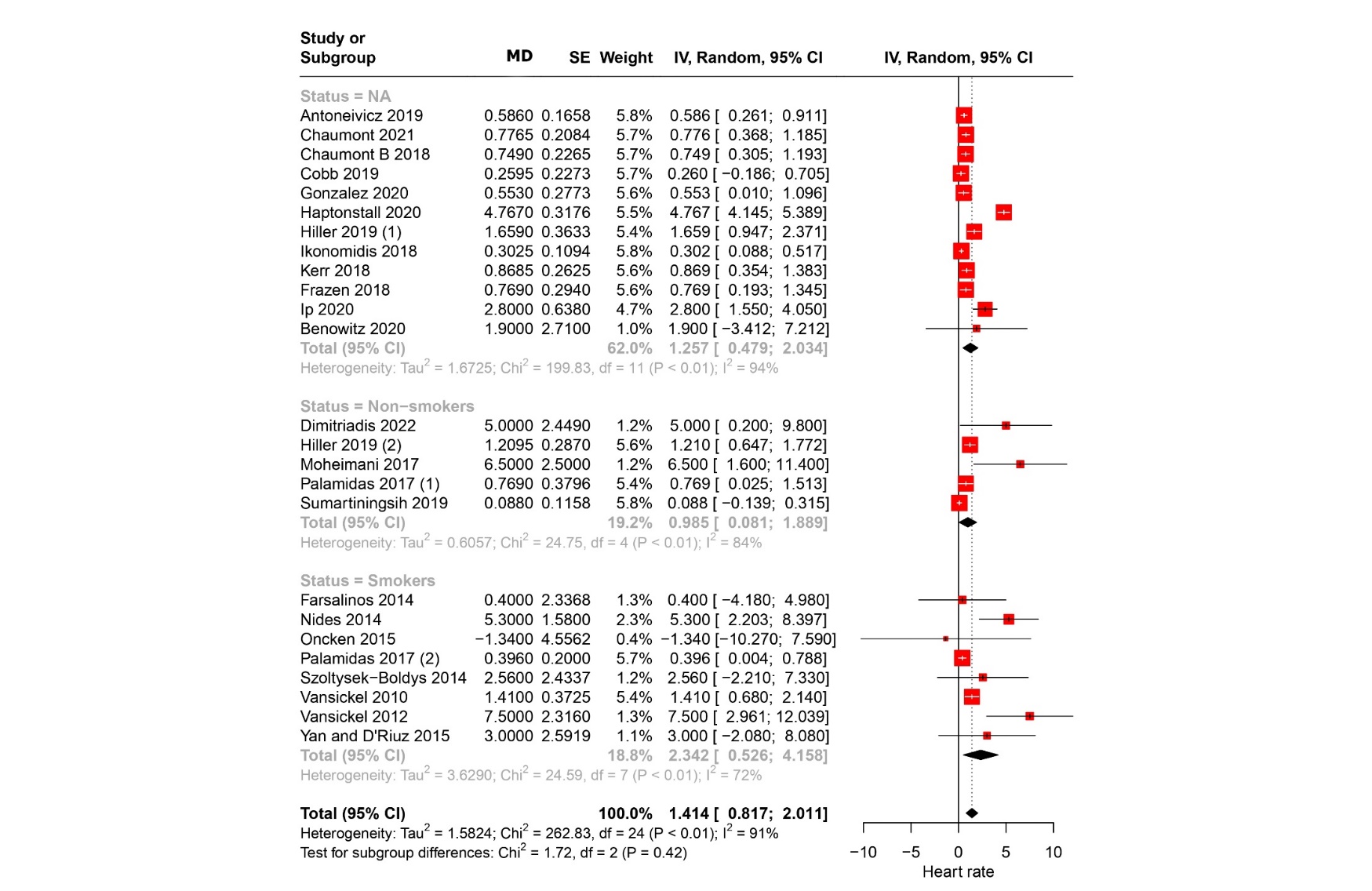
**

**Figure S6 Systolic Blood Pressure Non Smokers Vs Smokers**

**
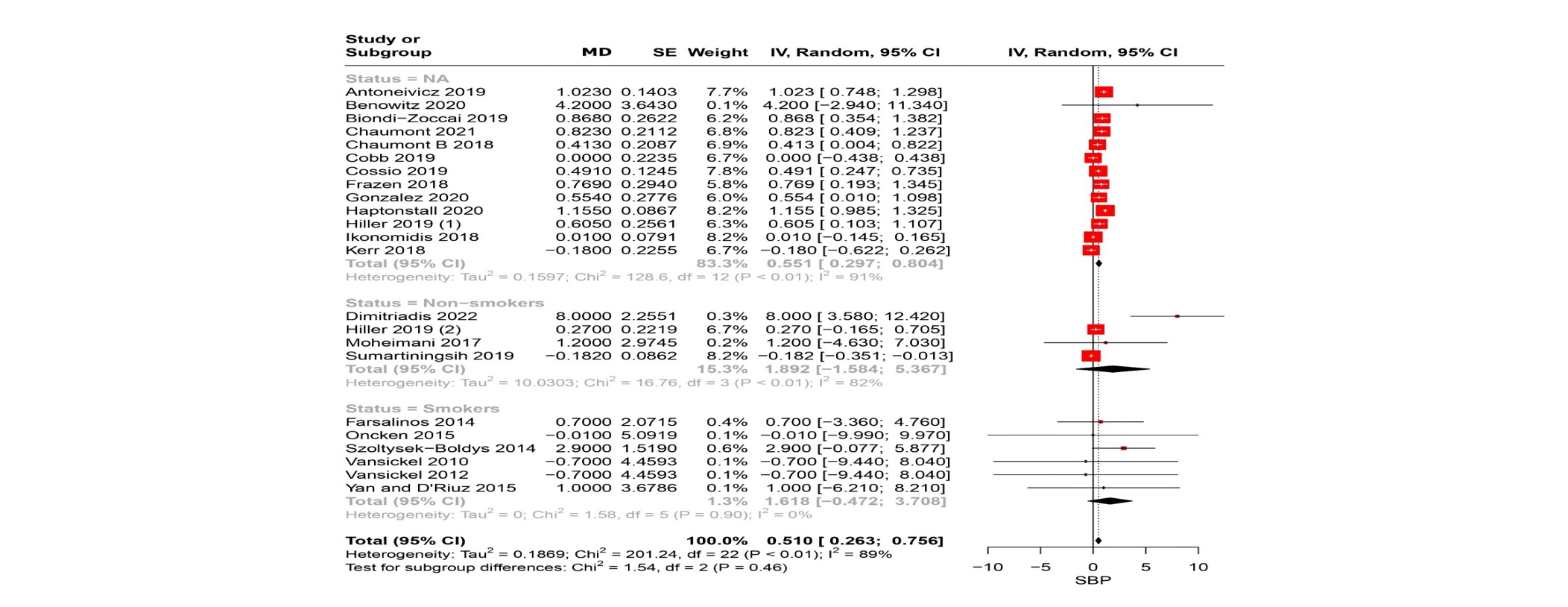
**

**Figure S7. Diastolic Blood Pressure**

**
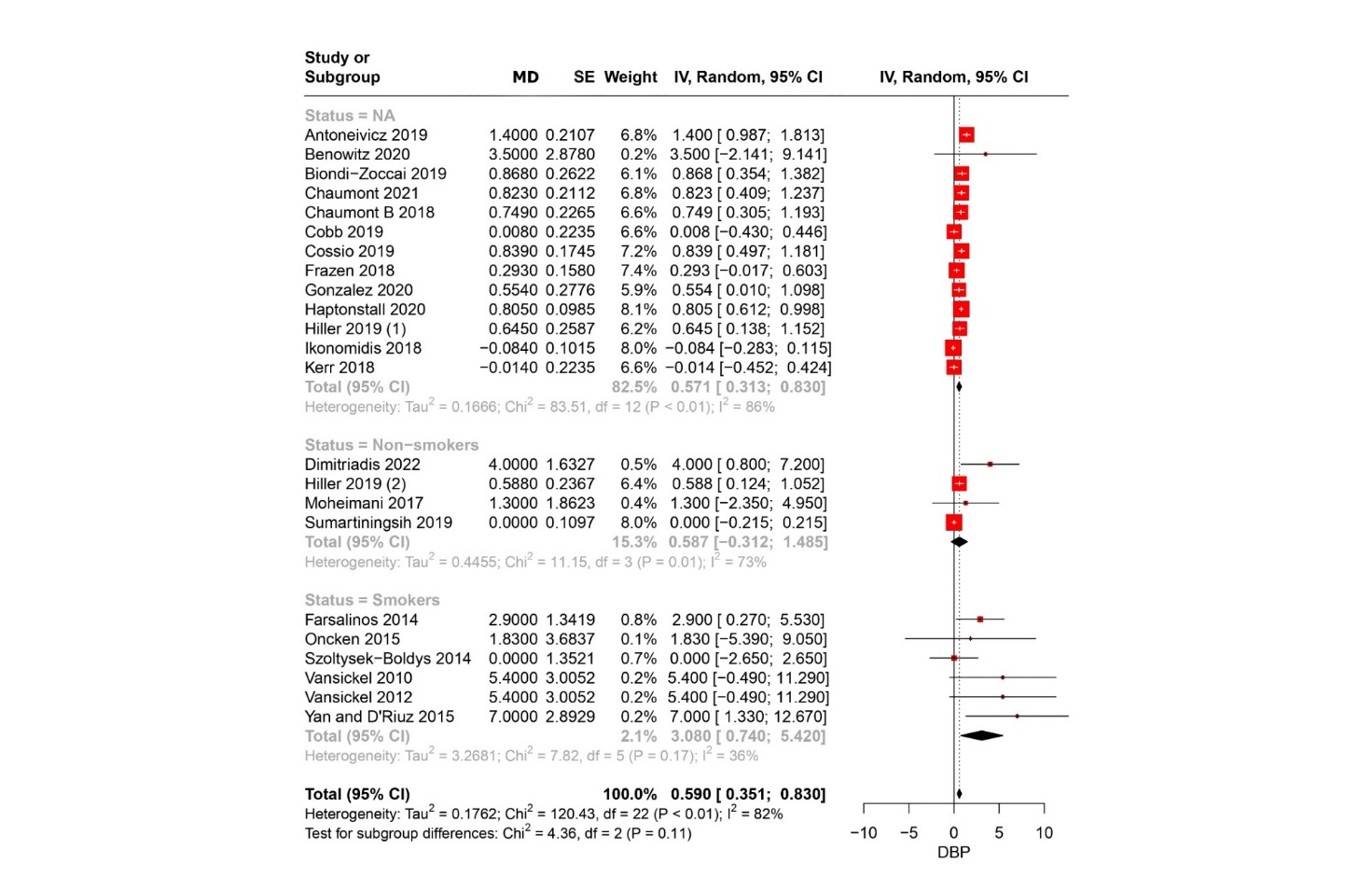
**

**Figure S8. Publication bias assessment-Funnel plots and trim & Fill and Heart rate**


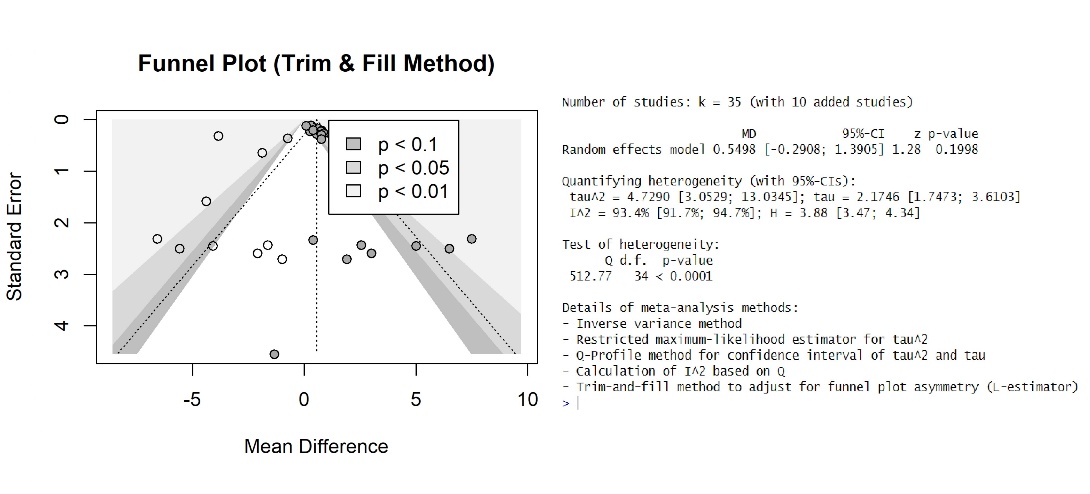


**Figure S9. Publication bias assessment-Funnel plots and trim & Fill for SBP**


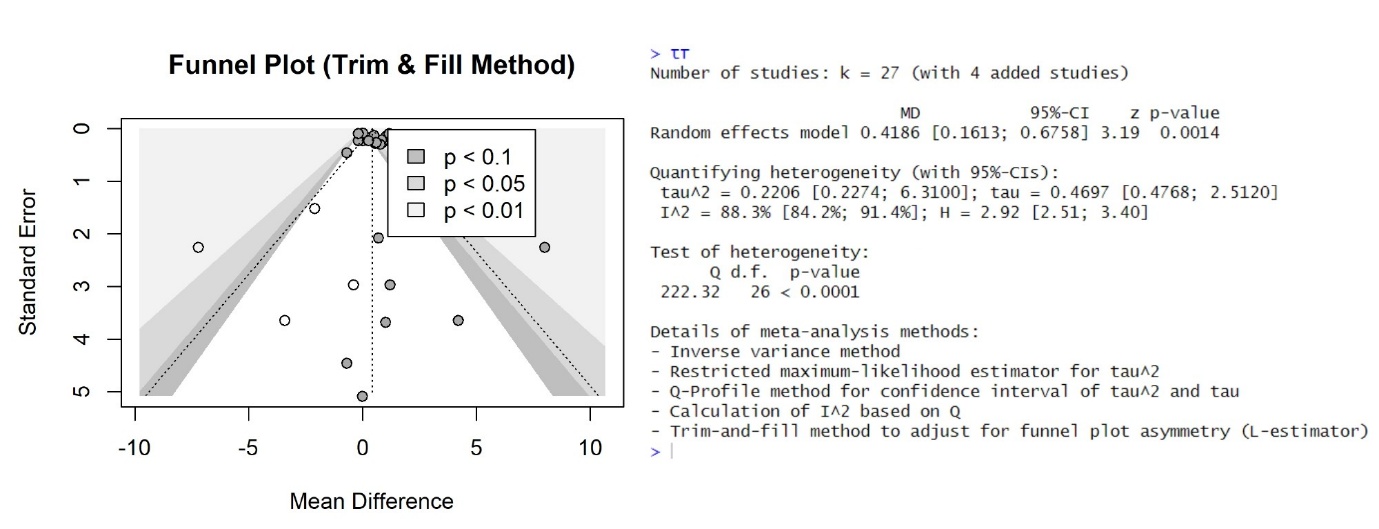


**Figure S10. Publication bias assessment-Funnel plots and trim & Fill for DBP**


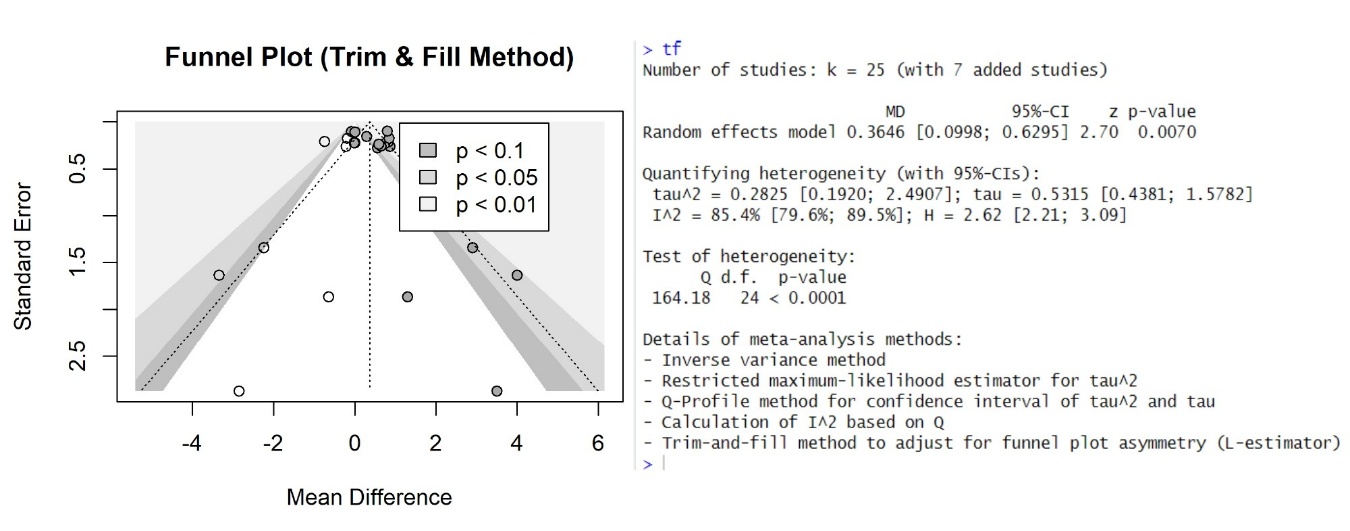


**References of included systematic reviews**

1. Anandan AS, Leung J, Chan GC, Sun T, Connor JP, Hall WD, et al. Common adverse events of electronic cigarettes compared with traditional nicotine replacement therapies: A systematic review and meta‐analysis. Drug and Alcohol Review. 2023.

2. Ashour O, Al-Huneidy L, Noordeen H. The implications of vaping on surgical wound healing: A systematic review. Surgery. 2023.

3. Awad K, Mohammed M, Martin SS, Banach M. Association between electronic nicotine delivery systems use and risk of stroke: a meta-analysis of 1,024,401 participants. Archives of Medical Science: AMS. 2023;19(5):1538.

4. Bandara NA, Zhou XR, Alhamam A, Black PC, St-Laurent M-P. The genitourinary impacts of electronic cigarette use: A systematic review of the literature. World Journal of Urology. 2023;41(10):2637-46.

5. Becker TD, Arnold MK, Ro V, Martin L, Rice TR. Systematic review of electronic cigarette use (vaping) and mental health comorbidity among adolescents and young adults. Nicotine and Tobacco Research. 2021;23(3):415-25.

6. Bjurlin MA, Matulewicz RS, Roberts TR, Dearing BA, Schatz D, Sherman S, et al. Carcinogen biomarkers in the urine of electronic cigarette users and implications for the development of bladder cancer: a systematic review. European urology oncology. 2021;4(5):766-83.

7. Bourke M, Sharif N, Narayan O. Association between electronic cigarette use in children and adolescents and coughing a systematic review. Pediatric Pulmonology. 2021;56(10):3402-9.

8. Bozier J, Chivers EK, Chapman DG, Larcombe AN, Bastian NA, Masso-Silva JA, et al. The evolving landscape of e-cigarettes: a systematic review of recent evidence. Chest. 2020;157(5):1362-90.

9. Bravo-Gutiérrez OA, Falfán-Valencia R, Ramírez-Venegas A, Sansores RH, Ponciano-Rodríguez G, Pérez-Rubio G. Lung damage caused by heated tobacco products and electronic nicotine delivery systems: a systematic review. International journal of environmental research and public health. 2021;18(8):4079.

10. Calder R, Gant E, Bauld L, McNeill A, Robson D, Brose LS. Vaping in pregnancy: a systematic review. Nicotine and Tobacco Research. 2021;23(9):1451-8.

11. Chand BR, Hosseinzadeh H. Association between e-cigarette use and asthma: a systematic review and meta-analysis. Journal of Asthma. 2022;59(9):1722-31.

12. D’Ambrosio F, Pisano M, Amato A, Iandolo A, Caggiano M, Martina S. Periodontal and peri-implant health status in traditional vs. heat-not-burn tobacco and electronic cigarettes smokers: A systematic review. Dentistry Journal. 2022;10(6):103.

13. Damay VA, Setiawan S, Lesmana R, Akbar MR, Lukito AA. How Electronic Cigarette Affects the Vascular System. Journal of Smoking Cessation. 2022;2022.

14. Dekhou A, Oska N, Partiali B, Johnson J, Chung MT, Folbe A. E-cigarette burns and explosions: what are the patterns of oromaxillofacial injury? Journal of Oral and Maxillofacial Surgery. 2021;79(8):1723-30.

15. Farooqui M, Shoaib S, Afaq H, Quadri S, Zaina F, Baig A, et al. Bidirectionality of smoking and depression in adolescents: a systematic review. Trends in Psychiatry and Psychotherapy. 2023;45:e20210429.

16. Farsalinos KE, Poulas K, Voudris V, Le Houezec J. Electronic cigarette use in the European Union: analysis of a representative sample of 27 460 Europeans from 28 countries. Addiction. 2016;111(11):2032-40.

17. Figueredo CA, Abdelhay N, Figueredo CM, Catunda R, Gibson MP. The impact of vaping on periodontitis: A systematic review. Clinical and Experimental Dental Research. 2021;7(3):376-84.

18. Flach S, Maniam P, Manickavasagam J. E‐cigarettes and head and neck cancers: A systematic review of the current literature. Clinical Otolaryngology. 2019;44(5):749-56.

19. Garcia PD, Gornbein JA, Middlekauff HR. Cardiovascular autonomic effects of electronic cigarette use: a systematic review. Clinical Autonomic Research. 2020;30:507-19.

20. Gentry S, Forouhi NG, Notley C. Are electronic cigarettes an effective aid to smoking cessation or reduction among vulnerable groups? A systematic review of quantitative and qualitative evidence. Nicotine and Tobacco Research. 2019;21(5):602-16.

21. Glasser AM, Collins L, Pearson JL, Abudayyeh H, Niaura RS, Abrams DB, et al. Overview of electronic nicotine delivery systems: a systematic review. American journal of preventive medicine. 2017;52(2):e33-e66.

22. Goniewicz ML, Miller CR, Sutanto E, Li D. How effective are electronic cigarettes for reducing respiratory and cardiovascular risk in smokers? A systematic review. Harm reduction journal. 2020;17(1):1-9.

23. Gonsalves CL, Zhu JW, Kam AJ. Diagnosis and acute management of E-cigarette or vaping product use–associated lung injury in the pediatric population: a systematic review. The Journal of Pediatrics. 2021;228:260-70.

24. Gualano MR, Passi S, Bert F, La Torre G, Scaioli G, Siliquini R. Electronic cigarettes: assessing the efficacy and the adverse effects through a systematic review of published studies. Journal of Public Health. 2015;37(3):488-97.

25. Guo J, Hecht SS. DNA damage in human oral cells induced by use of e‐cigarettes. Drug Testing and Analysis. 2022.

26. Hartmann-Boyce J, Lindson N, Butler AR, McRobbie H, Bullen C, Begh R, et al. Electronic cigarettes for smoking cessation. Cochrane database of systematic reviews. 2022(11).

27. Hess IM. A systematic review of the health risks from passive exposure to electronic cigarette vapour. Public health research and practice. 2016;26(2).

28. Honeycutt L, Huerne K, Miller A, Wennberg E, Filion KB, Grad R, et al. A systematic review of the effects of e-cigarette use on lung function. NPJ Primary Care Respiratory Medicine. 2022;32(1):45.

29. Hua M, Talbot P. Potential health effects of electronic cigarettes: a systematic review of case reports. Preventive medicine reports. 2016;4:169-78.

30. Ioakeimidis N, Vlachopoulos C, Tousoulis D. Efficacy and safety of electronic cigarettes for smoking cessation: a critical approach. Hellenic J Cardiol. 2016;57(1):1-6.

31. Keijsers M, Vega-Corredor MC, Hoermann S, Tomintz M. Cue reactivity to electronic cigarettes: A systematic review. Substance Abuse: Research and Treatment. 2022;16:11782218221114971.

32. Kennedy RD, Awopegba A, De León E, Cohen JE. Global approaches to regulating electronic cigarettes. Tobacco control. 2017;26(4):440-5.

33. Kwon M, Park E, Dickerson SS. Adolescent substance use and its association to sleep disturbances: A systematic review. Sleep health. 2019;5(4):382-94.

34. La Rosa G, Vernooij R, Qureshi M, Polosa R, O’Leary R. Clinical testing of the cardiovascular effects of e-cigarette substitution for smoking: a living systematic review. Internal and Emergency Medicine. 2023;18(3):917-28.

35. Larue F, Tasbih T, Ribeiro PA, Lavoie KL, Dolan E, Bacon SL. Immediate physiological effects of acute electronic cigarette use in humans: A systematic review and meta-analysis. Respiratory medicine. 2021;190:106684.

36. Li X, Zhang Y, Zhang R, Chen F, Shao L, Zhang L. Association between e-cigarettes and asthma in adolescents: a systematic review and meta-analysis. American Journal of Preventive Medicine. 2022;62(6):953-60.

37. Liber AC, Knoll M, Cadham CJ, Issabakhsh M, Oh H, Cook S, et al. The role of flavored electronic nicotine delivery systems in smoking cessation: A systematic review. Drug and Alcohol Dependence Reports. 2023:100143.

38. Liu X, Lu W, Liao S, Deng Z, Zhang Z, Liu Y, et al. Efficiency and adverse events of electronic cigarettes: a systematic review and meta-analysis (PRISMA-compliant article). Medicine. 2018;97(19).

39. Livingston JA, Chen C-H, Kwon M, Park E. Physical and mental health outcomes associated with adolescent E-cigarette use. Journal of pediatric nursing. 2022;64:1-17.

40. Martinez-Morata I, Sanchez TR, Shimbo D, Navas-Acien A. Electronic cigarette use and blood pressure endpoints: a systematic review. Current hypertension reports. 2021;23:1-10.

41. Meng X-c, Guo X-x, Peng Z-y, Wang C, Liu R. Acute effects of electronic cigarettes on vascular endothelial function: a systematic review and meta-analysis of randomized controlled trials. European Journal of Preventive Cardiology. 2023;30(5):425-35.

42. Notley C, Gentry S, Cox S, Dockrell M, Havill M, Attwood AS, et al. Youth use of e‐liquid flavours—a systematic review exploring patterns of use of e‐liquid flavours and associations with continued vaping, tobacco smoking uptake or cessation. Addiction. 2022;117(5):1258-72.

43. Oloyede EO, Ola O, Kolade VO, Tevie J, OLOYEDE E, OLA O, et al. Looking Back and Going Forward: Roles of Varenicline and Electronic Cigarettes in Smoking Cessation. Cureus. 2021;13(8).

44. Patel S, Wooles N, Martin T. A systematic review of the impact of cigarettes and electronic cigarettes in otology. The Journal of Laryngology & Otology. 2020;134(11):951-6.

45. Pesce P, Menini M, Ugo G, Bagnasco F, Dioguardi M, Troiano G. Evaluation of periodontal indices among non-smokers, tobacco, and e-cigarette smokers: A systematic review and network meta-analysis. Clinical Oral Investigations. 2022;26(7):4701-14.

46. Pisinger C, Døssing M. A systematic review of health effects of electronic cigarettes. Preventive medicine. 2014;69:248-60.

47. Prasetyo A, Sadhana U, Budiman J. Nasal mucociliary clearance in smokers: a systematic review. International Archives of Otorhinolaryngology. 2021;25:160-9.

48. Qureshi MA, Vernooij RW, La Rosa GRM, Polosa R, O’Leary R. Respiratory health effects of e-cigarette substitution for tobacco cigarettes: a systematic review. Harm Reduction Journal. 2023;20(1):143.

49. Rahman A, Alqaisi S, Alzakhari R, Saith S. Characterization and Summarization of the Impact of Electronic Cigarettes on the Cardiovascular System: A Systematic Review and Meta-Analysis. Cureus. 2023;15(5).

50. Ralho A, Coelho A, Ribeiro M, Paula A, Amaro I, Sousa J, et al. Effects of electronic cigarettes on oral cavity: a systematic review. Journal of Evidence Based Dental Practice. 2019;19(4):101318.

51. Riley HE, Berry-Bibee E, England LJ, Jamieson DJ, Marchbanks PA, Curtis KM. Hormonal contraception among electronic cigarette users and cardiovascular risk: a systematic review. Contraception. 2016;93(3):190-208.

52. Scarpino M, Rosso T, Lanzo G, Lolli F, Bonizzoli M, Lazzeri C, et al. Severe neurological nicotine intoxication by e‐cigarette liquids: Systematic literature review. Acta Neurologica Scandinavica. 2021;143(2):121-30.

53. Seitz CM, Kabir Z. Burn injuries caused by e-cigarette explosions: A systematic review of published cases. Tobacco prevention & cessation. 2018;4.

54. Sharma A, Gupta I, Venkatesh U, Singh AK, Golamari R, Arya P. E-cigarettes and myocardial infarction: A systematic review and meta-analysis. International Journal of Cardiology. 2023;371:65-70.

55. Siddiqi TJ, Rashid AM, Siddiqi AK, Anwer A, Usman MS, Sakhi H, et al. Association of Electronic Cigarette Exposure on Cardiovascular Health: A Systematic Review and Meta-Analysis. Current Problems in Cardiology. 2023:101748.

56. Skotsimara G, Antonopoulos AS, Oikonomou E, Siasos G, Ioakeimidis N, Tsalamandris S, et al. Cardiovascular effects of electronic cigarettes: a systematic review and meta-analysis. European journal of preventive cardiology. 2019;26(11):1219-28.

57. Sreedharan S, Mian M, Robertson RA, Rhodes A. Radiological findings of e-cigarette or vaping product use associated lung injury: A systematic review. Heart & Lung. 2021;50(5):736-41.

58. Taylor E, Simonavičius E, McNeill A, Brose LS, East K, Marczylo T, et al. Exposure to Tobacco Specific Nitrosamines among people who vape, smoke or do neither. A Systematic Review and Meta Analysis. Nicotine and Tobacco Research. 2023:ntad156.

59. Thiem DG, Donkiewicz P, Rejaey R, Wiesmann-Imilowski N, Deschner J, Al-Nawas B, et al. The impact of electronic and conventional cigarettes on periodontal health—a systematic review and meta-analysis. Clinical oral investigations. 2023;27(9):4911-28.

60. Tran V, Mian M, Sreedharan S, Robertson R, Saha A, Tadakamadla SK, et al. Oral and maxillofacial injuries associated with e-cigarette explosions: a systematic review and management guidelines proposal. Journal of Oral and Maxillofacial Surgery. 2023.

61. Tzortzi A, Kapetanstrataki M, Evangelopoulou V, Behrakis P. A systematic literature review of e-cigarette-related illness and injury: not just for the respirologist. International journal of environmental research and public health. 2020;17(7):2248.

62. Vanderkam P, Bonneau A, Kinouani S, Dzeraviashka P, Castera P, Besnier M, et al. Duration of the effectiveness of nicotine electronic cigarettes on smoking cessation and reduction: Systematic review and meta-analysis. Frontiers in Psychiatry. 2022:1721.

63. Vyncke T, De Wolf E, Hoeksema H, Verbelen J, De Coninck P, Buncamper M, et al. Injuries associated with electronic nicotine delivery systems: A systematic review. Journal of trauma and acute care surgery. 2020;89(4):783-91.

64. Wasfi RA, Bang F, de Groh M, Champagne A, Han A, Lang JJ, et al. Chronic health effects associated with electronic cigarette use: A systematic review. Frontiers in public health. 2022;10:959622.

65. Wills TA, Soneji SS, Choi K, Jaspers I, Tam EK. E-cigarette use and respiratory disorders: an integrative review of converging evidence from epidemiological and laboratory studies. European Respiratory Journal. 2021;57(1).

66. Wilson C, Tellez Freitas CM, Awan KH, Ajdaharian J, Geiler J, Thirucenthilvelan P. Adverse effects of e‐cigarettes on head, neck, and oral cells: a systematic review. Journal of Oral Pathology & Medicine. 2022;51(2):113-25.
