# Supplementary material for: Health impacts of electronic nicotine delivery systems: an umbrella review of systematic reviews
Source: BMJ Open. 2025 Oct 10;15(10):e100168. doi: 10.1136/bmjopen-2025-100168 (PMC12516984; doi:10.1136/bmjopen-2025-100168)
Supplement: Supplementary file 2 [file bmjopen-2025-100168.reviewer_comments.pdf]

PEER REVIEW HISTORY

BMJ Open publishes all reviews undertaken for accepted manuscripts. Reviewers are asked to complete a checklist review form and are provided with free text boxes to elaborate on their assessment. These free text comments are reproduced below.

ARTICLE DETAILS

Title (Provisional)

Health Impacts of Electronic Nicotine Delivery Systems: An Umbrella Review of Systematic Reviews

Authors

Kaur, Jagdish; Goel, Sonu; Shabil, Muhammed; Rana, Rishabh Kumar; Chauhan, Anil; Gupta, Shibaji; Rinkoo, Arvind Vashishta

VERSION 1 - REVIEW

**Reviewer**	**1**
**Name**	**Watson, Peter**
**Affiliation Unit**	**University of Cambridge, MRC Cognition and Brain Sciences**
**Date**	**28-Apr-2025**
**COI**	**None**

Health impacts of electronic nicotine delivery systems: an umbrella review of systematic reviews bmjopen-2025-100168

I make a few comments concerning improved, or indeed a lack of, assessment for publication bias, possible lack of clinically meaningful differences, incorporation and more detailed discussion of the high between study heterogeneity and inconsistencies in the tables.

Line 205. I would state here which routine you used in R for the meta-analysis e.g. metafor?

Line 208. I would replace the wording with "low heterogeneity" for 0%-40%.

Lines 210-211. Rather than a visual inspection of the funnel plots to assess asymmetry of publication bias you should use Duval and Tweedie's (2000) more objective trim and fill procedure which will assess asymmetry and in addition add in any extra studies to any asymmetric plots to give an unbiased effect size.

Table 3. You appear to give the total sample size in the third column. I think you should split this and quote the sample sizes for each group being compared by the effect size e.g. for E-cigarettes and NRT for the Relative Risk in row 1. I also think you should state in the comparison columns

of table 3 and 4 in each row which groups are being compared to yield the effect size in that row. You mention which groups are being compared for some rows but not for the majority.

Table 3. Could you explain why you use a mean difference for cardiac parameters in table 3? The usual effect size for a comparisons of means is the standardised mean difference or Cohen's d which you later use for the respiratory parameters and, as such, is more understandable that the raw difference in means as it is scale invariant. It may be the mean differences you have found for the cardiac parameters are too small to be not clinically meaningful. Adjusting for the variance as a SMD would help inform us if this is so or if these studies were overpowered. This clinical relevance is particularly important as the only significant differences you find in table 3 are for mean differences. Would you have got the same results using standardised mean differences as the effect size for the cardiac parameters?

Tables 3 and 4. I am not clear why you state "NA" in tables 3 and 4 in the publication bias column. Were you for some reason unable to test for this? I also notice that when you did use Egger's test of publication bias there are several meta-analyses which did yield evidence of publication bias with $p<0.05$. I wonder in the absence of any apparent correction for this and apparent lack of testing for it if this undermines the pooled estimates obtained from the meta-analyses.

On a similar theme there is substantial inter-study heterogeneity found as evinced by the large I^2s in tables 3 and 4. I assume you used the DerSimonian-Laird pooled odds ratio when there was between study heterogeneity and similar approaches that incorporate the extra random component associated with between study heterogeneity to obtain confidence intervals for the effect sizes?

Lines 557-558. Can you speculate on which confounding factors between the studies may have contributed to the large amount of between study heterogeneity e.g. sex, age, socio-economic status. It may be for some demographic groups, for example, there are no health benefits to using e.g. E-cigarettes as opposed to NRTs.

I wonder if the number of studies is a confounding factor as your most statistically significant results in table 3 are for the meta-analyses with the three meta-analyses having by far the highest number of studies e.g. heart rate (N=25), SBP (N=23) and DBP (N=23). Does using more studies boost the power in some way of the test of a pooled effect?

I also wonder if studies which use more fine grained measures yielding mean and standard differences of the mean are more powerful as I note none of the relative risk effect sizes, which are based on dichotomous measures as opposed to continuous ones, are significant. Could they have been if a continuous measure had been used?

You should put the p-value for Egger's test in table 4 and the p-value for the effect sizes as you have done in table 3.

**Reviewer**	**2**
**Name**	**Martin, Connor D**

**Affiliation**	**Roswell Park Comprehensive Cancer Center, Health Behavior**
**Date**	**20-May-2025**
**COI**	**None**

Thank you kindly for your invitation to review the manuscript submitted to your journal, titled "Health Impacts of Electronic Nicotine Delivery Systems: An Umbrella Review of Systematic Reviews." Here, the authors have extracted and curated information from a wide range of studies and reviews on the numerous and diverse health effects associated with the use of electronic nicotine delivery systems (ENDS). The authors systematically characterized effects of the use of ENDS on important outcomes including: cardiovascular health; respiratory health; mental health; and exposure to toxicants and known carcinogens. I appreciated the thorough and comprehensive nature of the manuscript, and acknowledge the importance of frequent updates to the literature on these dynamic and dangerous products.

I have two major concerns and one minor concern that I would expect to see addressed before recommending this manuscript be accepted for publication. First, the manuscript presents a perceptible lack of editorial polish. There are an alarming number of inconsistencies in the language and formatting used. Variable use of key terms detracts from presentation. Although this does not detract from the scientific merit of the manuscript, I refer to this as a major rather than minor concern because of how widespread the issue is.

My second major concern is the inclusion of significant content in the Discussion section that is not in the Results section. Numerous sections of the Discussion section expand at length on topics that are not reported on in the Results section. While some extrapolation is expected (essential, even) in a Discussion section, it should follow naturally from content in the Results.

A third concern is minor; the novelty of what this manuscript offers is poorly articulated. Per the introduction, a similar umbrella review was published in 2022. It would improve the manuscript to call attention to what is *new* in this review that has been added to the field. Many of the cited studies have been published since the 2022 review, and I have no doubt that this manuscript adds a novel and valuable contribution, but the specific contribution should be better highlighted.

I present a line-by-line summary of my critiques below. It largely overlaps with the above commentary. Pending significant revisions, I am happy to review a resubmission of this manuscript, which I anticipate will be of significant value to the field once polished.

INTRODUCTION

The introduction leads strongly with a narrative on the use of tobacco, then a paragraph on nicotine. The narrative then turns to ENDS in specific markets with no segue. Discussion of prevalence of vaping should come after the dangers of vaping are established.

Lines 87-88: the assertion that flavors make vaping less harsh is accurate, but one or more citations are needed.

Line 91: "ENDSs" is used rather than the more correct "ENDS," which is used throughout the rest of the manuscript.

Line 94: vaping is misspelled as "vapping."

Throughout: the byproduct of vaping devices is aerosols, not smoke. These are distinct byproducts with differential health consequences, a critical distinction between use of vapes and combustible cigarettes. This should be corrected throughout.

Line 101: use of the abbreviation FDA should be given fully at first use (e.g., Food and Drug Administration).

Line 107: again, “ENDSs” should be ENDS. Needs to be updated throughout manuscript.

By the end of the introduction, it is not clear to me what, *specifically*, this umbrella review will offer that others have not already. The content between lines 108-128 suggests that this is one part of a trio of umbrella reviews, with this paper addressing health impacts of ENDS. How is this manuscript going to be different than the Afsar et al. 2022 paper referenced on line 113? It may be beneficial to do something to highlight in the discussion section what content from this analysis is updated and/or was not available when prior reviews were conducted.

METHODS

Justification for the exclusion of non-nicotine ENDS (line 148) should be given. Many of the health effects of ENDS are not restricted to the presence of nicotine. There may be significant limitations on work done with non-nicotine vaping products, but some justification for this decision should be made explicit. The inclusion of this group as a comparator in some cases (e.g., lines 242-243) makes the professed exclusion all the more peculiar.

Table 1: exclusion criteria include dual users of ENDS and tobacco; why? These people make up many regular ENDS users, and dual users may be vulnerable to even greater levels of exposure to toxicants. It may be narratively cleaner to exclude, and such a decision may be acceptable, but justification must be given for such a significant omission. Again, there does appear to be some comparison (e.g., lines 258, 260, 266-267, 270-271) which is helpful. But that is unexpected given how Table 1 presently reads.

The descriptions of the literature search, screening and selection, data extraction, quality assessment, and data synthesis are all acceptable. The methodology does not invite critique.

RESULTS

Line 210, “p” in “P-value” should be lower-case and italicized.

Section on heart rate, lines 249-263 for example, including the time frame studied would be useful. ENDS increased heart rate… for how long? Comparators include users of non-nicotine e-cigarettes, users of nicotine replacement therapies, and never smokers; these are distinct groups. When describing various effects of ENDS, the comparator in a specific comparison should be made explicit (unless effects are being aggregated across comparators? which, if that is what is being done here, is not made very clear).

Measures where differences have clinical implications should be called out explicitly. As in line 256, for example, ENDS use increased heart rate by 1.41 BPM… compared to whom? After how much use? For how long? Is this clinically significant?

Abbreviations are frequently not spelled out on first use (e.g., DPB, MAP, lines 268 and 272, respectively).

Line 277: single word sentence, “Evidence” with no punctuation; was a citation meant to go here?

Authors repeatedly move back and forth between “ENDS” and “e-cigarette” as on line 279 (but also many places elsewhere). There should be consistent terminology.

Lines 292-297: why is this whole section italicized?

In some places, ranges of 95% confidence intervals are given as “# -- #” while in others as “# to #.” The authors should practice consistency in formatting values.

Is there a reason why country of origin is mentioned in some cases but not others? I don’t see that it adds much to the manuscript, unless there is some specific factor that would suggest the country of origin of the paper would affect generalizability.

Line 361: vapers is misspelled as “vapours.”

The terms “ENDS users” and “vapers” are used interchangeably throughout the paper; authors should choose one term for consistency. On a related note; it is advisable though not essential that authors consider person-centered language when discussing the use of nicotine products, consistent with calls within the field (see: Goodwin, R. D., & Walker, L. K. (2022). Time to stop using the word “smoker”: reflecting on the role of language in advancing the field of nicotine and tobacco research. *Nicotine and Tobacco Research*, *24*(12), 1847-1848.).

Line 385: again, “vapours” rather than “vapers” is used.

DISCUSSION

There is an entire subheading of the discussion dedicated to nicotine poisoning, but nothing in the Results section precedes this inclusion. Was this intentional?

The same issue with explosions and burn injuries. Why is this in the Discussion section but not in the Results section?

Some of the language in the Discussion on differences in this umbrella review from previous similar papers could be moved or adapted for inclusion in the Introduction; it was not clear at the onset what the comparative advantage of this manuscript would be.

The utility of ENDS for cigarette smoking cessation is an important topic to address in the conversation on the use of ENDS, but as with previous section of the Discussion, this is outside the scope of what is presented in the Results. This needs to either be added to Results, or removed from Discussion.

VERSION 1 - AUTHOR RESPONSE

Serial Number	Reviewer	Line number given	Comment	Resolved /Comments (New Line number in Italicized format)
1	1	205	Which routine you used in R for the meta-analysis e.g. metafor	We sincerely appreciate your valuable feedback and for highlighting this point. We have included the use of the meta and metafor packages for conducting our meta-analysis, and we've ensured this is clearly specified in the Methods section. *Line 199*
2	-	208	replace the wording with "low heterogeneity" for 0%-40%.	Thank you for the comment. We have replaced the wording as you suggested in the methods section. *Line 202*
3		210-211,<br>Table 3	Rather than a visual inspection of the funnel plots to assess asymmetry of publication bias you should use Duval and Tweedie's (2000) more objective trim and fill procedure which will assess asymmetry and in addition add in any extra studies to any asymmetric plots to give an unbiased effect size.	We are deeply grateful for your insightful and invaluable suggestion. We have now incorporated the Trim and Fill procedure to further assess publication bias, enhancing the robustness of our meta-analysis. Mention is made in methods section. *Line 205*<br>Publication bias is discussed in Table 3. And Table 4.
4		Table 3.	You appear to give the total sample size in the third column. I think you should split this and quote the sample sizes for each group being compared by the effect size e.g. for E-cigarettes and NRT for the Relative Risk in row 1. I also think you should state in the comparison columns of table 3 and 4 in each row which groups are being compared to yield the effect size in that row. You mention which groups are being compared for some rows but not for the majority.	We sincerely appreciate your insightful and valuable feedback. In response, we have revised our manuscript to clearly specify the sample sizes for each group being compared in the third column, such as for E-cigarettes and NRT in relation to the Relative Risk in row 1. Additionally, we have updated Tables 3 and 4 to explicitly state the comparator groups in the first column for all rows, ensuring clarity across all comparisons. Thank you for your thoughtful guidance, which has significantly enhanced the quality and transparency of our presentation

5		Table 3.	Could you explain why you use a mean difference for cardiac parameters in table 3? The usual effect size for a comparisons of means is the standardised mean difference or Cohen's d which you later use for the respiratory parameters and, as such, is more understandable that the raw difference in means as it is scale invariant. It may be the mean differences you have found for the cardiac parameters are too small to be not clinically meaningful. Adjusting for the variance as a SMD would help inform us if this is so or if these studies were overpowered. This clinical relevance is particularly important as the only significant differences you find in table 3 are for mean differences. Would you have got the same results using standardised mean differences as the effect size for the cardiac parameters?	We agree with your comment. Standardized mean difference (SMD) offers greater generalizability. However, we extracted the data directly from the included systematic review where they used mean differences for pooling.<br><br>To use SMD, we would need to access individual primary studies in order to extract means, standard deviations, and sample sizes. Considering these peer reviewed published systematic reviews, we used the second approach (MD). A seminal methodological study comparing SMD and MD from 1000+ meta-analyses have found that there were no significant differences in the results between MD and SMD (Takeshima, N., Sozu, T., Tajika, A., Ogawa, Y., Hayasaka, Y., & Furukawa, T. A. (2014). Which is more generalizable, powerful and interpretable in meta-analyses, mean difference or standardized mean difference?. *BMC medical research methodology*, *14*, 30. https://doi.org/10.1186/1471-2288-14-30) Therefore the results presented are unlikely to be greatly affected by SMD or MD.<br>Consequently, we reported the effect size as presented in the included systematic review (Siddiqi et al.: https://www.sciencedirect.com/science/article/pii/S0146280623001652?via%3Dihub).
6		Table 3,4<br><br>Line 259	I am not clear why you state "NA" in tables 3 and 4 in the publication bias column. Were you for some reason unable to test for this? I also notice that when you did use Egger's test of publication bias there are several meta-analyses which did yield evidence of publication bias with p<0.05. I wonder in the absence of any apparent correction for this and apparent lack of testing for it if this undermines the pooled estimates	Thanks for pointing it out. We apologize for any confusion regarding the "NA" entries in the publication bias column of Tables 3 and 4 and the handling of publication bias in our meta-analyses.<br><br>To address your first point, we reported "NA" in the publication bias column [illegible] outcomes in Tables 3 and 4 where [illegible] fewer than 10 studies were included. This was due to the limited reliabilit[illegible] funnel plots and statistical tests, su[illegible] as Egger's test, for assessing

Commented [DS1]: Need to put these words as well in response somewhere.

Commented [SG2R1]: Included a line at Line 259.
+ Could not find footnote below Tables 3 and 4. I have tried to add one.

			obtained from the meta-analyses.	publication bias with a small number of studies, as these methods may produce unreliable results in such cases. We have now clarified this rationale in the Methods section and added a footnote to Tables 3 and 4 to ensure transparency.<br><br>For the observational studies in Table 4, we relied on pooled data from existing systematic reviews rather than conducting an independent meta-analysis. We have carefully reviewed these reviews for publication bias assessments and incorporated their findings where available, clearly noting this in the table captions and Results section for improved clarity.<br>Trim and fill procedure was performed for necessary corrections.
7			On a similar theme there is substantial inter-study heterogeneity found as evinced by the large I^2s in tables 3 and 4. I assume you used the DerSimonian-Laird pooled odds ratio when there was between study heterogeneity and similar approaches that incorporate the extra random component associated with between study heterogeneity to obtain confidence intervals for the effect sizes?	Thank you for your insightful comment. We agree with your observation regarding the outcomes with odds ratios (ORs) from observational studies. Given the practical challenges of extracting and pooling primary data across such a wide range of outcomes, we opted to collect pooled ORs from the included systematic literature reviews and present them in the table. We have acknowledged this as a limitation in the Discussion section. As you rightly noted, the meta-analyses employed the DerSimonian-Laird method. We also recognize that between-study heterogeneity is an expected consideration in observational studies, and we have addressed this point in the Discussion as well.<br><br>*Line 579-580*
8		557-558.	Can you speculate on which confounding factors between the studies may have contributed to the large amount of between study heterogeneity e.g. sex, age, socio-economic status. It may be for some demographic groups, for example, there are no health benefits to using e.g. E-cigarettes	We found your comment regarding the potential confounding and effect-modifying factors, such as sex, age, and socio-economic status, to be particularly insightful. We agree that our initial statement on confounding was too general, and your point about the possibility of certain demographic groups not benefiting from e-cigarettes

			as opposed to NRTs.	over NRTs is a crucial one that warrants further discussion. You have correctly identified a key limitation of our meta-analysis: the lack of individual-level data to formally test for effect modification. We have revised the limitations section of our manuscript to include a more detailed and speculative discussion of these factors.<br>*Line 561-564*
9		---	I wonder if the number of studies is a confounding factor as your most statistically significant results in table 3 are for the meta-analyses with the three meta-analyses having by far the highest number of studies e.g. heart rate (N=25), SBP (N=23) and DBP (N=23). Does using more studies boost the power in some way of the test of a pooled effect?	We thank you for highlighting this crucial issue. Your inquiry regarding the number of studies as a "confounding factor" and its connection to statistical significance is a fundamental aspect of meta-analysis, and we value your encouragement to elucidate this in our manuscript.<br><br>The quantity of studies does not serve as a confounding factor in the traditional epidemiological context, where a variable is linked to both the exposure and the outcome. The quantity of studies directly influences the statistical power and accuracy of the meta-analytic estimate.<br>In response to your inquiry: Indeed, incorporating additional studies enhances the test's power for a pooled effect.The explanation based on statistics:<br><br>**Enhanced Accuracy:** The meta-analysis enhances the total sample size by aggregating data from various studies, resulting in a reduced standard error for the overall pooled effect size.<br><br>**Increased Statistical Power:** Statistical power refers to the likelihood of identifying a genuine effect when it is present. A smaller standard error, indicating higher precision, results in a narrower confidence interval surrounding the pooled effect. Comparing Meta-Analysis and Individual Studies: A meta-analysis

				possesses greater statistical power compared to any of the individual studies it integrates. The meta-analyses conducted for heart rate, SBP, and DBP included a substantial number of studies, resulting in a significant total sample size. The strong statistical power enabled us to identify a statistically significant pooled effect, despite the modest effect size. The outcomes with fewer studies likely resulted in meta-analyses that were inadequate to identify a small effect, which may account for the non-significant findings.<br><br>**Hence** We have not changed anything regarding this in our manuscript .
10		641-647	I also wonder if studies which use more fine grained measures yielding mean and standard differences of the mean are more powerful as I note none of the relative risk effect sizes, which are based on dichotomous measures as opposed to continuous ones, are significant. Could they have been if a continuous measure had been used?	Thanks for pointing this out . You are spot on pointing out that continuous measures typically provide more statistical power. As you noted, the non-significant findings for our relative risk effect sizes may indeed be a reflection of the inherent loss of information when a continuous variable is dichotomized.<br><br>This brings up a crucial limitation not just of our review, but of the primary literature itself. Many of the previous studies did not consistently document quantifiable variables with fine-grained measures (i.e., means and standard deviations), which necessitated our reliance on dichotomous outcomes for some analyses. This lack of detailed data prevents the kind of powerful meta-analysis that could have provided a more conclusive result.<br><br>This points to a significant gap in the existing research and a clear direction for future studies. As you astutely noted, if continuous data had been available, it is plausible that our results for these outcomes would have been different. We have added a statement

				to our limitations section to reflect that the lack of such data from primary studies implies a crucial area for future research.<br><br>*Line 587-590*
11		Table 3,4	You should put the p-value for Egger's test in table 4 and the p-value for the effect sizes as you have done in table 3.	Thank you for the suggestion, we have now added the p values of the egger test where available in Table 4
12	2	Through out	First, the manuscript presents a perceptible lack of editorial polish. There are an alarming number of inconsistencies in the language and formatting used. Variable use of key terms detracts from presentation. Although this does not detract from the scientific merit of the manuscript, I refer to this as a major rather than minor concern because of how widespread the issue is.	Thanks for pointing these errors throughout the manuscript. We have reviewed the document to weed out such errors and improve the grammar. We have now ensured and nullified any variability in use of key terms.
13		Discussion<br><br>Lines 488-502;<br>Lines 118-129.	inclusion of significant content in the Discussion section that is not in the Results section. Numerous sections of the Discussion section expand at length on topics that are not reported on in the Results section. While some extrapolation is expected (essential, even) in a Discussion section, it should follow naturally from content in the Results.<br>the novelty of what this manuscript offers is poorly articulated. Per the introduction, a similar umbrella review was published in 2022. It would improve the manuscript to call attention to what is new in this review that has been added to the field. Many of the cited studies have been published since the 2022 review, and I have no doubt that this manuscript adds a novel and valuable contribution, but the	We thank you again for pointing this out. Now we have trimmed down our discussion section and kept it related to our results. A section highlighting the novelty of this study has been added.<br>*Line 466- 480.*<br>In the introduction section, we have added how this study has overall improved in scope and what are the additions after the 2022 review.<br>*Lines 113-122.*

			specific contribution should be better highlighted.	
14		87-88	the assertion that flavors make vaping less harsh is accurate, but one or more citations are needed.	Thank you for pointing this out. We have added the references
15		91	“ENDSs” is used rather than the more correct “ENDS,” which is used throughout the rest of the manuscript.	Thank you for the comment. It was due to some typo error. Now we have changed all ENDSs to ENDS
16		94	vaping is misspelled as “vapping.” Throughout: the byproduct of vaping devices is aerosols, not smoke. These are distinct byproducts with differential health consequences, a critical distinction between use of vapes and combustible cigarettes. This should be corrected throughout.	We have corrected the spelling error. We have also corrected the byproduct of ENDS as aerosol as you suggested instead of smoke throughout the manuscript. Thank you for the suggestion
17		Line 101:	use of the abbreviation FDA should be given fully at first use (e.g., Food and Drug Administration).	Thanks for your astuteness to point out this error on our part. We have now corrected it as well as other similar errors.
18		Line 107:	“ENDSs” should be ENDS. Needs to be updated throughout the manuscript.	Thank you for the comment. Now we have changed all ENDSs to ENDS
19		Lines 118-129	By the end of the introduction, it is not clear to me what, specifically, this umbrella review will offer that others have not already. The content between lines 108-128 suggests that this is one part of a trio of umbrella reviews, with this paper addressing health impacts of ENDS. How is this manuscript going to be different than the Afsar et al. 2022 paper referenced on line 113? It may be beneficial to do something to highlight in the discussion section what content from this analysis is updated and/or was not available when prior reviews were conducted.	We have now elaborated as to how our manuscript is more detailed and enhanced in context and different from earlier manuscripts, in the discussion section. In the introduction section, we have added how this study has overall improved in scope and what are the additions after the 2022 review. *Lines 113-122.*

20		148<br><br>151-168	Justification for the exclusion of non-nicotine ENDS	Thank you for your question regarding the exclusion of non-nicotine ENDS. Our decision to focus solely on nicotine-containing products was made to maintain alignment with the review's primary objective of assessing nicotine-related health risks. This approach also helps ensure methodological consistency, as non-nicotine ENDS may have different risk profiles and a more limited evidence base, which could introduce additional variability.<br><br>We have addressed this rationale in the Methods section for transparency. *Lines 146-150.*
21		242-243<br><br>151-168<br><br>573-581	Many of the health effects of ENDS are not restricted to the presence of nicotine. There may be significant limitations on work done with non-nicotine vaping products, but some justification for this decision should be made explicit. The inclusion of this group as a comparator in some cases (e.g., lines 242-243) makes The professed exclusion is all the more peculiar.	Thanks for highlighting this issue. We have made changes accordingly. While describing methods we have now included this statement in inclusion<br><br>"We included studies that investigated the health effects of nicotine-containing electronic nicotine delivery systems (ENDS). Studies exclusively investigating non-nicotine vaping products (NNVP) were excluded from the meta-analysis, as the primary objective of this review was to synthesize evidence on the health effects of ENDS in the context of nicotine delivery and its use in smoking cessation. However, we did include data from studies where a non-nicotine comparator group was used as a control to isolate the effects of nicotine from other components of the aerosol." *Lines 45-150*<br><br>Also, in the limitations section this has been added.<br><br>"A significant limitation of this umbrella review is the intentional exclusion of studies exclusively on non-nicotine vaping products (NNVP). While our analysis focused on the effects of nicotine, it is well-established that many health effects of ENDS can be attributed to non-nicotine components, such as flavoring agents, propylene glycol, vegetable glycerin, and metal particulates. The decision to exclude

				NNVP was based on the review's primary focus on nicotine-related health effects in the context of smoking cessation. As a result, our findings may not fully represent the complete spectrum of health effects from all types of vaping products. Further research is needed to comprehensively characterize the health impacts of NNVP, independent of nicotine."<br>*Lines 551-554*
22		Table 1	exclusion criteria include dual users of ENDS and tobacco; why? These people make up many regular ENDS users, and dual users may be vulnerable to even greater levels of exposure to toxicants. It may be narratively cleaner to exclude, and such a decision may be acceptable, but justification must be given for such a significant omission. Again, there does appear to be some comparison (e.g., lines 258, 260, 266-267, 270-271) which is helpful. But that is unexpected given how Table 1 presently reads.	In this study, we specifically focused on examining the cardiovascular effects of ENDS alone. Including dual users would have confounded our results, as the established cardiovascular effects of tobacco smoking are well-documented in the literature. To ensure we could exclusively attribute any observed effects to ENDS use rather than to concurrent tobacco smoking, we deliberately excluded dual users from our analysis. This approach allows for a clearer understanding of the specific impact of ENDS on cardiovascular parameters.
23		210	"p" in "P-value" should be lower-case and italicized.	We have corrected it. Thank you for the comment
24		249-263	example, including the time frame studied would be useful. ENDS increased heart rate... for how long? Comparators include users of non-nicotine e-cigarettes, users of nicotine replacement therapies, and never smokers; these are distinct groups. When describing various effects of ENDS, the comparator in a specific comparison should be made explicit (unless effects are being aggregated across comparators? which, if that is what is being done here, is not made very clear).	The time frame for cardiovascular measurements varied across studies, ranging from immediately after vaping to 30 minutes or 1 hour after use. This variation in assessment timing contributes to the heterogeneity observed in our findings. We acknowledge that the original systematic reviews did not explicitly specify these time frames, and we have now added this important detail to the manuscript as suggested.<br><br>Regarding comparators, we have clarified in Tables 3 and 4 that most studies used pre- and post-ENDS exposure comparisons within the same participants. We have also explicitly noted these distinctions in our analysis.

25		277	single word sentence, "Evidence" with no punctuation; was a citation meant to go here?<br>Authors repeatedly move back and forth between "ENDS" and "e-cigarette" as on line 279 (but also many places elsewhere). There should be consistent terminology.	Thanks for pointing this out. It was a formatting/typo error, we have now corrected it.<br><br>We have now ensured "ENDS" is used through the manuscript
26		292-297:	why is this whole section italicized?<br>In some places, ranges of 95% confidence intervals are given as "# -- #" while in others as "# to #." The authors should practice consistency in formatting values.	We have corrected the formatting, and ensured only subsections headings are italicized. We have corrected the formatting related to 95% CI and ensured consistency
27		Results	Is there a reason why country of origin is mentioned in some cases but not others? I don't see that it adds much to the manuscript, unless there is some specific factor that would suggest the country of<br>origin of the paper would affect generalizability.	We agree with your comment. We have removed the country of origin, since it does not add any value.
28		361	vapers is misspelled as "vapours."	We have corrected it, and used ENDS users instead of vapers. Thank you for pointing it out
		Throughou t	The terms "ENDS users" and "vapers" are used interchangeably throughout the paper; authors should choose one term for consistency. On a related note; it is advisable though not essential that<br>authors consider person-centered language when discussing the use of nicotine products, consistent with calls within the field (see: Goodwin, R. D., & Walker, L. K. (2022). Time to stop using the word "smoker": reflecting on the role of language in advancing the field of nicotine and tobacco research. Nicotine and Tobacco Research, 24(12), 1847-1848.).	We agree with your comment. We have corrected it, and used ENDS users instead of vapers. We have changed the "word smokers" to 'individual who smoke', where possible
29		Line 385:	"vapours" rather than "vapers" is	We have corrected it.

			used.	
30		Lines 118-129<br><br>Lines 426-443	There is an entire subheading of the discussion dedicated to nicotine poisoning, but nothing in the<br>Results section precedes this inclusion. Was this intentional? The same issue with explosions and burn injuries. Why is this in the Discussion section but not in the Results section?<br><br>Some of the language in the Discussion on differences in this umbrella review from previous similar<br>papers could be moved or adapted for inclusion in the Introduction; it was not clear at the onset<br>what the comparative advantage of this manuscript would be.<br>The utility of ENDS for cigarette smoking cessation is an important topic to address in the conversation on the use of ENDS, but as with previous section of the Discussion, this is outside the scope of what is presented in the Results. This needs to either be added to Results, or removed from<br>Discussion.	Thanks for pointing out this un-intentional error. We have now placed a separate section of nicotine poisoning along with explosion and burn injuries to the Results.<br>*Lines 394-420*<br><br><br>We have considered your feedback carefully and agree with your points. We have now revised the manuscript accordingly.<br><br>**Regarding your first comment** about the content in the Discussion being better suited for the Introduction, we completely agree. Our intention was to highlight the novelty of our umbrella review, and we agree that this should be made clearer at the onset. We have revised the Introduction to more explicitly state the comparative advantage of our manuscript over previous similar papers, setting the stage for the reader from the very beginning.<br><br>*Lines 113-122*<br><br>**Regarding your second comment** about the discussion of ENDS utility for smoking cessation being outside the scope of our Results, you are absolutely correct. We acknowledge that Results section in this manuscript does not present data on cessation outcomes. The primary reason for its inclusion in discussion section is to provide a broader view to our readers about ENDS. To address your point without removing this important section of discussion, we have included an introductory para in Discussion before delving on the cessation effect, "**ENDS use in the Context of Smoking Cessation**" This new section serves to contextualize our results by referencing the broader literature on cessation, making it clear that we are not presenting new data on this topic in this manuscript. This ensuring that the discussion remains grounded in the

				scope of our results while still providing necessary context for clinical interpretation.<br><br>*Lines 489-508*<br><br>We believe these changes will significantly improve the manuscript's structure and clarity. Thank you again for your valuable input.

VERSION 2 - REVIEW

**Reviewer**	**2**
**Name**	**Martin, Connor D**
**Affiliation**	**Roswell Park Comprehensive Cancer Center, Health Behavior**
**Date**	**08-Aug-2025**
**COI**	

All of my concerns relating to the prior submission have been addressed satisfactorily. I am recommending a decision of 'Accept' to the journal.
